# Synthesis, Characterization, Biological Evaluation, DFT Calculations, and Molecular Docking Study of Transition Metal Complexes Derived From a Schiff Base Ligand

**DOI:** 10.1002/cbdv.202500940

**Published:** 2025-06-26

**Authors:** Ibrahim Waziri, Olaide O. Wahab, Grema A. Mala, Musa B. Ismaila, Usman Umaru, Mostafa S. Abd El‐Maksoud, Ibrahim M. Wakil, Tunde L. Yusuf

**Affiliations:** ^1^ Department of Chemical Sciences University of Johannesburg Johannesburg South Africa; ^2^ Department of Pure and Applied Chemistry, Faculty of Physical Sciences University of Maiduguri Maiduguri Nigeria; ^3^ Department of Chemistry, School of Secondary Education, Science Programmes Federal College of Education Kontagora Kontagora Niger Nigeria; ^4^ Department of Pharmacology and toxicology Al‐Azhar University Assiut Egypt; ^5^ Department of Chemistry, Faculty of Natural and Agricultural Sciences University of Pretoria Pretoria South Africa

**Keywords:** bacterial resistance, DFT, docking, metal complexes, oxidative stress, Schiff bases

## Abstract

The escalating global challenge of antibiotic resistance amid rising oxidative stress emphasizes the critical necessity for innovative antimicrobial strategies. This study, reported synthesis of Schiff base ligand, (*Z*)‐2‐[(4‐nitrobenzylidene)amino]phenol (HL), derived from *c*ondensation between 2‐aminophenol and 4‐nitrobenzaldehyde, and it Co(II), Ni(II), Cu(II), and Zn(II) complexes. The compounds were characterized using NMR, FTIR, UV−Vis, MS, TGA, SEM‐EDX, and elemental (CHN) analysis. In addition, solid‐state structure of HL was obtained using single‐crystal x‐ray diffraction. The characterizations show that the ligand coordinated to the metal ions through the oxygen and nitrogen atoms of phenolic and imine moieties, resulting in a homoleptic mononuclear complexes of the form ML_2_, in which, M represents cobalt, nickel, copper, or zinc, and L stands for the ligand. Stability studies using time‐dependent UV−Vis spectroscopy in a 5% DMSO solution showcases the stability of the complexes with constant stability (*K*) as 3.76 × 10^3^, 3.51 × 10^3^, 3.30 × 10^3^, and 2.88 × 10^3^ for NiL_2_, CuL_2_, CoL_2_, and ZnL_2_, respectively. Antibacterial and antioxidant activities were evaluated against selected bacteria and DPPH radical, with the complexes outperforming the ligand. Notably, Ni(II) complex showed superior activity, with MIC value as low as 3.9 µg/mL against *Bacillus subtilis* and IC_50_ values of 2.3 µM on DPPH radical. DFT calculations at the B3LYP/Def2TZVP level supported the experimental results and provided additional insight into the geometry of the complexes. Molecular docking further validated the biological study results, providing insights into the mechanisms of action and binding affinities against the receptor.

## Graphical abstract

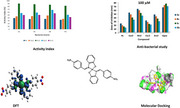

## 1 Introduction

In recent years, escalating bacterial resistance to antibiotics [1, 2] and recurring oxidative stress‐related disorders have underscored the pressing need for novel therapeutic agents with potent antioxidant properties [3, 4]. Schiff bases have emerged as promising candidates due to their remarkable chelating ability, particularly when complexed with transition metal ions [5, 6]. This has led to significant interest in enhancing the chemical structures and biological activities of metal complexes derived from Schiff bases [7, 8], especially those incorporating cobalt, nickel, copper, and zinc, renowned for their antibacterial and antioxidant properties against a spectrum of pathogenic bacteria [9]. Mechanistic studies have unveiled the multifaceted antibacterial actions of these metal complexes [10], including membrane disruption, enzyme inhibition, and interference with bacterial DNA replication [11]. Incorporating metal ions into Schiff base complexes significantly boosts their antibacterial efficacy, potentially reducing the risk of resistance development [12]. Furthermore, oxidative stress, implicated in various diseases like cardiovascular disorders, neurodegenerative conditions, and cancer, underscores the importance of antioxidant interventions [13, 14]. Metal complexes derived from Schiff bases exhibit promising antioxidant capabilities, acting as scavengers of reactive oxygen species (ROS) and inducers of endogenous antioxidant defenses [15]. Transition metal ions within these complexes endow them with redox‐active properties, enabling effective neutralization of harmful free radicals to mitigate oxidative damage [16, 17]. While there is a growing interest in metal complexes derived from Schiff bases for their antibacterial and antioxidant properties, along with existing literature reports on the ligand, few studies have explored their potential as dual‐therapeutic agents, acting simultaneously as antioxidants and antibacterial. In light of the increasing challenges posed by bacterial resistance and the prevalence of oxidative stress‐related disorders, the development of multi‐therapeutic strategies becomes imperative.

In this study, we present the synthesis of metal complexes of cobalt, nickel, copper, and zinc derived from Schiff bases, designed to function as dual‐therapeutic agents combating both bacterial infections and free radicals. Our research endeavors to contribute to the quest for innovative therapeutic solutions addressing the pressing issues of bacterial resistance and the complications arising from oxidative stress‐related diseases.

## 2 Experimental

### 2.1 Chemicals and Instrumentation

For this study, we utilized analytical‐grade chemicals and reagents obtained from Merck Pty Ltd. These materials were used without additional purification. The chemicals employed in this research included 2‐aminophenol, benzaldehyde, sulfuric acid, nitric acid, nickel acetate tetra hydrate, copper acetate, zinc acetate dihydrate, cobalt acetate tetra hydrate, ethanol, methanol, dichloromethane, hexane, and ethyl acetate. The elemental compositions (carbon, hydrogen, and nitrogen) of these compounds were determined using a VarioElementar III microbe CHNS analyzer. The metal content of the complexes was measured using a Spectro Arco FSH12 inductively coupled plasma mass spectrometer. Infrared spectra were recorded using a Tensor 27 Bruker and PerkinElmer FT‐IR spectrometer BX, covering the range of 4000–400 cm^−1^. Electronic absorption spectra were obtained using a Shimadzu UV–Vis 1800 spectrophotometer in DMSO at room temperature, spanning the range of 800–200 nm. The ^1^H and ^13^C NMR spectra were acquired on a Bruker 500 and 125 MHz spectrometer, respectively, at room temperature. Chemical shifts are reported in parts per million (ppm), relative to tetramethylsilane as the internal standard for both ^1^H and ^13^C NMR. Mass spectra were acquired using a Waters Acquity UPLC Synapt G2 HD mass spectrometer. Thermogravimetric analyses (TGAs) were performed on a TGA‐Q600 thermoanalyzer using a heating rate of 10°C/min under a nitrogen flow of 20 mL/min, from room temperature to 800°C. Powder x‐ray diffraction (PXRD) data were collected using a Rigaku Miniflex 600 Benchtop diffraction instrument operating at 40 keV, 15 mA, with Cu‐Kα radiation (*λ* = 1.5418 Å) over a 2*θ* range of 0°C–80°C at room temperature.

#### 2.1.1 Synthesis of (*Z*)‐2‐[(4‐Nitrobenzylidene)Amino]Phenol

**SCHEME 1:**
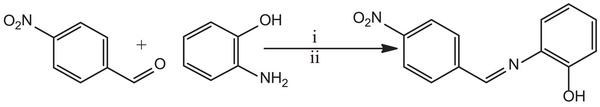
Synthesis of (*Z*)‐2‐((4‐nitrobenzylidene)amino)phenol (HL); i = CH_3_OH/HCOOH; ii = RT, 6 h.

A solution of 4‐nitrobenzaldehyde (0.510 g, 3.30 mmol, 1 eq) in 10 mL of methanol was prepared, to which a solution of 2‐aminophenol (0.360 g, 3.30 mmol, 1 eq) was added, along with three drops of formic acid. The resulting mixture was stirred at room temperature for 6 h, which resulted in the formation of precipitate. Afterward, the precipitate was filtered, washed with diethyl ether, and dried to obtain the final product, (*Z*)‐2‐[(4‐nitrobenzylidene)amino]phenol (HL), as depicted in Scheme 1. Yield: 0.343 g (43.0%); m.p.: 52–56°C; ^1^H NMR (500 MHz, DMSO‐*d*_6_): *δ*_H_ (ppm) = 6.87 (t, 1H, *J* = 7.5 Hz, Ar), 6.93 (d, 1H, *J* = 8.0 Hz, Ar), 7.15 (t, 1H, *J* = 8.0 Hz, Ar), 7.30 (d, 1H, *J* = 7.5 Hz, Ar), 8.30 (d, 2H, *J* = 8.5 Hz, Ar), 8.35 (d, 2H, *J* = 8.5 Hz, Ar), 8.90 (s, 1H, HC═N), 9.23 (s, 1H, OH); ^13^C NMR (125 MHz, DMSO‐*d*_6_): *δ*_C_ (ppm) = 116.3, 119.3, 119.4, 123.8 (2C), 128.5, 129.7 (2C), 136.9, 142.0, 148.6, 151.7, 156.9 (HC═N); IR_ATR_: *v*_max_ (cm^−1^): *v*_(O─H)_ = 3020, *v*_(C═N)_ = 1750, *v*_(NO)asy_ = 1365, *v*_(NO)sy_ = 1226; UV−visible (DMSO, 10^−3^ M): *λ*_max_ (nm): 263 (π→π*), 340 (n→π*); CHN Anal. Calculated for C_13_H_10_N_2_O_3_: C, 64.46; H, 4.16; N, 11.56; found: C, 64.43; H, 4.15; N, 11.53; HRMS–ESI, *m*/*z* [M + H]^+^: Calculated for C_13_H_10_N_2_O_3_ = 243.0770; found = 243.0844.

### 2.2 Synthesis of the Complexes

The solution of HL (0.20 g, 0.825 mmol, 2 eq) in 10 mL of dichloromethane was mixed with one equivalent each of the metal salts; Co(OAc)_2_·4H_2_O (0.10 g, 0.413 mmol, 1eq), Ni(OAc)_2_·4H_2_O (0.10 g, 0.413 mmol, 1 eq)), Cu(OAc)_2_ (0.08 g, 0.413 mmol, 1 eq), or Zn(OAc)_2_·2H_2_O (0.09 g, 0.413 mmol, 1 eq) in 10 mL of methanol, respectively, in separate reaction flask. The solutions of the mixtures were stirred at room temperature for 6 h. After that, the precipitate formed was filtered, washed with methanol and ether or concentrated (in the absence of precipitate) and dried. The solid product was further dissolved in dichloromethane and layered with hexane for crystal growth. The reaction procedure is illustrated in Scheme S2.

#### 2.2.1 Bis(*Z*)‐2‐[(4‐Nitrobenzyllidene)Amino]Phenolato Cobalt(II)

Yield: 0.3438 g (68.8%); m.p.: 226–228°C; IR_ATR_: 2890, 1612, 1520, 1452, 1309, 823, 554, 428 cm^−1^; UV−Vis: (DMSO, 10^−3^ M): *λ*_max_ (nm): 257 (π→π*), 350 (n→π*), 450 (d‐d, transition); CHN Anal. calculated for C_26_H_18_CoN_4_O_6_: C, 57.68; H, 3.35; N, 10.35; found: C,57. 64; H, 3.33; N, 10.33; HRMS–ESI, *m*/*z* [M + H]^+^: calculated for C_26_H_18_CoN_4_O_6_ = 542.0637; found = 542.2303.

#### 2.2.2 Bis(*Z*)‐2‐[(4‐Nitrobenzyllidene)Amino]Phenolato Nickel(II)

Yield: 0.231 g (48.8 %); m.p.: 148–152°C; ^1^H NMR (500 MHz, DMSO‐*d*_6_): *δ* (ppm) = 6.84 (t, 1H, *J* = 7.5 Hz, Ar), 6.93 (d, 1H, *J* = 8.0 Hz, Ar), 7.13 (t, 1H, *J* = 8.0 Hz, Ar), 7.28 (d, 1H, *J* = 8.0 Hz, Ar), 8.28 (d, 2H, *J* = 8.5 Hz, Ar), 8.35 (d, 2H, *J* = 8.5 Hz, Ar), 8.95 (s, 1H, HC═N); ^13^C NMR (125 MHz, DMSO‐*d*_6_): *δ* = 106.5, 123.4, 123.7 (2C), 126.6 (2C), 128.1, 132.4, 135.4, 146.0, 147.0 (C─O), Ar; 170.3 (HC═N); IR_ATR_: 2900, 1666, 1509, 1332, 749, 503, 434 cm^−1^; UV−Vis: (DMSO, 10^−3^ M): *λ*_max_ (nm): 250 (π→π*), 350 (n→π*), 460 (d‐d, transition); CHN Anal. calculated for C_26_H_18_N_4_NiO_6_: C, 57.71; H, 3.35; N, 10.35; found: C, 57.69; H, 3.32; N, 10.34; HRMS–ESI, *m*/*z* [M + H]^+^: Calculated for C_26_H_18_N_4_NiO_6_ = 542.1457; found = 542.2307.

#### 2.2.3 Bis(*Z*)‐2‐[(4‐Nitrobenzyllidene)Amino]Phenolato Copper(II)

Yield: 0.266 g (50.2 %); m.p.: 160–164°C; IR_ATR_: 2927, 1650, 1509, 1385, 1160, 754, 554, 423 cm^−1^; UV−Vis: (DMSO, 10^−3^ M): *λ*_max_ (nm): 308 (π→π*), 390 (n→π*), 648 (d‐d, transition); CHN Anal. calculated for C_26_H_18_CuN_4_O_6_: C, 57.19; H, 3.32; N, 10.26; found: C, 57.16; H, 3.30; N, 10.25; HRMS–ESI, *m*/*z* [M]^+^: Calculated for C_26_H_18_CuN_4_O_6_ = 545.0522; found = 545.0084.

#### 2.2.4 Bis(*Z*)‐2‐[(4‐Nitrobenzyllidene)Amino]Phenolato Zinc(II)

Yield: 0.247 g (58.8 %); m.p.: 163–166°C; ^1^H NMR (500 MHz, DMSO‐*d*_6_): *δ* (ppm) = 5.96 (t, 1H, *J* = 7.0 Hz, Ar), 6.21 (d, 1H, *J* = 8.5 Hz, Ar), 6.66 (t, 1H, *J* = 7.0 Hz, Ar), 6.96 (d, 1H, *J* = 7.5 Hz, Ar), 7.92 (d, 2H, *J* = 8.5 Hz, Ar), 8.20 (d, 2H, *J* = 8.5 Hz, Ar), 10.81 (s, 1H, HC═N); ^13^C NMR (125 MHz, DMSO‐*d*_6_): *δ* = 116.0, 119.1, 121.0, 121.7, 125.6, 128.1, 128.7, 130.8, 131.1, 133.1; 134.6; 145.0, (Ar); 188.9 (HC═N); IR_ATR_: 2950, 1685, 1442, 1373, 1203, 524, 455 cm^−1^; UV−Vis: (DMSO, 10^−3^ M): *λ*_max_ (nm): 263 (π→π*), 350 (n→π*), 401 (LMCT); CHN Anal. Calculated for C_26_H_18_N_4_O_6_Zn: C, 57.00; H, 3.31; N, 10.23; found: C, 56.76; H, 3.30; N, 10.21; HRMS–ESI, *m*/*z* [M]^+^, calculated for C_26_H_18_N_4_O_6_Zn = 548.8323; found = 548.5062.

### 2.3 Single‐Crystal x‐ray Diffraction Analysis

Attempts to obtain single crystals suitable for data collection for all the compounds, except for the ligand, HL, were unsuccessful. However, the ligand, HL, was successfully obtained in methanol through a slow evaporation process that took 72 h. The crystallographic data of the ligand, HL, was collected at 293 K using an APEXII instrument with Mo Kα (*λ* = 0.71073) radiation. The collected frames were processed using Bruker SAINT for integration [18]. Subsequently, absorption effects were reduced using SADABS [19], and the structures were solved using SHELXT [20]. Refinement of the structures was performed using SHELXL [21]. Non‐hydrogen atoms were refined anisotropically using the least squares method, while hydrogen atoms were placed geometrically and refined using a riding approximation with isotropic displacement parameters 1.2 times (C─H) or 1.5 times (O─H) the Ueq of the parent atom [22]. The crystal structure graphics were generated using Mercury software [23]. Crystal data and details of the refinement are given in Table S1.

## 3 Biological Evaluation

### 3.1 Stability Study in Aqueous Buffer

The stability analysis of the metal complexes and the free ligand was conducted in an aqueous/DMSO mixture. The compounds were dissolved in a solution of 5% DMSO–KH_2_PO_4_ (50 mM, pH 7.5), and their spectra were monitored over time, with spectral acquisition ranging from 250 to 800 nm at 7‐day intervals.

### 3.2 Stoichiometric and Stability Constant Determination

The stoichiometric ratio of M(II) to the ligand in the complexes was determined using Job's continuous variation method, following the techniques reported in the literature [24]. In this method, different volumes (0, 1, 2, 3, 4, 5, 6 cm^3^) of 0.01 M M(II) solutions were successively pipette into seven 50 cm^3^ volumetric flasks. Corresponding aliquots (6, 5, 4, 3, 2, 1, and 0 cm^3^) of the 0.01 M ligand were added, maintaining a constant mole fraction in the solution. The absorbance of each solution was measured at the wavelength of maximum absorbance of the complex, initially determined by scanning wavelengths from 400 to 800 nm. This process was repeated for each mole fraction of the complex at different temperature ranges (30–60°C), with absorbance recorded immediately after mixing. The data obtained were used to determine the stoichiometric ratio and stability constant of the complexes.

### 3.3 Antibacterial Study

The antibacterial activity of the free ligand, its complexes, the standard drug ciprofloxacin, and the carrier solvent dimethyl sulfoxide (DMSO) was evaluated against Gram‐positive bacteria (*Staphylococcus aureus* and *Bacillus subtilis*) and Gram‐negative bacteria (*Pseudomonas aeruginosa* and *Klebsiella pneumoniae*) [25, 26]. The bacterial isolates were uniformly inoculated on agar plates and incubated at 37°C for 24 h. Subsequently, paper discs containing various concentrations (50 and 100 µM) of the standard and synthesized compounds were introduced. The effect of the compounds on the organisms was assessed by measuring the inhibition zone diameter, expressed in millimeters. The experiment was repeated three times, and the results are presented as mean values with standard deviations.

### 3.4 Determination of Minimum Inhibitory Concentration

The minimum inhibitory concentration (MIC) of the synthesized compounds and the control was determined using a modified broth dilution method [27, 28, 29]. To begin, each of the test compounds and the control were dissolved in DMSO and diluted in a twofold manner to create a stock solution concentration range of 512–0.25 µg/mL. From the stock solution, 100 µL was added to each well of a 96‐well micro plate containing 90 µL of broth solution. Subsequently, 10 µL of a bacterial inoculums (1 × 10^6^ CFU/mL) was added to achieve a final concentration range of 250–0.125 µg/mL. The micro plates were covered and incubated at 37°C for 24 h. The MIC was determined visually as the lowest concentration at which no bacterial growth was observed. Ciprofloxacin was used as the positive control, while a mixture of broth and DMSO served as the negative control.

### 3.5 Antioxidant Study

The radical scavenging ability of the synthesized compounds was assessed using a DPPH (2,2‐diphenyl‐1‐picrylhydrazyl) protocol [30, 31]. The ligand, its complexes, and the control (ascorbic acid [AA]) were dissolved in DMSO to various concentrations ranging from 20 to 100 µg/mL. Subsequently, 1 mL of each concentration was mixed with 3 mL of a 0.1 mM solution of the DPPH radical in methanol. The mixture was then incubated in a dark room for 30 min, allowing for the scavenging reaction to occur. After incubation, the absorbance of the solution was measured using a spectrophotometer at a wavelength of 517 nm. This measurement was carried out in triplicate, and the percentage scavenging activity (SA) was calculated.

### 3.6 Computational Study

A comprehensive computational study was carried out using Gaussian 09/GaussView 5.0 [32] to provide quantum mechanical explanations for some of the experimental data and support this work with additional theoretical evidence which are not obtainable experimentally.

Input geometries of the ligand, the metal ions and the derived complexes were prepared on GaussView 5.0 and optimized to their respective global minima in the absence of any constraint, using B3LYP functional [33, 34] with Def2TZVP basis set [35, 36], for describing all atom types present in the modelled species. This combination of functional and basis set is very reliable for theoretical study of metal–ligand complexes [37, 38, 39]. All optimized geometries were confirmed to lack imaginary frequencies. For the Co(II), Ni(II), and Cu(II) complexes (i.e., bis(*Z*)‐2‐[(4‐nitrobenzyllidene)amino]phenolato cobalt(II) [CoL_2_], bis(*Z*)‐2‐[(4‐nitrobenzyllidene)amino]phenolato nickel(II) [NiL_2_], and bis(*Z*)‐2‐[(4‐nitrobenzyllidene)amino]phenolato copper(II) [CuL_2_], respectively), both square planar and tetrahedral geometrical possibilities were investigated to determine the energetically more favorable geometry (i.e., low or high spin) and hence predict the strength of the ligand's field. Global reactivity/stability descriptors (Equations (1), (2), (3), (4)) were computed from the frontier molecular orbital energies of the modelled species [40]. Stability of metal–ligand bonds was predicted via natural bond orbital (NBO) analysis. The thermodynamics of complex formation was assessed in terms of changes in enthalpy (Δ*H*) (Equation 5), entropy (Δ*S*) (Equation 6) and Gibb's free energy (Δ*G*) (Equation 7) at 298.15 K, in concurrence with complexation equation:

$$M^{n+}+xL^{z}\to {\left[ML_{x}\right]}^{n-\left(xz\right)}$$

where M^n+^ represents Co^2+^, Ni^2+^, Cu^2+^, and Zn^2+^ ions, L represents the ligand, *x* is the number of molecules of L with a charge of *z*.

(1) $$\mathrm{Energy}\;\mathrm{gap}\left(\Delta \right)=E_{\mathrm{LUMO}}-E_{\mathrm{HOMO}}$$

(2) $$\mathrm{Global}\;\mathrm{hardness}\left(\eta \right)=\frac{\Delta }{2}$$

(3) $$\mathrm{Global}\;\mathrm{electro}\;\mathrm{neagativity}\left(\chi \right)=-\frac{1}{2}\left(E_{\mathrm{LUMO}}+E_{\mathrm{HOMO}}\right)$$

(4) $$\mathrm{Electrophilicity}\;\mathrm{index}\left(\omega \right)=\frac{\chi^{2}}{2\eta }$$

(5) $$\Delta H_{\mathrm{Complexation}}=H_{\mathrm{Complex}}-\left(H_{\mathrm{Ligand}}+H_{\mathrm{Metal}\;\mathrm{ion}}\right)$$

(6) $$\Delta S_{\mathrm{Complexation}}=S_{\mathrm{Complex}}-\left(S_{\mathrm{Ligand}}+S_{\mathrm{Metal}\;\mathrm{ion}}\right)$$

(7) $$\Delta G_{\mathrm{Complexation}}=G_{\mathrm{Complex}}-\left(G_{\mathrm{Ligand}}+G_{\mathrm{Metal}\;\mathrm{ion}}\right)$$

### 3.7 Molecular Docking Study

A molecular docking study was conducted to investigate the interactions between the lead compound NiL_2_ and target proteins at a molecular level. Crystallographic structures of the target proteins (IDs: 3ttz, 4ddq, 5eix, 6m1j, 5yto, 2cdu) were obtained from the Protein Data Bank. Active residues were identified based on prior literature [41, 42, 43, 44, 45, 46]. Water molecules and co‐crystallized ligands were removed. Protein preparation involved adding missing atoms and polar hydrogen using AutoDock tools [47], followed by Kollman charge assignment and PDBQT file generation. The 3D structures of ciprofloxacin and AA were retrieved from PubChem and optimized using Avogadro software [48]. The NiL_2_ structure was prepared using GaussView 5.0 and optimized with B3LYP functional in conjunction with Def2TZVP. Docking was performed using AutoDock Vina software [49], selecting the pose with the lowest binding energy for further analysis. Ligand–protein interactions were visualized using Discovery Studio Visualizer software [50]. The re‐docking and calculated RMSD values to validate docking precision are presented in Table 1.

**TABLE 1:** Redocking validation of co‐crystallized ligand of the studied proteins.

Protein	PDB ID	RMSD (Å)	Superimposition plot
*Escherichia coli* topoisomerase	1kzn	0.647	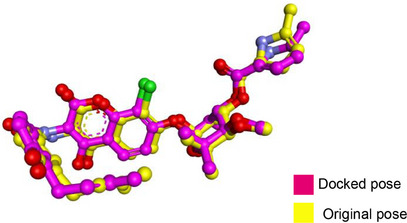
*Klebsiella pneumoniae* topoisomerase	5eix	0.864	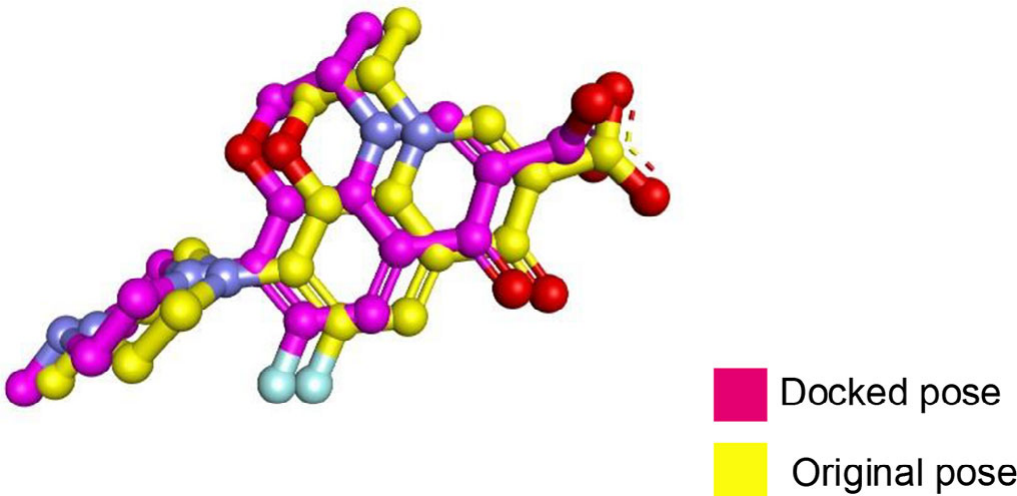
*Staphylococcus aureus* topoisomerase	3ttz	0.574	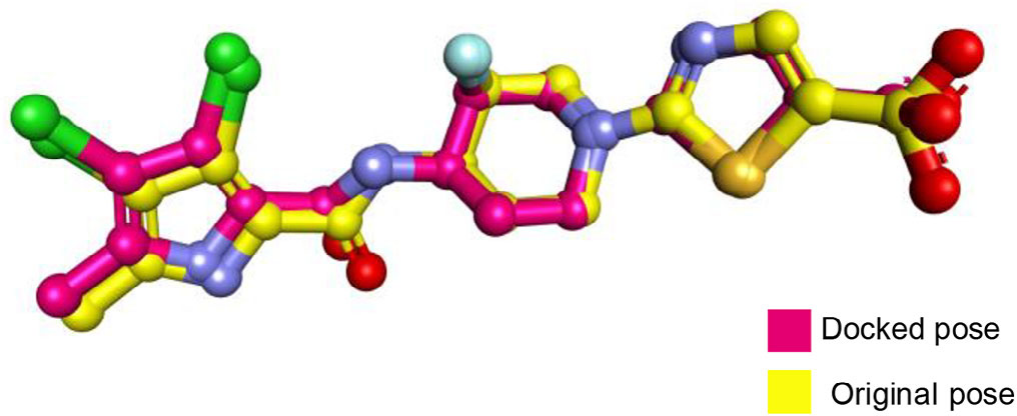

## 4 Results and Discussions

### 4.1 Synthesis

The nitro‐substituted ON donor ligand (HL) was synthesized by treating nitro benzaldehyde, which was obtained through the nitration of benzaldehyde (detail procedure for the nitration is provided in the Supporting Information), with 2‐aminophenol using a methanolic solvent medium in the presence of a catalytic amount of formic acid as a dehydrating agent at ambient temperature. This synthetic procedure facilitated the formation of HL (Scheme 1).

**SCHEME 2:**
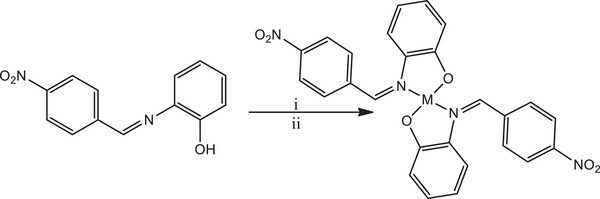
Synthesis of the complexes; i = Co(OAc)_2_·4H_2_O, Ni(OAc)_2_·4H_2_O, Cu(OAc)_2_, or Zn(OAc)_2_·2H_2_O, and ii = CH_2_Cl_2_/CH_3_OH; RT, 6 h.

Subsequently, HL reacted with metal salts of cobalt, nickel, copper, and zinc in a mixture of dichloromethane and methanol, maintaining a 1:2 mole ratio of metal to ligand, at room temperature. This reaction led to the formation of mononuclear homoleptic complexes of the form ML_2_ (Scheme 2).

Both the ligand and its complexes exhibited stability in the presence of air and moisture, and they were soluble in polar solvents. Conductivity studies were conducted on the complexes in 10^−3^ M solutions of DMSO to gain insight into their electrical properties. The measured conductivity values ranged from 6.48 to 10.23 Ω^−1^ cm^2^ mol^−1^, indicating that the complexes exhibited weak electrolyte properties [51, 52]. The complexes displayed higher melting points compared to the ligand. This observation could be attributed to the formation of new compounds with distinct physical and chemical properties, a pointer to the formation of the complexes. The ligand and its complexes were subjected to various spectroscopic characterizations to elucidate their structure as detailed discussed below.

### 4.2 Characterization of the Ligand and Its Complexes

#### 4.2.1 ^1^H and ^13^C NMR Spectra

The spectra of the free ligand (HL), as detailed in Supporting Information (Figures S6 and S7), reveal characteristic signals consistent with its proposed structures. Notably, the azomethine (─CH═N) proton, a key diagnostic functional group confirming Schiff base formation, appears as a singlet at *δ* 8.90 ppm [53], while the hydroxyl proton (─OH) resonates as a singlet at *δ* 9.23 ppm [39, 54]. Aromatic protons exhibit resonance in the range of *δ* 6.85–8.36 ppm, displaying diverse splitting patterns reflective of their distinct chemical environments, accounting for all protons in the molecule. The phenolic proton's downfield appearance relative to the azomethine proton arises from the electron‐withdrawing effect of oxygen atoms, leading to decreased electron density around the proton's nucleus and subsequent deshielding by moving downfield. The ^13^C NMR spectrum of the ligand (Figure S7) displays peaks at *δ* 156.9 and 151.7 ppm from the azomethine carbon and aromatic carbon directly linked to the hydroxyl group, respectively. Upon coordination, the spectra of NiL_2_ and bis(*Z*)‐2‐[(4‐nitrobenzyllidene)amino]phenolato zinc(II) (ZnL_2_), supplementary information (Figures S21, S22, S37, and S38) do not exhibit signals from the hydroxyl proton due to deprotonation and subsequent coordination of the phenolate oxygen to the metal ions, aligning with findings by Kargar et al. [55]. Furthermore, the signal from the azomethine proton shifts to *δ* 8.95 and 10.80 ppm compared to the ligand's spectrum at 8.90 ppm, indicative of nitrogen atom involvement in coordination, consistent with existing literature reports [56]. A summary of the spectral data is provided in Table 2.

**TABLE 2:** ^1^H and ^13^CNMR spectral data (*δ*, ppm) of the ligand and its NiL_2_ and ZnL_2_ complexes.

Compounds	^1^H NMR		
	OH	HC═N	Ar‐H
HL	9.23	8.90	6.85–8.36
NiL_2_	—	8.95	6.82–8.30
ZnL_2_	—	10.80	5.94–8.21

	^13^C NMR		
	C═N	C─O	Ar‐C
HL	156.9	151.7	116.3–148.6
NiL_2_	170.3	147.4	123.4–146.0
ZnL_2_	188.9	160.1	116.0–145

#### 4.2.2 IR Spectra

The vibrational stretching frequencies associated with the functional groups in both the ligand and the complexes were investigated within the range of 4000–400 cm^−1^ in the infrared spectrum. The corresponding spectra are presented in Figures S8, S14, S23, S30, and S38. In the spectrum of the ligand (Figure S8), a weak broad band at 3020 cm^−1^, attributed to υ_(O─H)_ stretching vibrations [57], was observed. In addition, a strong and sharp band at 1750 cm^−1^ indicated the stretching vibration due to the υ_(C═N)_ group, while vibrations at 1450, 1365, and 1226 cm^−1^ were assigned to υ_(C–N)_, υ_(NO)asy_, and υ_(NO)sy_, respectively. The spectra of the complexes (Figures S14, S23, S30, and S39) exhibited the absence of the peak associated with υ_(O─H)_ stretching [39]. This absence is attributed to the chelation of the ligand to the metal ions through the oxygen atom post‐deprotonation, corroborating the NMR study findings. The stretching vibration frequency attributed to C═N appeared in the 1612–1685 cm^−1^ region of the complex spectra, as opposed to 1750 cm^−1^ in the ligand, further confirming the involvement of the nitrogen atom in coordination, aligning with the NMR study results. Moreover, noticeable shifts were observed in other stretching bands, likely originating from molecular vibrations following complexation. New stretching bands between 554–523 and 455–428 cm^−1^ were also identified in the complex spectra, assignable to υ_(M─O)_ and υ_(M─N)_ vibrations, respectively. These observations substantiate the coordination through the (OH) and (C═N) groups.

#### 4.2.3 UV−Vis Spectra

The electronic absorption study was performed using a 10^−3^ M sample solution in DMSO. The individual spectrum is shown in supplementary information (Figures S9, S15, S24, S31, and S40). The ligand spectrum exhibited two distinct bands at 289 and 377 nm, attributed to the aromatic moiety and azomethine group, corresponding to π→π* and n→π* transitions, respectively [58]. Similarly, the spectra of the CoL_2_, NiL_2_, CuL_2_, and ZnL_2_ complexes each displayed three absorption bands within the ranges of 250–263, 308–350, and 417–460 nm. These bands were associated with π→π*, n→π*, d→d transitions, and ligand‐to‐metal charge transfer (LMCT) in the case of ZnL_2_. Notably, the d→d transitions observed for CoL_2_ involved ^4^A_2_→^4^T1(P) and ^4^A_2_→^4^T_1_(F), while those for NiL_2_ included ^3^A_2_g(F)→^3^T_1_g(P) and ^3^A_2_g(F)→^3^T_1_g(F), and for CuL_2_, the transitions were identified as ^2^Eg→^2^T_2_g [59, 60, 61]. The decrease in absorption associated with the azomethine group is caused by a decrease in electron density on the nitrogen atom because of its participation in coordination with a metal ion *via* the lone pair of electrons. Furthermore, the formation of all the complexes is confirmed by the appearance of new absorption bands in the visible region of the spectra. However, the absorption bands in the spectra of Co(II), Ni(II), and Cu(II) fall within the near visible region. This can be attributed to the electron‐withdrawing nature of the nitro group and the resulting electronic effects on the ligand structure. The strong electron‐withdrawing property of the nitro group disrupts the π‐conjugated system within the ligand, altering the delocalization of electrons and affecting the energy levels of the molecular orbital involved in electronic transitions. This disruption, coupled with the presence of two aromatic rings, enhances the complexity of the ligand structure and influences the interaction with the metal center, ultimately leading to absorptions in the near visible region due to modifications in the ligand field strength and electronic transitions within the metal complex [62, 63].

### 4.3 Thermal Study

The thermal properties of the ligand and its complexes were assessed *via* TGA in a nitrogen atmosphere within a temperature range of 25°C–800°C. The thermographs are provided in the Supporting Information (Figures S10, S16, S25, S32, and S41). The TGA curve of the ligand, HL (Figure S10), exhibited stability between 25°C and 180°C, followed by decomposition from 200°C to 700°C, with a weight loss of 63.41%, representing the organic component of the ligand. The calculated weight loss was 62.39%. The curve indicated further stability from 700°C to 800°C, representing the inorganic residue. The TGA curve of CoL_2_ (Figure S16) displayed three decomposition stages with weight losses of 19.42%, 34.07%, and 31.99%, corresponding to the decomposition of water molecules and the two organic ligands, with calculated weight losses of 19.27%, 33.82%, and 31.55%, respectively. For NiL_2_ (Figure S25), the decomposition curve revealed four stages at 25°C–120°C, 120°C–250°C, 250°C–380°C, and 380°C–400°C with weight losses of 5.63%, 8.75%, 31.09%, and 22.53%, representing the loss of moisture content, organic ligands, and inorganic residue, with calculated weight losses of 5.46%, 8.46%, 30.92%, and 21.85%, respectively.

The TGA curve of CuL_2_ (Figure S32) indicated three decomposition stages at 25°C–200°C, 200°C–320°C, and 320°C–400°C with weight losses of 19.42%, 34.07%, and 31.98%, corresponding to calculated weight losses of 19.23%, 33.62%, and 31.72%, respectively, representing the loss of moisture content, organic ligands, and inorganic residue.

Similarly, the TGA curve of ZnL_2_ (Figure S40) exhibited three decomposition stages at 25°C–180°C with a weight loss of 3.43%, calculated as 3.22%, representing the loss of moisture content. The second and third decomposition stages occurred at 180°C–360°C and 360°C–650°C with weight losses of 20.64% and 41.18%, corresponding to the loss of the organic ligands, with calculated weight losses of 20.32% and 40.87%, respectively.

### 4.4 Description of Crystal Structure

**FIGURE 1:**
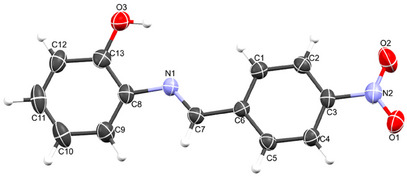
Crystal structure of HL1, with its atoms labeled and numbered.

**FIGURE 2:**
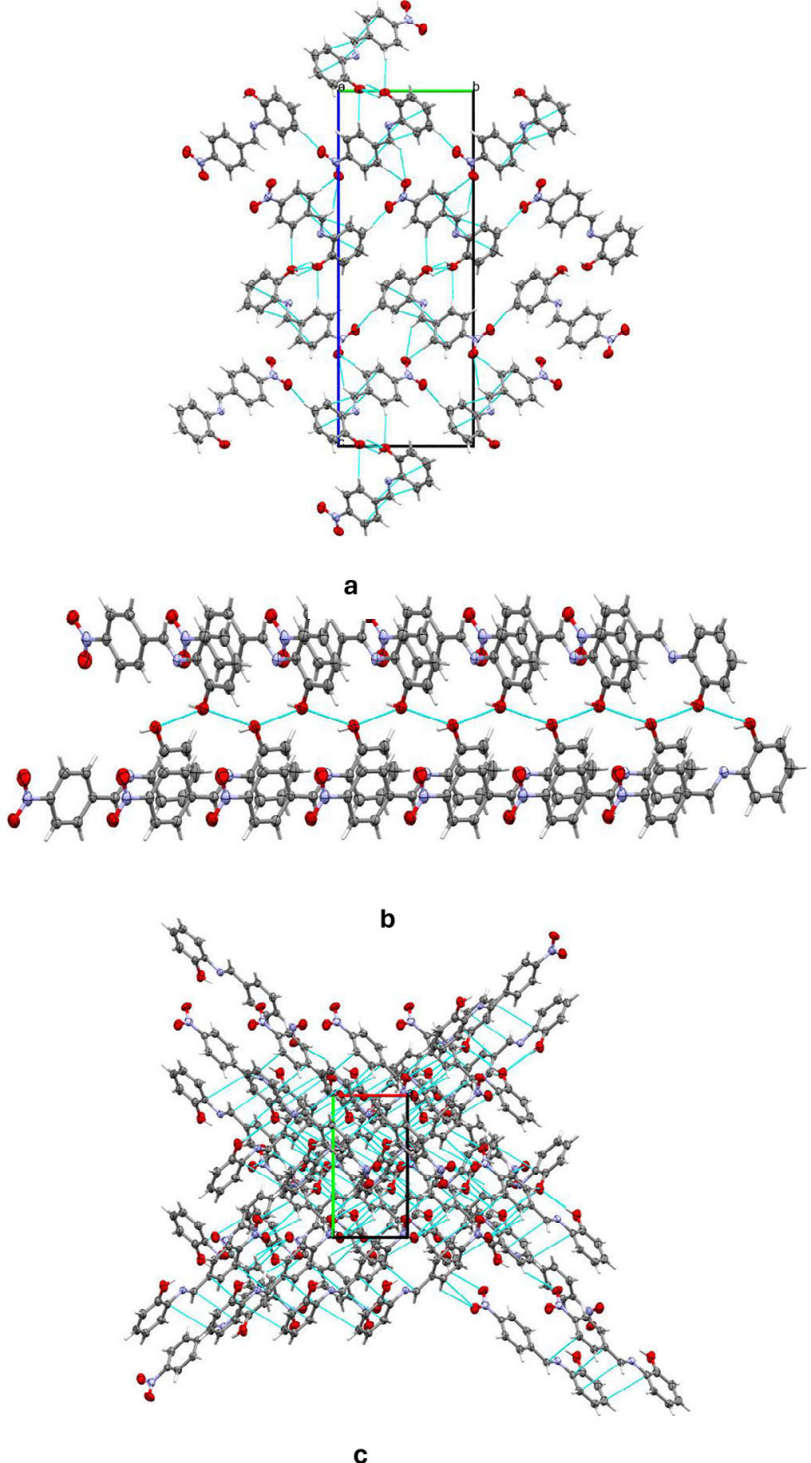
(a) Crystal packing for HL1 viewed along *a*‐axis (b) Representation of intermolecular C─O⋯O (c) expanded bonded interactions.

Despite several attempts, only the ligand, HL, yielded single crystals suitable for data collection. The crystal data reveal that the compound crystallized in the orthorhombic system with the P*2_1_2_1_2_1_* space group, containing one molecule within the asymmetric unit. The molecular structure of the compound, with an atomic numbering scheme, is depicted in Figure 1, while the crystallographic data and final refinement parameters are detailed in Table S1. The imine (C═N; labeled as C7 and N1, respectively) bond distance, a key characteristic of Schiff bases, measures 1.280(5) Å, consistent with C═N bonds in Schiff base compounds. All the atoms of the molecule are approximately coplanar, the greatest deviation from the least squares plane for all atoms, apart from the nitro group, is 0.058(3) Å for C2. The dihedral angle between this plane, and the nitro group is 7.8(5)° while the dihedral angle between the two phenyl rings is 3.9(2)°. The crystal structure of HL is held together by a network of intermolecular hydrogen bonds, specifically C─H⋯O, O─H⋯O, and C─O⋯O types, as illustrated in Figure 2a. These non‐classical hydrogen bonds, which involve O─H donors and O acceptors, create a chain‐like arrangement of neighboring molecules that extends along the crystallographic *a*‐axis (Figure 2b). Furthermore, these molecular chains are interconnected by C─H⋯C interactions, depicted in Figure 2c, forming a complex, multi‐dimensional supramolecular architecture. In greater detail, the C─H⋯O and C─H⋯C hydrogen bonds act as “molecular stitches,” binding adjacent molecules into a cohesive chain. This chain formation is significant as it introduces directional order within the structure along a specific axis, potentially influencing the material's physical properties, such as stability and rigidity. The C─H⋯C contacts provide an additional layer of interaction by linking these chains laterally, establishing a robust, multi‐dimensional network. This network plays a critical role in creating a stable and ordered lattice, where each molecule's position is reinforced by multiple weak yet cooperative intermolecular forces. These supramolecular interactions significantly contribute to the stability of the overall structure and could potentially impact the material's reactivity and behavior under varying conditions.

## 5 Biological Evaluation

### 5.1 Stability Study

The exploration of metal complexes in biological applications requires a nuanced understanding of their stability profiles, a fundamental aspect that underpins their efficacy and safety. Stability studies act as pivotal gateways to comprehend the intricate interplay between metal ions and ligands, illuminating the chemical resilience of these complexes under diverse environmental conditions. These investigations not only pave the way for optimizing experimental protocols but also lay the foundation for elucidating the mechanistic pathways through which these metal complexes exert their biological activities, thereby shaping the trajectory of future research endeavors toward innovative therapeutic interventions and bioinorganic applications. In this study, the synthesized complexes were assessed for stability before evaluating their antimicrobial and antioxidant efficacy. The stability study involved UV spectral analysis at different days intervals in a 5% DMSO‐KH_2_PO_4_ solution. The spectra, as depicted in (Figures S46–S50), reveal no significant changes in the absorption bands of the complexes within 7 days, indicating good stability under the test conditions. Furthermore, the stoichiometric study of the complexation reaction using the continuous variation method revealed a 1:2 ratio (metal to ligand) in all the complexes (Figures S51–S54). Similarly, the stability constant values (*K*) for the complexes were determined. These values exhibited an order of 3.76 × 10^3^, 3.51 × 10^3^, 3.30 × 10^3^, and 2.88 × 10^3^ for NiL_2_, CuL_2_, CoL_2_, and ZnL_2_, respectively, indicating a high stability of the complexes. The significant stability of the chelate can be attributed to resonance effects originating from the benzene rings, facilitating electron delocalization between the ligand–metal π bonding and thus providing additional stability to the ligand. The ligand rings act as stabilizers preventing bond distortion through elongation or compression, thereby contributing to the overall stability of the complex. Subsequently, the biological assays were conducted as outlined below.

### 5.2 Antibacterial Study

The antimicrobial activities of the synthesized ligand and its complexes were screened against two Gram‐positive bacteria, *S. aureus* and *B. subtilis*, and two Gram‐negative bacteria, *K. pneumoniae* and *P. aeruginosa*. The screening was conducted using disc diffusion and broth dilution methods [64, 65] to determine the zone of inhibition and MICs, respectively. The results were compared with those of the standard drug, ciprofloxacin. The zone of inhibition and MIC values are provided in Tables 3, 4, 5. All the compounds exhibited different levels of activity against the tested bacterial strains. Notably, the complexes show greater activity compared to the ligand. Among the complexes, NiL_2_ displayed the highest activity against all tested organisms. The activity displayed by these compounds is superior to that of other Schiff bases containing similar metal ions [66, 67, 68].

**TABLE 3:** In vitro antibacterial activity results at 50 µM of the ligand and its metal complexes.

Compounds	Bacteria			
	SA	BS	KB	PA
HL	12.00 ± 0.800	14.00 ± 0.900	8.50 ± 0.500	7.50 ± 0.500
CoL_2_	15.50 ± 0.465	18.00 ± 0.480	13.30 ± 0.470	9.00 ± 0.820
NiL_2_	25.00 ± 0.820	32.00 ± 0.800	23.50 ± 0.480	22.70 ± 1.300
CuL_2_	20.00 ± 0.470	23.00 ± 0.820	17.00 ± 0.800	14.00 ± 0.900
ZnL_2_	18.50 ± 0.500	21.00 ± 0.950	15.00 ± 0.820	11.00 ± 0.500
Cipro	32.00 ± 0.850	37.60 ± 0.475	29.00 ± 0.950	27.50 ± 0.471
DMSO	—	—	—	—

*Note*: DMSO was included due its usage as a vehicle carrier for the solubilization of the compounds, and results are presented using mean ± SD.

Abbreviations: —, no activity; BS, *Bacillus subtilis*; Cipro, ciprofloxacin; KB, *Klebsiella pneumoniae*; PA, *Pseudomonas aeruginosa*; SA, *Staphylococcus aureus*.

**TABLE 4:** In vitro antibacterial activity results at 100 µM of the ligand and its metal complexes.

Compounds	Bacteria			
	SA	BS	KB	PA
HL	16.50 ± 0.600	17.00 ± 0.450	12.00 ± 0.700	10.50 ± 0.350
CoL_2_	19.00 ± 0.865	22.00 ± 0.810	16.00 ± 0.870	11.50 ± 0.520
NiL_2_	38.00 ± 0.550	42.00 ± 0.600	28.50 ± 0.680	26.00 ± 0.550
CuL_2_	24.00 ± 0.700	28.00 ± 0.750	22.50 ± 0.340	16.00 ± 0.650
ZnL_2_	21.00 ± 0.650	24.00 ± 0.950	18.50 ± 0.520	13.50 ± 0.250
Cipro	42.00 ± 0.750	47.60 ± 0.475	33.00 ± 0.150	31.50 ± 0.871
DMSO	—	—	—	—

**TABLE 5:** Minimum inhibitory concentration (µg/mL) of the ligand and its metal complexes.

Compounds	Bacteria			
	SA	BS	KB	PA
HL	125 ± 0.17	62.5 ± 0.66	125 ± 0.78	125 ± 0.55
CoL_2_	62.5 ± 0.34	15.6 ± 0.08	62.5 ± 0.01	62.5 ± 0.65
NiL_2_	15.6 ± 0.19	3.9 ± 0.34	15.6 ± 0.05	7.8 ± 0.77
CuL_2_	31.2 ± 0.23	7.8 ± 0.67	62.5 ± 0.23	31.2 ± 0.24
ZnL_2_	62.5 ± 0.09	15.6 ± 0.19	62.5 ± 0.54	62.5 ± 0.91
Cipro	3.9 ± 0.23	1.9 ± 0.87	7.8 ± 0.21	3.9 ± 0.33
DMSO	—	—	—	—

Based on the results obtained, the led complex, NiL_2_ exhibit highest activity across all the strains with zone of inhibition of 25.00 ± 0.82 mm for *S. aureus*, 32.00 ± 0.80 mm for *B*. *subtilis*, 23.50 ± 0.48 mm for *K*. *pneumonioe*, and 22.70 ± 1.30 mm for *P*. *aeruginosa* at concentration of 50 µM. comparing this result with the standard at the same concentration, across the strains it can be seen that the control slightly outperformed the NiL_2_ with average zone of inhibition of 5–6 ± 0.92 mm (Table 3). Similarly at concentration of 100 µM, NiL_2_ maintained the highest activity across the tested strains with zone of inhibition of 38.00 ± 0.55 mm, for *S. aureus*, 42.00 ± 0.60 mm for *B*. *subtilis*, 28.50 ± 0.68 mm for *K*. *pneumonioe*, and 26.00 ± 0.55 mm for *P*. *aeruginosa*. Comparative to the control, similar trend as observed at concentration of 50 µM was noted (Table 4).

Furthermore, the MIC result as compiled in Table 5, shows that NiL_2_ has the lowest MIC values with 15.6 µg/mL for *S*. *aureus* and *K. pneumonioe*, 3.9 µg/mL for *B*. *subtilis*, and 7.8 µg/mL for *P. aeruginosa*. The varying antibacterial activities observed among these metal complexes can be attributed to a combination of factors. These include the unique characteristics of the metal ions, with Ni(II) potentially having specific interactions with bacterial targets that enhance their efficacy, while Co(II) exhibits the least activity. The chelation theory suggests that coordination of metal ions to ligands can enhance biological activity, impacting the stability and reactivity of the complexes [69, 70]. Structural features, metal–ligand interactions, steric effects, and electronic properties also play roles in determining the complexes' effectiveness [71]. Ni(II) complex may possess a more favorable geometry and stronger interactions with the ligand, leading to its higher activity, whereas Cu(II) and Zn(II) complexes show moderate efficacy. These combined factors contribute to the observed differences in antibacterial activity among the metal complexes tested against *S. aureus*, *B. subtilis*, *K. pneumoniae*, and *P. aeruginosa*, with Ni(II) complex emerging as the most active.

### 5.3 Activity Index Analysis

In antimicrobial studies, the activity index is a crucial metric used to compare the effectiveness of compounds being tested against a standard drug. This index, determined by assessing parameters like the zone of inhibition or MIC, quantitatively evaluates how well new compounds can inhibit microbial growth. Understanding the activity index is vital in antimicrobial research as it guides the identification of potential candidates for combating microbial infections and aids in the discovery of more effective antimicrobial agents. To calculate the % activity index for the synthesized ligand and its complexes, compared to the standard drug ciprofloxacin against tested bacteria, in vitro study results (zone of inhibition) are utilized using the equation below:

$$\begin{array}{ccc} & & \mathrm{Activity}\;\mathrm{index}\left(\%\right)\\ & & =\left(\frac{\mathrm{Zone}\;\mathrm{of}\;\mathrm{inhibition}\;\mathrm{of}\;\mathrm{the}\;\mathrm{compound}}{\mathrm{Zone}\;\mathrm{of}\;\mathrm{inhibition}\;\mathrm{of}\;\mathrm{the}\;\mathrm{standard}\;\mathrm{drug}}\right)\times 100 \end{array}$$

**FIGURE 3:**
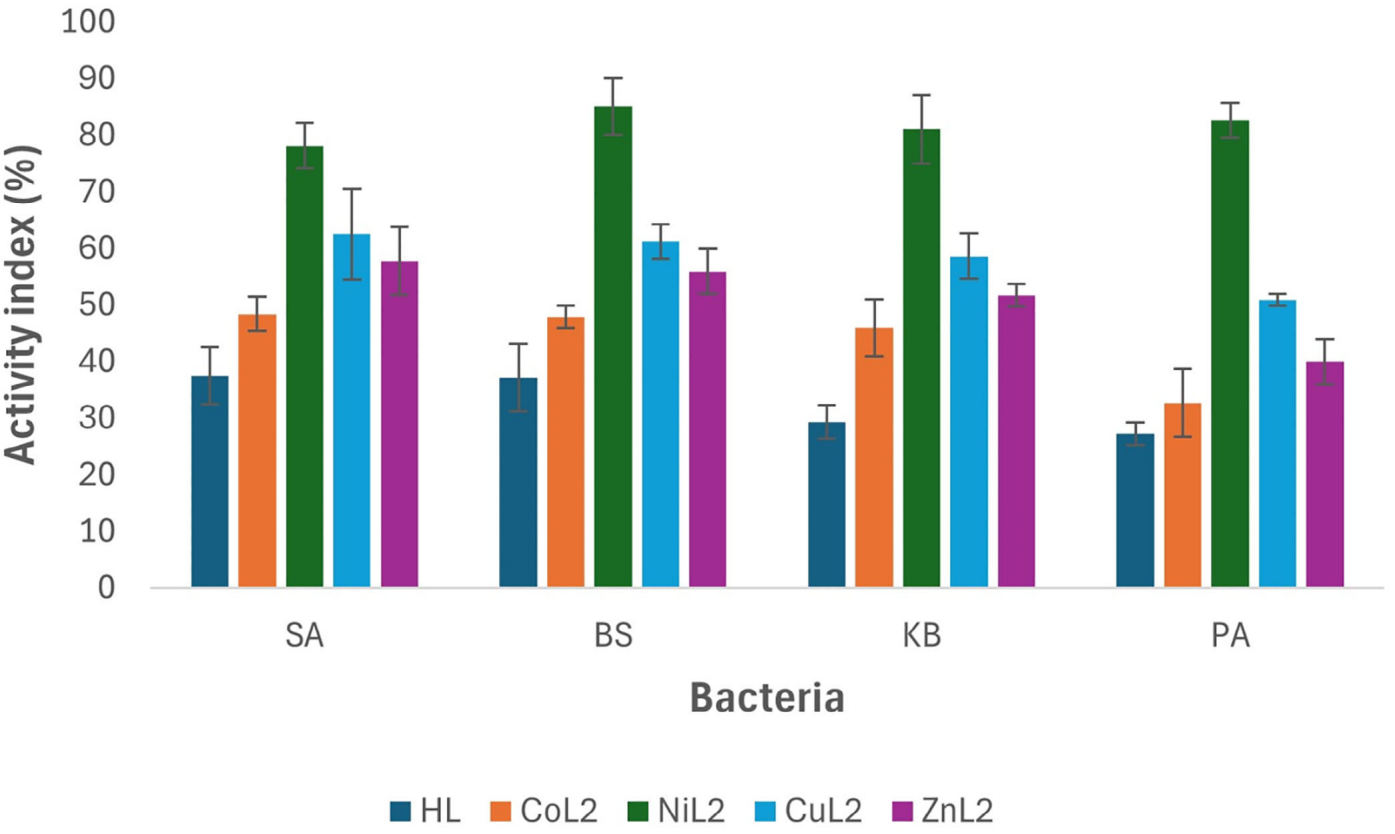
Activity index analysis comparing the compounds to ciprofloxacin at 50 µM against the tested bacteria strains.

**FIGURE 4:**
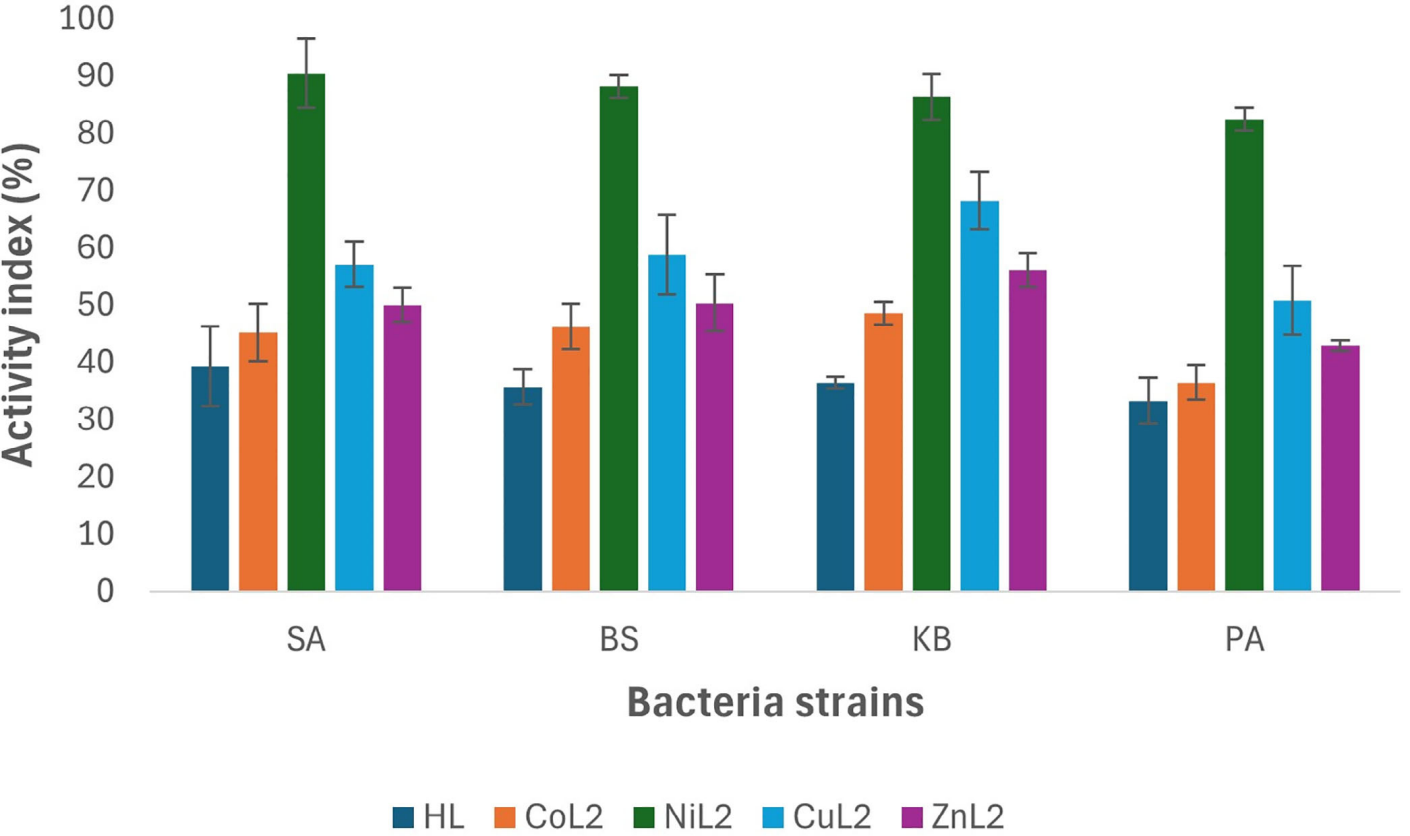
Activity index analysis comparing the compounds to ciprofloxacin at 100 µM against the tested bacteria strains.

The activity index analysis results of the complexes in comparison to ciprofloxacin against tested bacterial strains at 50 and 100 µM concentrations are depicted in Figures 3 and 4. At a concentration of 50 µM, the ligand HL and its CoL_2_ complex exhibited activity indices below 50% across all tested bacteria when compared to the standard drug. The activity index of the ligand ranged from 29.3% to 37.5%, while CoL_2_ complex showed values between 32.7% and 48.4% (Figure 3), indicating their potency is less than 50% compared to the standard drug against the tested bacterial strains. However, CuL_2_ and ZnL_2_ complexes demonstrated activity levels above 50% compared to the standard drug across all tested bacteria, except for *P aeruginosa*, where ZnL_2_ exhibited an activity index of 40% in relation to the standard drug. The activity index of CuL_2_ ranged from 50.9% to 62.5% across the organisms, while that of ZnL_2_ varied from 40% to 57.8% of the standard. Conversely, NiL_2_ displayed higher activity compared to the standard drug than the other complexes against all tested bacterial strains. The activity index of the NiL_2_ complex was 78.1%, 85.1%, 81.0%, and 82.6% of the standard drug on *S. aureus*, *B. subtilis*, *K. pneumoniae*, and *P. aeruginosa*, respectively (Figure 3). A similar trend was observed at a concentration of 100 µM, where the ligand HL and the CoL_2_ complex demonstrated activity indices of less than 50% compared to the standard drug. At this concentration, the activity index of HL ranged from 33.3% to 39.3%, while the CoL_2_ complex exhibited activity indices ranging from 36.5% to 45.2% on the tested bacterial strains. Although these indices were slightly higher compared to those observed at 50 µM, indicating an increase in activity in a concentration‐dependent manner (Figure 3).

Similarly, at this concentration, the CuL_2_ and ZnL_2_ complexes‐maintained activity indices of more than 50% compared to the standard drug, except for *P. aeruginosa* where ZnL_2_ had an activity index of 42.9%. The activity index range of CuL_2_ and ZnL_2_ across the tested bacterial strains was 50.8%–57.1% and 42.9%–56.1%, respectively (Figure 4).

The NiL_2_ complex exhibited sustained higher activity as observed at the 50 µM concentration, with activity indices of 90.5%, 88.2%, 86.4%, and 82.5% compared to the standard drug on *S. aureus*, *B. subtilis*, *K. pneumoniae*, and *P. aeruginosa*, respectively (Figure 4).

The activity index analysis of the compounds indicated that the ligand had the least activity compared to the standard drug, and among the complexes, CoL_2_ was found to be less potent in comparison to the standard drug. NiL_2_ displayed the highest potency among the complexes, with an overall activity index between 80% and 90% on the tested bacteria across the concentrations used (Figure 4). In general, the results demonstrated an increase in activity upon complexation with metal ions as observed in both assays.

### 5.4 Antioxidant Study

The DPPH (2,2‐diphenyl‐2‐picryl‐hydrazyl) free radical scavenging assay was utilized to evaluate the antioxidant potency of the synthesized ligand and its metal complexes. Table 6 illustrates the antioxidant activity of the compounds in comparison to the standard antioxidant, AA. The results indicate that the complexes exhibit higher radical scavenging activity than the ligands but lower than the control, except for NiL_2_ and CuL_2_, which demonstrate superior activity compared to the control (Table 5). This trend aligns with the antimicrobial study results and suggests varying levels of activity among the compounds. The IC_50_ values against the DPPH radical were determined to be 5.20, 2.98, 2.34, 2.68, and 2.88 µM for HL, CoL_2_, NiL_2_, CuL_2_, and ZnL_2_, respectively, while the control exhibited an IC_50_ of 2.85 µM. The complexes' heightened activity compared to the free ligand is due to the chelation effect, electronic modifications enhancing electron transfer, and synergistic effects arising from the metal–ligand complex formation, collectively contributing to their increased antioxidant potency [72, 73]. Similarly, the variation in radical scavenging activity between Ni(II) and Cu(II) complexes compared to Co(II) and Zn(II) complexes, despite originating from the same Schiff base ligand, can be attributed to factors such as distinct metal ion characteristics, metal–ligand interactions, and structural differences influencing antioxidant efficacy. These differences result in Ni(II) and Cu(II) complexes showing superior radical scavenging abilities [74, 75]. Furthermore, the enhanced antioxidant activity observed for the Ni(II) and Cu(II) complexes compared to the standard (AA) can be attributed to their unique electronic structures. Upon coordination with the Schiff base ligand, the metal centers experience a modulation in their redox potentials, favoring electron or hydrogen atom donation to neutralize free radicals such as DPPH. The conjugated system of the Schiff base facilitates electron delocalization, allowing for stabilization of the resulting radical species. In addition, the strong chelation effect of the ligand can suppress the intrinsic pro‐oxidant behavior of free Ni(II) ions by preventing unwanted redox cycling. Therefore, the observed antioxidant activity likely results from a combination of metal–ligand electron delocalization, radical stabilization, and modulation of metal‐cantered redox properties [72].

**TABLE 6:** Antioxidant study activity of the ligand and its metal complexes.

Compounds	DPPH radical scavenging activity					IC_50_ (µM)
	20 µM	40 µM	60 µM	80 µM	100 µM	
HL	12.16 ± 0.53	17.85 ± 0.09	26.92 ± 0.32	38.15 ± 0.63	47.23 ± 0.26	5.20 ± 0.29
CoL_2_	25.69 ± 0.33	36.77 ± 0.13	53.38 ± 0.45	64.92 ± 0.46	70.15 ± 0.51	2.98 ± 0.03
NiL_2_	32.77 ± 0.46	42.00 ± 0.11	66.77 ± 0.33	70.15 ± 0.53	76.46 ± 0.19	2.34 ± 0.06
CuL_2_	31.23 ± 0.08	40.31 ± 0.53	56.31 ± 0.39	67.23 ± 0.61	71.69 ± 0.54	2.69 ± 0.09
ZnL_2_	26.62 ± 0.64	39.08 ± 0.38	54.46 ± 0.18	65.85 ± 0.18	70.46 ± 0.43	2.89 ± 0.31
AA	25.08 ± 0.71	36.00 ± 0.23	55.08 ± 0.45	68.77 ± 0.56	74.46 ± 0.14	2.86 ± 0.43

Abbreviations: AA, ascorbic acid.

## 6 Density Functional Theory Study

### 6.1 Analysis of Optimized Geometries

**FIGURE 5:**
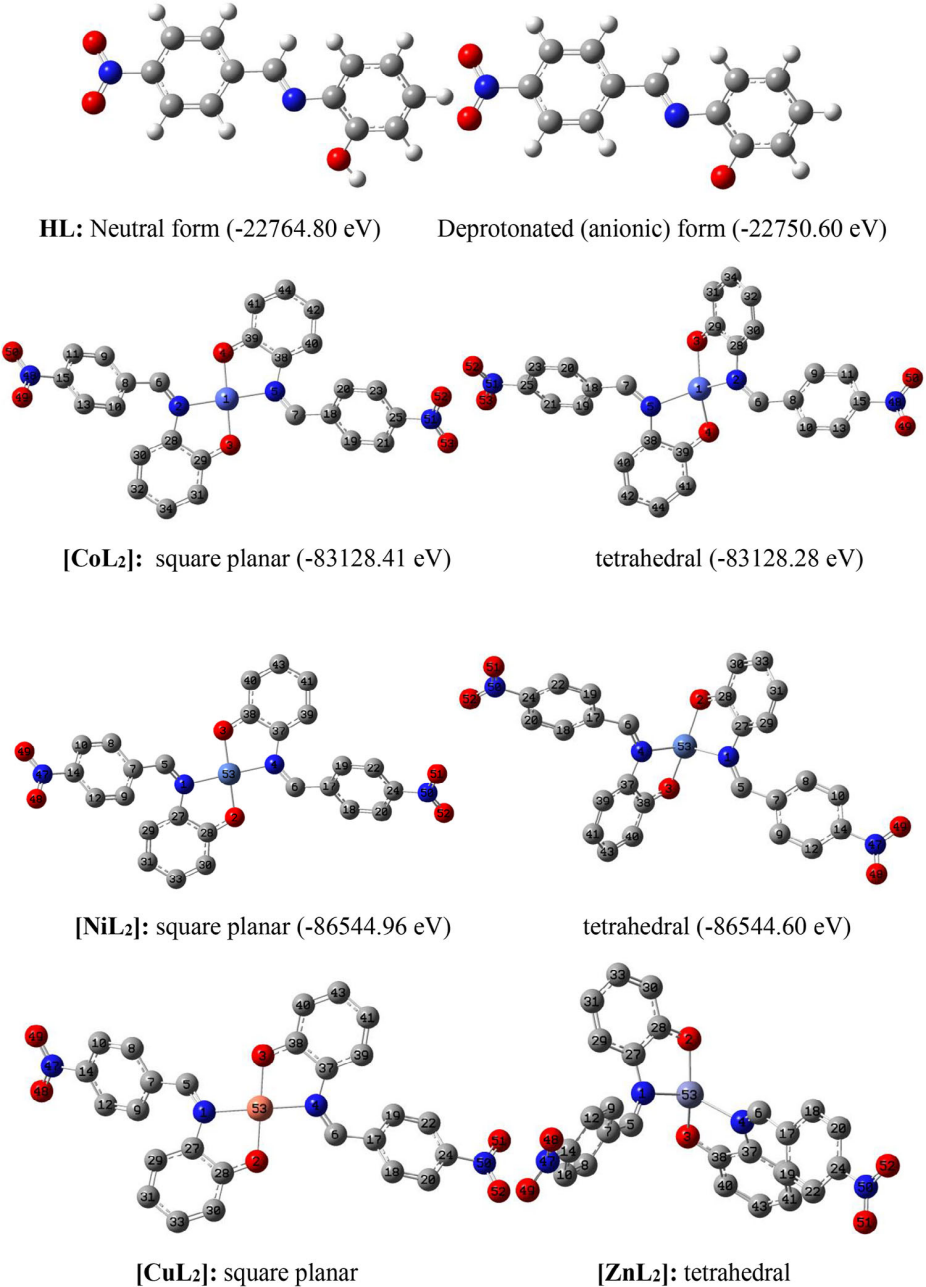
Optimized structures of the ligand and the complexes. Structures of the complexes are presented without hydrogen atoms to increase visibility.

The geometrically optimized models of the ligand and the complexes are shown as Figure 5, alongside their geometrical parameters in Table 7. In this figure, optimized square planar and tetrahedral structures are presented for all but Cu(II) and Zn(II) complexes. Traditionally, tetra‐coordinated Zn(II) complexes are known to have tetrahedral geometry hence, a square planar possibility was not investigated for this complex. In the case of Cu(II) complex, no equilibrium tetrahedral geometry was located throughout the optimization cycle after several attempts. Rather, the input tetrahedral structure kept reverting to the more stable square planar geometry during optimization.

**TABLE 7:** Geometrical parameters of the ligand and its complexes.

Complex	Geometry	M─O (Å)	M─N (Å)	C─O (Å)	─C═N (Å)	∠ O─M─O (°)	∠ N─M─N (°)	∠ N─M─O (°)
HL				1.36	1.27			
[CoL_2_]	SP	1.86	1.95	1.32	1.29	176.87	175.41	94.64
	DT	1.93	2.08	1.31	1.29	153.20	135.97	106.85
[NiL_2_]	SP	1.86	1.93	1.31	1.29	176.88	175.50	94.33
	DT	1.92	2.04	1.31	1.29	159.77	111.94	107.95
[CuL_2_]	SP	1.92	2.03	1.31	1.29	178.69	174.87	96.45
[ZnL_2_]	SDT	1.92	2.12	1.31	1.29	145.41	118.76	114.52

Abbreviations: DT, distorted tetrahedral; SDT, slightly distorted tetrahedral; SP, square planar.

For the Co(II) and Ni(II) complexes, the displayed energy values clearly show that the square planar geometry is energetically more stable than the tetrahedral geometry by −0.1369 and −0.3602 eV, respectively. This implies that the probability of these two complexes favoring a low spin (dsp^2^) configuration is higher than that of a high spin (sp^3^) configuration which suggests the ligand might be more of a strong field ligand. This is further confirmed by the complete absence of the tetrahedral possibility (i.e., sp^3^ configuration) in the copper complex, despite having the same multiplicity (doublet) as the preferred square planar counterpart.

To account for the low/weak vibrational frequencies (V_M─N_ and V_M─O_) of the metal–ligand bonds seen previously observed in the infrared spectral study, we examine the levels of imperfection in the geometries of the complexes and ascertain any structural differences and/or similarities between the square planar and tetrahedral geometries of the cobalt(II) and nickel(II) complexes, assessment of key geometrical properties namely: metal–ligand (M─L) bond lengths and ligand–metal–ligand (L─M─L) bond angles is necessary. In all the complexes, there are two M─O and two M─N bonds formed by the coordination of two ligand molecules to the metal ions, with both pairs forming O─M─O, N─M─N, and N─M─O angles in the coordination sphere. The bond lengths of M─O and M─N are within the known range of metal–oxygen and metal–nitrogen bond lengths in metallic complexes [39, 76]. These bond lengths justify the appearance of weak bands in the fingerprint region of infrared spectra of the complexes (Figures S14, S23, S30, and S38). Similarly, closeness of O─M─O and N─M─N angles to linearity (i.e., 180°), and N─M─O to right angle (90°), confirms the square planar geometry of the cobalt, nickel and copper complexes. On the other hand, the large deviations of N─M─N and N─M─O angles from linearity, and their closeness to 109°5′ (bond angle in a perfect tetrahedron), confirm existence of tetrahedral geometry in zinc complex. Involvement of the C─O and C═N bonds in coordination led to a slight reduction in length of the former but slight elongation of the latter which explains the observed pattern of vibrational frequency between the free and bound ligand.

### 6.2 Chemical Reactivity

**FIGURE 6:**
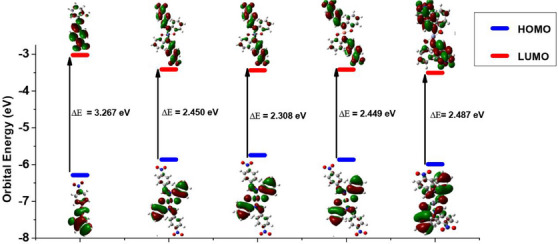
Graphics of frontier molecular orbital electron density isosurfaces of the modeled compounds taken at isovalue 0.02.

The global reactivity parameters, including the highest occupied molecular orbital (HOMO)–lowest unoccupied molecular orbital (LUMO) energy gap (Δ*E*), hardness (*η*), electro negativity (*χ*), and electrophilicity index (*ω*) derived from the HOMO and LUMO energies (*E*_HOMO_ and *E*_LUMO_) of the optimized molecules, are listed in Table 8. Images of HOMO and LUMO electron density isosurfaces are displayed in Figure 6. The *E*_HOMO_ trend suggests that the complexes have a greater tendency to donate electrons to an accepting species than the ligand in the order of [NiL_2_] > [CoL_2_] > [CuL_2_] > [ZnL_2_] > HL. Similarly, the *E*_LUMO_ trend shows that the complexes are more susceptible to an incoming donation than the ligand in the following sequence: [ZnL_2_] > [NiL_2_] > [CuL_2_] > [CoL_2_] > HL. The regions of the complexes involved in donor–acceptor interactions with biomolecules are revealed by the HOMO–LUMO electron density isosurfaces (Figure 6). The HOMO isosurface indicates the part from which electrons can be donated to a biomolecule. This region includes the imine nitrogen atom, the hydroxyl oxygen atom, and a delocalized pi ring network. Conversely, the LUMO isosurface indicates the region to which electrons can be added from a biomolecule, consisting of the electron‐deficient nitro‐substituted phenyl component of the complex. The HOMO–LUMO gap of the complexes is higher than that of the free ligand. The larger HOMO–LUMO gap values in the complexes compared to the ligand could contribute to their enhanced biological activity by providing increased stability, lower reactivity, altered electronic properties, and potentially stronger binding affinity with biological targets.

**TABLE 8:** Molecular orbital‐based reactivity parameters and dipole moments of the synthesized compounds.

Complex	Geometry	*E*_HOMO_	*E*_LUMO_	Δ*E*	η	ω	Dipole moment
HL		−6.288	−3.021	3.267	1.634	6.629	6.35
[CoL_2_]	SP	−5.862	−3.412	2.450	1.225	8.776	2.45
[NiL_2_]	SP	−5.743	−3.435	2.308	1.154	9.124	2.39
[CuL_2_]	SP	−5.867	−3.418	2.449	1.224	8.804	2.67
[ZnL_2_]	SDT	−5.991	−3.504	2.487	1.244	9.059	2.99

*Note*: Units are in eV, except for dipole moment which is in Debye.

The overall reactivity ranking of the modeled compounds, as indicated by their respective HOMO–LUMO energy gap (Δ*E*) and hardness (*η*), is [NiL_2_] > [CuL_2_] ≈ [CoL_2_] > [ZnL_2_] > HL. This suggests that the nickel complex is the most chemically reactive, with the reactivity of the cobalt and copper complexes being similar. The study results indicate that the complexes are expected to exhibit greater biological viability than the ligand, with the nickel complex anticipated to demonstrate higher activity compared to the other complexes. This finding aligns well with the experimental results from the antimicrobial and antioxidant assays, where the complexes outperformed the ligand in both tests. Specifically, among the complexes, NiL_2_ emerged as the most active, as predicted by the reactivity study. The heightened activity is attributed to the varying degrees of involvement of the complexes' HOMO and LUMO in interactions with biomolecules. The HOMO of the nickel complex is likely more engaged than its LUMO, while the zinc complex's LUMO is expected to participate more than its HOMO, as indicated by the *E*_HOMO_ and *E*_LUMO_ ratings. In the cases of the cobalt and copper complexes, an almost equal probability of utilizing HOMO and LUMO for interactions is observed. However, the copper complex, being more involved, exhibited greater biological viability compared to the cobalt complex, as evidenced in the biological study results.

The complexes are generally more electrophilic and electron‐deficient than the ligand, as indicated by the trend of the electrophilicity index (*ω*). This suggests that the complexes are more receptive to interacting with nucleophilic biomolecules. Despite the dipole moment data portraying the ligand as a more polar molecule than the complexes, the data equally indicate that the complexes possess sufficient polarity to facilitate significant van der Waals interactions in biological systems. The relatively smaller net dipole moments of the complexes suggest the presence of dipoles of similar strengths at opposite ends of the central metal ions within the complexes.

### 6.3 Analysis of Spin Density Distribution

One of the merits of spin density distribution (SDD) is its reflection of the magnetic properties of metal complexes. Paramagnetic complexes have one or more unpaired valence electrons hence, they possess singly occupied molecular orbital (SOMOs). Unlike electron density, SDD reveals the 3D shape of SOMOs in metal complexes. Of the four metallic complexes investigated in this work, only the cobalt and copper complexes possess paramagnetic centers due to the presence of a single unpaired electron in their respective metal d‐orbital. These complexes can thus be classified as open‐shell systems. The nickel and zinc complexes on the other hand are closed‐shell complexes since they have zero unpaired electrons and consequently lack a paramagnetic center.

**FIGURE 7:**
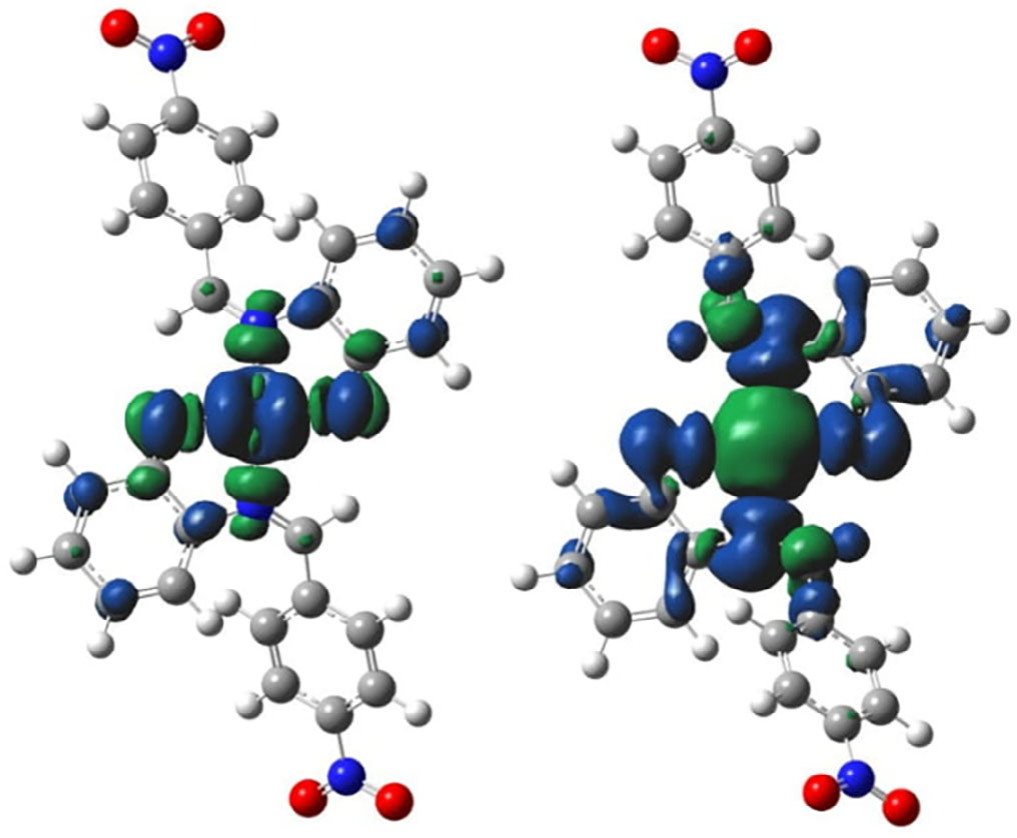
Isosurfaces of spin density distribution for [CoL_2_] (left) and [CuL_2_] (right) obtained at a cutoff of 0.02.

Another desirable usefulness of SDD is in the prediction of strength of covalent bond formed between a central metal ion and a ligand in a metal complex [77]. An unpaired electron at a paramagnetic center is conventionally assigned to a positive spin [78]. This positive spin can induce an opposite spin density at the surrounding atom through spin delocalization or spin polarization mechanism. Details of these mechanisms can be found elsewhere [78]. If *n* is the total spin density associated with the SOMOs of a metal complex, the larger fraction of *n* will be apportioned to the metal d‐orbital (being the major contributors to the SOMOs) while the remaining smaller fraction of the spin density will be delocalized toward the coordinated ligand atoms. Alternatively, the spin density may be distributed by spin polarization mechanism such that the positive part is drawn close to the atom that bears the unpaired electron (the metal atom) while the negative part is concentrated around the atom bonded to it (the ligand). This effect resonates through the molecule from the paramagnetic center, creating spin densities of opposing signs at moderately long distances from the centers (Figure 7). Consequently, the total spin density at the metal center is usually less than the number of unpaired valence electrons in their atomic orbital. This difference can reveal the degree of covalent behavior of a metal–ligand bond. The more the spread of spin density toward a ligand atom, the stronger the covalent character of the bond between the ligand and the metal, and the shorter the bond [78]. Comparison of the spin density isosurfaces of [CoL_2_] and [CuL_2_] (Figure 7) therefore shows that the bond between cobalt and the ligand is of a higher covalent character than that formed between copper and the ligand, which is in good accord with the trend of metal–ligand bond length seen previously in the infrared spectral study.

### 6.4 Topological Analysis

**FIGURE 8:**
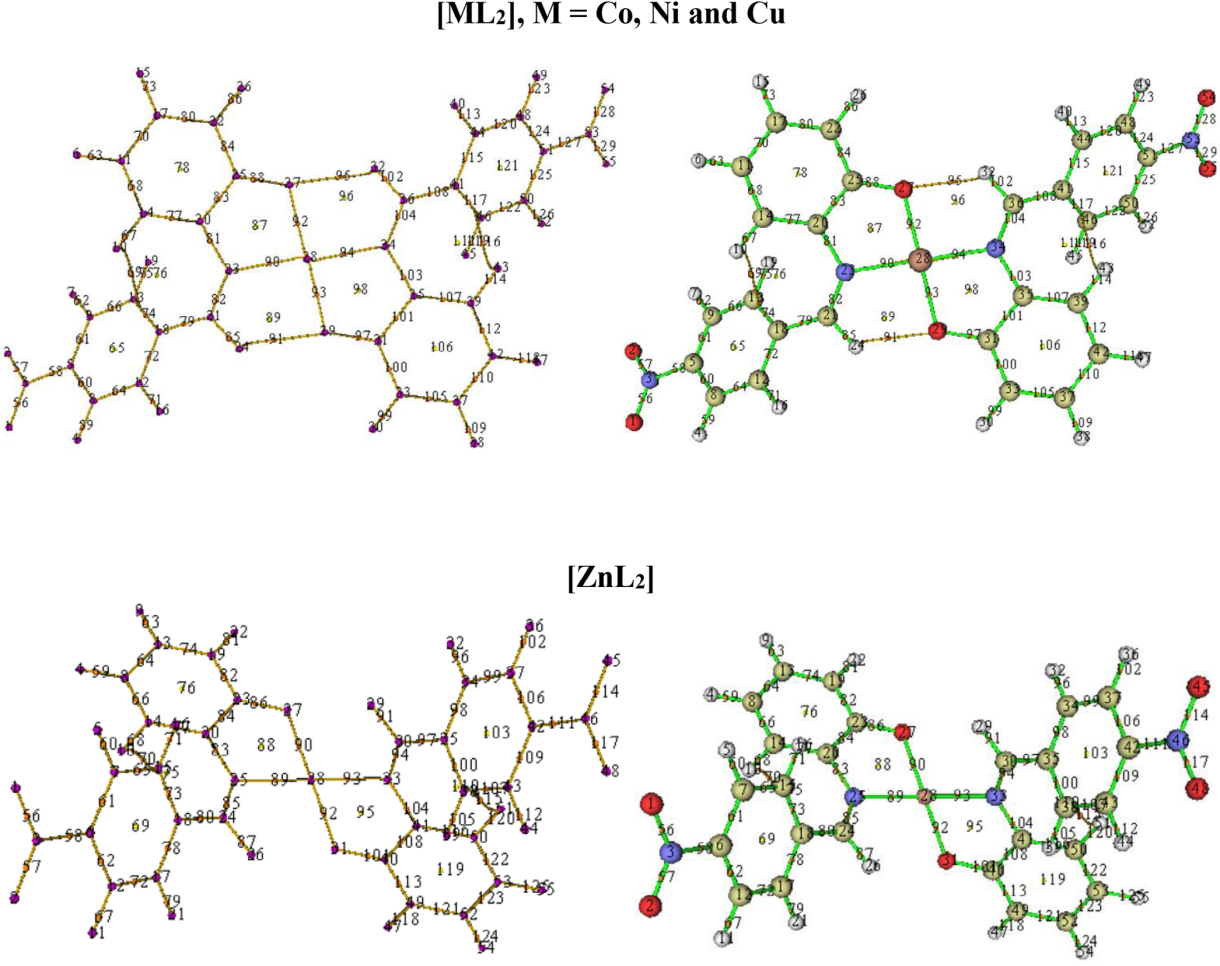
Images of the topologically analyzed complexes showing key critical points in the complexes.

The geometrically optimized complexes were topologically analyzed for important critical points (CPs) using Multiwfn 3.8.0 [79]. The key CPs located in the complexes are CPs (3, −3), (3, −1), and (3, +1) which are colored purple, orange and yellow, respectively (Figure 8).

Points 23, 27, 28, 29, and 34 are examples of CP of the type (3, −3) which indicate atomic positions in the complexes. Points 27 and 29 are the two coordinated oxygen atoms while 23 and 34 are the two iminic nitrogen atoms in the square planar complexes. Similar points in the zinc complex are 27 and 31, and 25 and 33. Point 28 indicates the central metal ions in all the complexes.

On the contrary, points 90, 92, 93, and 94 in the square planar complexes, and 89, 90, 92, and 93 in the zinc complex, are indicative of a (3, −1) type CP which describes inter‐nuclear regions, that is, bond formation between pairs of neighboring atoms. Points 92 and 93 (90 and 92 in the case of zinc complex) mark the existence of bonds between the central metal ion and the two coordinated oxygen atoms. Similarly, points 90 and 94 (89 and 93 in the case of zinc complex) reveal the two metal‐nitrogen bonds in the complexes.

(3, +1) type CP is a ring critical point (RCP) indicating formation of rings in the complexes. Notable examples are points 87, 89, 96, and 98 in the square planar complexes, and 88 and 95 in the zinc complex. The presence of RCPs 89 and 96 in the former is as a result of formation of two intramolecular H‐bonds involving the two coordinated O‐atoms. These weak bonds, marked as 91 and 95, show that the two hydrogen atoms (Points 24 and 32) are in‐plane with the region of coordination. This confirms a square planar geometry for [CoL_2_], [NiL_2_], and [CuL_2_]. On the other hand, similar RCPs are lacking in [ZnL_2_] due to the absence of a (3, −1) type CP between Points 27 and 29, and 26 and 31, which suggests that Points 26 and 29 are not in‐plane with 31 and 27, respectively. This confirms a tetrahedral geometry for [ZnL_2_].

Two additional (3, −1) type CPs located as Points 16 and 69 in the square planar complexes, and 15 and 70 in the zinc complex mark the formation of two additional H‐bonds believed to have been induced by the electron‐withdrawing effect of the two NO_2_ substituents on Points 13 and 46, and, 15 and 38, in the square planar and tetrahedral complexes, respectively.

### 6.5 NBO Analysis of Metal–Ligand Interaction

The strength of donor–acceptor interaction in the metal complexes was evaluated based on stabilization energy, E(2) derived by second order perturbation theory analysis of NBO Fock Matrix using to the relation:

(8) $$E\left(2\right)=q_{j}\frac{F{\left(i,j\right)}^{2}}{\varepsilon_{j}-\varepsilon_{i}}$$

where F(i,j) is the NBO Fock off‐diagonal element, $q_{j}$ is the occupancy of donor orbital, $\varepsilon_{j}$ and $\varepsilon_{i}$ are energies of the donor and acceptor NBO, respectively, and are the diagonal elements of the matrix [80].

The most important E(2)values for the ligand's interaction with the four metal ions through its imine nitrogen and deprotonated oxygen sites are compared in Table 9. This parameter serves as a means of quantifying the strength of orbital interaction between a donor and an acceptor. The larger the E(2)value, the more the favorability of charge transfer from the ligand (donor) to the metal ion (acceptor), and the stronger the interaction. Thus, the E(2)values in the table indicate that the interaction via oxygen is generally stronger than the one through nitrogen. Also, strength of interaction ranks among the complexes as [NiL_2_] > [ZnL_2_] > [CoL_2_] > [CuL_2_], which suggests that nickel ion formed the strongest M─O and M─N bonds with the ligand while copper ion formed the weakest metal–ligand bonds.

**TABLE 9:** Stabilization energies of the complexes as obtained from second order perturbation theory analysis of NBO Fock Matrix.

Complexes	Donor NBO (*i*)	Acceptor NBO (*j*)	*E_j_* − *E_i_* (eV)	*F_i,j_* (eV)	*E*(2) (eV)
[CoL_2_]	LP(N)	LP*(Co)	5.44	2.72	1.05
	LP(O)	LP*(Co)	7.08	3.35	1.27
[NiL_2_]	LP(N)	LP*(Ni)	5.44	2.61	2.00
	LP(O)	LP*(Ni)	6.26	3.08	2.38
[CuL_2_]	LP(N)	LP*(Cu)	4.62	2.01	0.69
	LP(O)	LP*(Cu)	4.62	2.50	1.07
[ZnL_2_]	LP(N)	LP*(Zn)	11.16	2.88	1.42
	LP(O)	LP*(Zn)	12.25	3.48	1.83

### 6.6 Thermodynamics of Complexation

The changes in internal energy (Δ*U*), enthalpy (Δ*H*), entropy (Δ*S*), and Gibb's free energy (Δ*G*) of complexation at 298.15 K are given in Table 10 as obtained from DFT calculations. Δ*U* gives an insight on the stability of the complexes as a result of changes in kinetic, potential and electric energies of the ligand and metal ions upon complexation. Based on this consideration, the ligand and the metal ions are energetically lower (hence more stable) in complex form, and the most stable complex is [NiL_2_].

**TABLE 10:** DFT‐predicted thermodynamic parameters at 298.15 K.

Complex	Δ*U* (kJ/mol)	Δ*H* (kJ/mol)	Δ*S* (kJ/mol)	Δ*G* (kJ/mol)
[CoL_2_]	−2717.03	−2721.98	−0.350	−2619.43
[NiL_2_]	−2866.86	−2871.82	−0.355	−2768.69
[CuL_2_]	−2634.23	−2638.96	−0.331	−2540.14
[ZnL_2_]	−2475.02	−2479.98	−0.313	−2386.60

Δ*H* is a parameter that shows the magnitude and direction of heat flow during a process. A positive ΔH value is indicative of a bond‐breaking process in which energy is absorbed (endothermic) while a negative value signifies that energy is released (exothermic) during a process that involves formation of new bonds. Thus, the values of Δ*H* in Table 10 suggest that the formation of all the complexes will be exothermic, and the largest heat evolution will be obtained during the formation of the nickel complex. Δ*S* reveal the level of agitation/disorderliness in a system. Positive Δ*S* indicate a dissociative process that leads to increased disorderliness while negative Δ*S* is characteristic of a process that leads to an increase in orderliness. Hence, the Δ*S* values obtained indicate the formation of all the complexes as an associative process that is expected to occur with increased orderliness such that the highest orderliness is obtained with the nickel complex.

Finally, Δ*G* is a parameter that reveals the feasibility of a process from a thermodynamic viewpoint. In the present case, it reveals the spontaneity of complex formation from the various ligand–metal combinations. Spontaneous processes are characterized by negative Δ*G*s while nonspontaneous ones are identified with positive Δ*G* values. Also, the more negative the Δ*G* value, the higher the spontaneity, and vice versa. Therefore, the Δ*G* values obtained for all the complexes indicate that their formation is thermodynamically feasible, meaning that the complexation process is self‐driven. Overall, the order of stability and spontaneity is [NiL_2_] > [CoL_2_] > [CuL_2_] > [ZnL_2_].

### 6.7 Molecular Docking Study

**FIGURE 9:**
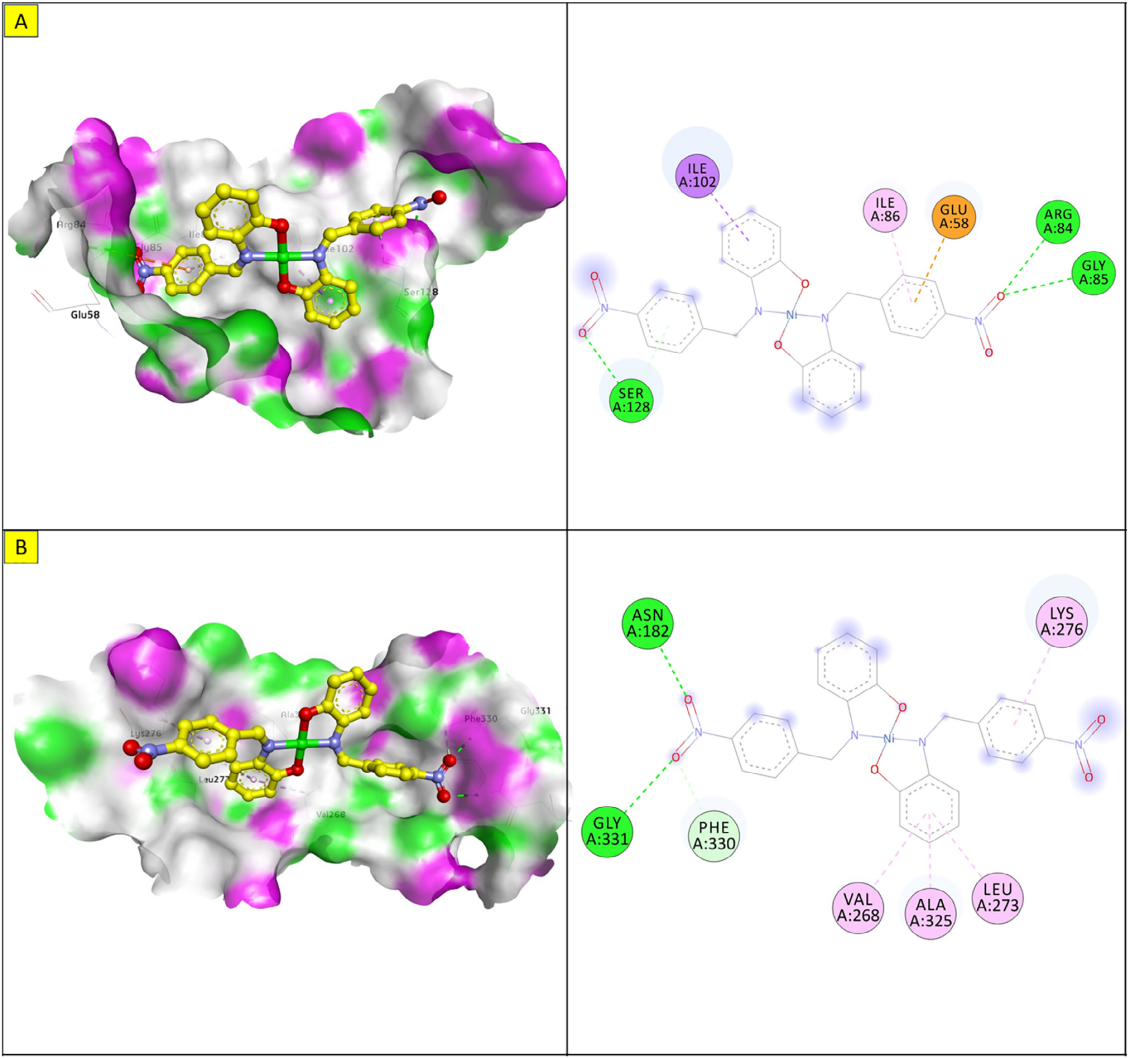
3D and 2D view of the lowest energy poses of NiL_2_ in the binding pocket of (a) *Staphylococcus aureus* topoisomerase. (b) *Bacillus subtilis* DNA gyrase.

**FIGURE 10:**
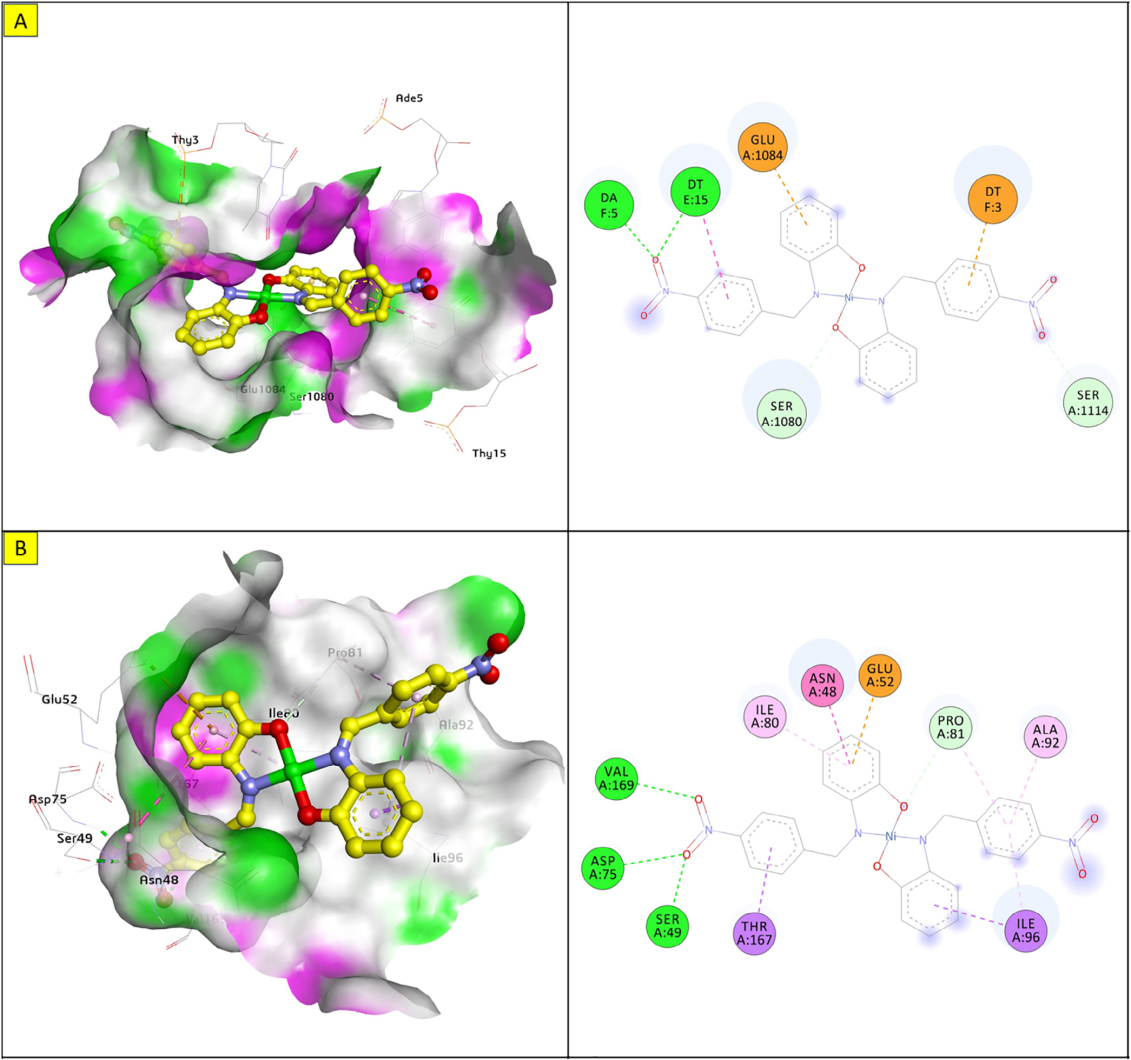
3D and 2D view and surface mapping for the best docked pose of NiL_2_ in the active sites of (a) *Klebsiella pneumonia* topoisomerase. (b) *Pseudomonas aeruginosa* DNA gyrase.

**FIGURE 11:**
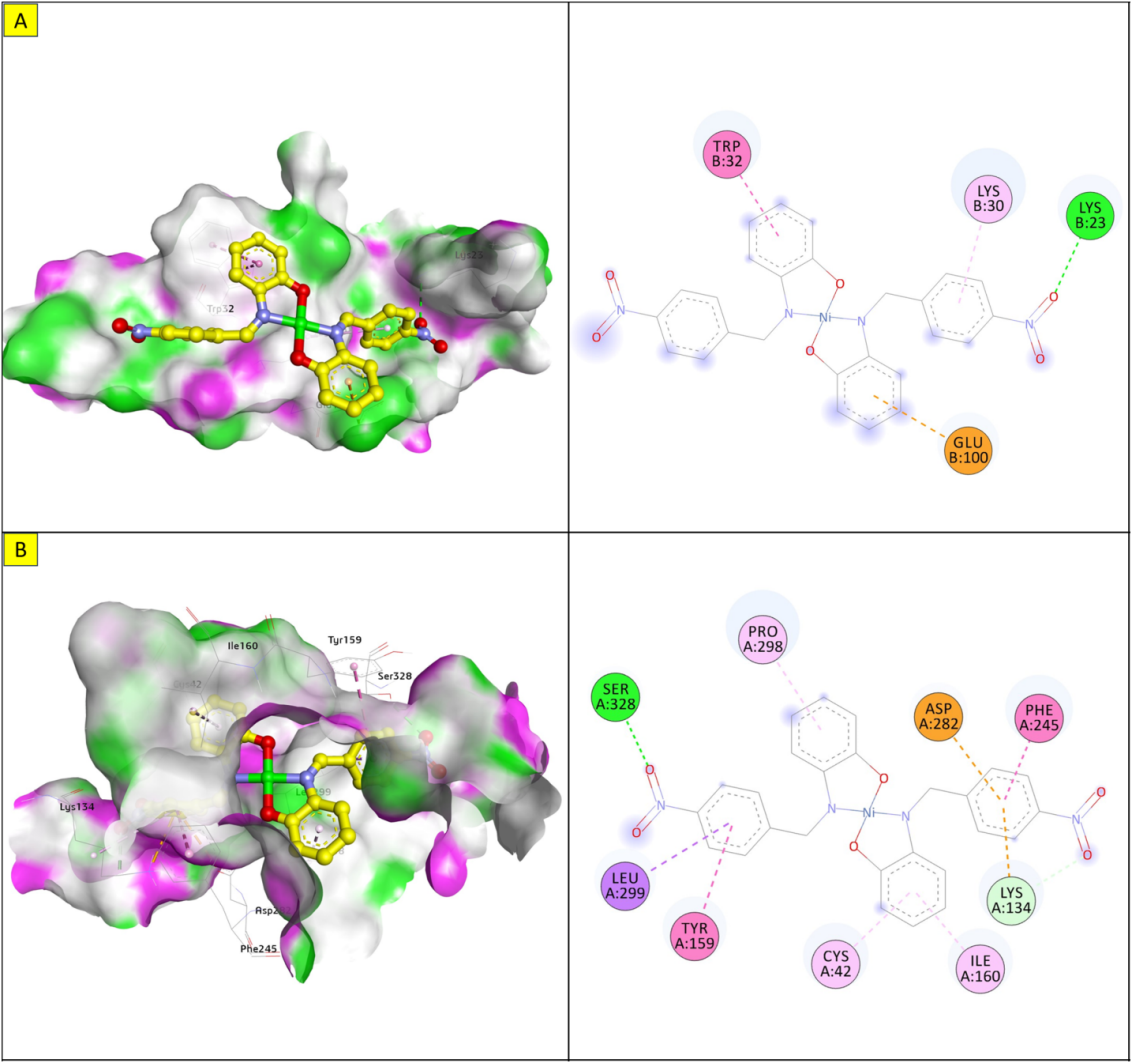
3D and 2D view and surface mapping of the best docked pose of NiL_2_ in the active sites of (a) SOD. (b) NADPH oxidase.

Molecular docking was performed to study the binding of the lead compound, NiL_2_, with the target proteins. The binding scores (kcal/mol) of the best‐docked poses of the ligands are presented in Table 11, and their interactions are presented in Figures 9, 10, 11.

**TABLE 11:** Binding energies (kcal/mol) of the best docked pose of the studied molecules.

Entry	Target			
	*Staphylococcus aureus*	*Bacillus subtilis*	*Klebsiella pneumonia*	*Pseudomonas aeruginosa*
	Binding energy (kcal/mol)			
NiL_2_	−8.2	−7.8	−8.8	−9.0
Ciprofloxacin	−6.8	−5.8	−8.8	−6.8

Entry	Target	
	SOD	NADPH
	Binding energy (kcal/mol)	
NiL_2_	−7.8	−11.1
Ascorbic acid	−5.4	−6.0

The docking energy of NiL_2_ with topoisomerase of *S. aureus* was −8.2 kcal/mol. NiL_2_ interacts with Arg84, Gly85, and Ser128 amino acids via conventional hydrogen bonds at a distance of 2.90, 2.36, and 2.04 Å. It also forms π–sigma contact with Ile102 residue beside π bond with the side chain of Ile86 amino acid. Moreover, NiL_2_ interacts with the anionic side chain of Glu58 residue via π bond Figure 9. From docking results, we found that NiL_2_efficiently binds to DNA gyrase of *B. subtilis* with binding score −7.8 kcal/mol. It forms two conventional hydrogen bonds with Asn182 and Gly331 residues at a distance 2.24 and 2.16 Å, respectively. Also, it interacts with the side chain of Val268, Leu273, Lys276, and Ala325 residues via π bonds in addition to C─H bond with Phe330 amino acid Figure 8. Furthermore, NiL_2_ exhibits good binding affinity −8.8 kcal/mol when interacting with topoisomerase of *K. pneumonioe*. NiL_2_ binds with Ade5 and Thy15 nucleotides of the DNA via two conventional hydrogen bonds. Also, it forms π–anion bonds Glu1084 residue and Thy3 nucleotide in addition to π–π bond with Thy15 of DNA. In addition to these interactions, NiL_2_ also interacts with Ser1080 and Ser1114 residue via C─H bonds (Figure 10). NiL_2_ also fits well with the DNA Gyrase of *P. aeruginosa* and forms strong conventional hydrogen bonds with Ser49, Asp75, and Val169 amino acids at a distance 3.29, 2.52, and 2.54 Å, respectively. Also, it forms π bond with the anionic side chain of Glu52 residue in addition to π–sigma bonds with Ile96 and Thr167 amino acids. NiL_2_ interacts with the side chain of Ile80, Pro81, Ala92, and Ile96 amino acids via π bonds. In addition, it forms π bond with the amide group of Asn48 residue in addition to C─H bond with Pro81 amino acid (Figure 10). As shown in Figure 11, NiL_2_ binds with SOD via formation of conventional hydrogen bond with Lys23 amino acid at a distance 2.90 Å. Also, it forms π bond with the anionic side chain of Glu100 residue beside π–π bond with Trp32 amino acid. Moreover, it forms a π bond with the side chain of Lys30 amino acid. The docking energy of the binding of NiL_2_ with NADPH was −11.1 kcal/mol. We found that NiL_2_ binds with the active site of the protein via conventional hydrogen bond with Ser328 residue at a distance 2.64 Å in addition to C─H bond with Lys134 amino acid. Furthermore, it forms π bonds with the cationic and anionic side chains of Lys134 and Asp282 amino acids, respectively. NiL_2_ also interacts with Leu299 amino acid via π–sigma bond beside π–π bonds with Tyr159 and Phe245 residues (Figure 11). Furthermore, it interacts with the side chain of Cys42, Ile160, and Pro298 amino acids via π bonds. Overall, the result corroborated well with experimental findings.

## 7 Conclusion

Four transition metal complexes of Co(II), Ni(II), Cu(II), and Zn(II) derived from a nitro‐substituted Schiff base ligand (HL) were synthesized and characterized through various analytical and spectroscopic techniques. The results of the characterization techniques reveal that the ligand acted as a bidentate ligand, coordinating to the metal ion via the oxygen and nitrogen atoms of the phenolate and imine moieties, respectively. This coordination led to a tetrahedral geometry for the Zn(II) complex, square planar geometries for the Co(II) and Ni(II) complexes, and a distorted square planar geometry for the Cu(II) complex. The ligand and its complexes were evaluated for antimicrobial and antioxidant activity using disc diffusion, broth dilution, and DPPH assay methods. The results of these studies demonstrate that all the complexes exhibited higher activity than the ligand in both assays, revealing a concentration‐dependent activity where the NiL_2_ complex displayed the most significant activity in each case. To delve into the electronic and molecular characteristics of the compounds, as well as the interaction mechanisms of the lead compound with the target receptor, and to correlate these findings with experimental studies, a combined density functional theory (DFT) and molecular docking investigation was carried out. The theoretical results align with experimental observations, providing valuable information on the electronic properties, stability, and mechanism of action of compounds.

## Author Contributions

This study was a collaborative effort among all the authors, who contributed to its conception and design. The data acquisition, analysis, and interpretation were carried out collectively. The manuscript was carefully drafted and underwent critical revisions by all the authors to ensure the incorporation of substantial intellectual content. The final approval for the publication of the manuscript was granted by all the authors, signifying their agreement and endorsement of the work.

## Conflicts of Interest

The authors declare no conflicts of interest.

## Supporting information




**Supporting File 1**: cbdv70157‐sup‐0001‐SuppMat.cif.

**Supporting File 2**: cbdv70157‐sup‐0002‐SuppMat.docx.
Crystallographic information CCDC  no: 2382972 contains the supplementary crystallographic data for the ligand (**HL**), and can be obtained free of charge from the Cambridge Crystallographic Data Centre via http://www.ccdc.cam.ac.uk/data_request/cif

## Data Availability

The data that support the findings of this study are available in the Supporting Information of this article.
