# Supplementary material for: Synthesis, Characterization, Biological Evaluation, DFT Calculations, and Molecular Docking Study of Transition Metal Complexes Derived From a Schiff Base Ligand
Source: Chem Biodivers. 2025 Jun 26;22(11):e00940. doi: 10.1002/cbdv.202500940 (PMC12629167; doi:10.1002/cbdv.202500940)
Supplement: Supplementary file 2 — Supporting File 2: cbdv70157‐sup‐0002‐SuppMat.docx. Crystallographic information CCDC no: 2382972 contains the supplementary crystallographic data for the ligand (HL), and can be obtained free of charge from the Cambridge Crystallographic Data Centre via http://www.ccdc.cam.ac.uk/data_request/cif [file CBDV-22-e00940-s002.docx]

***Supplementary information***

**Experimental**

**Synthesis of the ligand precusor, nitro benzaldehyde**

The synthesis of HL was initiated by nitration of benzaldehyde [1] using the following procedure: Sulphuric acid (2.5 mL, 46.95 mmol, 1 eq; 98%) was combined with nitric acid (2.2 mL, 46.95 mmol, 1 eq; 55%) in a 100 mL reaction flask placed on ice at 0 °C. The mixture was stirred, and then benzaldehyde (4.74 mL, 46.95 mmol, 1 eq) was added dropwise while maintaining the flask on ice. After the addition of benzaldehyde, the solution was refluxed at 65 °C for 1 hour. Subsequently, the hot solution was poured into a beaker containing crushed ice and stirred using a glass rod. The resulting precipitate was obtained by vacuum-filtering through a Buchner funnel and further purified by recrystalisation in ethanol, yielding the final product, 4-nitrobenzaldehyde (**Scheme S1**).Yield: 3.96 g (55.9 %); M.p. 42-45 °C; ^1^H NMR (500 MHz, DMSO-*d_6_*); δ_H_ (ppm) = 8.16 (d, 2H, *J* = 7.0 Hz, Ar), 8.41 (d, 2H, *J* = 7.5 Hz, Ar), 10.16 (s, 1H, CHO); ^13^C NMR (125 MHz, DMSO-*d_6_*); δ_C_ (ppm) = 124.2 (2C), 130.6 (2C), 140.0, 150.6, (ArC); 192.2 (CHO); IR_ATR_: *v_max_*/cm^-1^: *v_(CHO)_*= 2839, *v_(C=N)_*= 1697, *v_(NO)_*= 1535, *v_(NO)_*= 1334, *v_(C-N)_*= 1185 cm^-1^; CHN Anal. Calculated for C_7_H_5_NO_3_: C, 55.63; H, 3.33; N, 9.27; found: C, 55.61; H, 3.32; N, 9.26.

**Scheme S1**: Synthesis of nitro benzaldehyde: i = H_2_SO_4_ (98 %)/HNO_3_ (55 %); ii = 0 ˚C /65 °C; 1 h.

**Single-crystal X-ray Diffraction Analysis**

Attempts to obtain single crystals suitable for data collection for all the compounds, except for the ligand, HL, were unsuccessful. However, the ligand, HL, was successfully obtained in methanol through a slow evaporation process that took 72 hours. The crystallographic data of the ligand, HL, was collected at 293 K using an APEXII instrument with Mo Kα (λ = 0.71073) radiation. The collected frames were processed using Bruker SAINT for integration [[3](#_ENREF_43)]. Subsequently, absorption effects were reduced using SADABS [[4](#_ENREF_44)], and the structures were solved using SHELXT [[5](#_ENREF_45)]. Refinement of the structures was performed using SHELXL [[6](#_ENREF_46)]. Non-hydrogen atoms were refined anisotropically using the least squares method, while hydrogen atoms were placed geometrically and refined using a riding approximation with isotropic displacement parameters 1.2 times (C-H) or 1.5 times (O-H) the Ueq of the parent atom [[7](#_ENREF_47)]. The crystal structure graphics were generated using Mercury software [[8](#_ENREF_48)].Crystal Data and details of the refinement are given in Table S1

**2.4. Biological Evaluation**

**2.4.1. Stability Study in Aqueous Buffer**

The stability analysis of the metal complexes and the free ligand was conducted in an aqueous/DMSO mixture. The compounds were dissolved in a solution of 5% DMSO–KH_2_PO_4_ (50 mM, pH 7.5), and their spectra were monitored over time, with spectral acquisition ranging from 250 to 800 nm at seven-day intervals.

**Stoichiometric and Stability Constant Determination**

The stoichiometric ratio of M(II) to the ligand in the complexes was determined using Job’s continuous variation method, following the techniques reported in the literature [[9](#_ENREF_49)]. In this method, different volumes (0, 1, 2, 3, 4, 5, 6 cm³) of 0.01 M M(II) solutions were successively pipette into seven 50 cm³ volumetric flasks. Corresponding aliquots (6, 5, 4, 3, 2, 1, and 0 cm³) of the 0.01 M ligand were added, maintaining a constant mole fraction in the solution. The absorbance of each solution was measured at the wavelength of maximum absorbance of the complex, initially determined by scanning wavelengths from 400 nm to 800 nm. This process was repeated for each mole fraction of the complex at different temperature ranges (30-60 °C), with absorbance recorded immediately after mixing. The data obtained were used to determine the stoichiometric ratio and stability constant of the complexes.

**PXRD and SEM-EDX Studies**

Several attempts made to isolate suitable crystals of the complexes for single crystal X-ray diffraction (SCXRD) data collection were futile. Therefore, further structural elucidation and chemical composition analysis was achieved using powder X-ray diffraction (PXRD), scanning electron microscopy (SEM), and energy dispersive X-ray (EDX) spectroscopies. The diffractograms and spectra are presented in (Figs. S17-19, S26-28, S32-34, and S41-43), respectively. The XRD diffraction patterns for the complexes were obtained in the 2Θ = 0-90 ^o^ range, and the diffraction patterns exhibit several reflections with peaks that are clearly defined and sharply crystalline, ranging from 0 to 60 ^o^, and proving that the compounds are crystalline [10]. The average crystallite sizes (d_XRD_) of the complexes were determined using Scherers' formula [11] as 79, 82, 74, and 72 nm, for CoL_2_, NiL_2_, CuL_2_, and ZnL_2_, respectively. This indicates that ZnL_2_ has the highest crystallinity [12].

The particle morphology and chemical composition of the ligand were investigated using SEM and EDX analyses and compared to the complexes. Figs. S18-19, 27-28, 33-34, and 42-43 show SEM images and EDX spectra of ligand and its complexes. The ligand's particle morphology shows well-arranged block-like structures in various forms different from the complexes whose morphologies are characterized by a fine rod-like array of various sizes and shapes, indicating the complexes' crystalline nature. This difference in particle morphology between the ligand and the complexes is indicative of the formation of new materials (i.e. the complexes). The chemical compositions of the complexes were further examined using EDX, and the obtained spectra show peaks which quantitatively account for the elements present in the complexes in line with their proposed structures.

**Spectroscopic Data of ligand and its complexes**


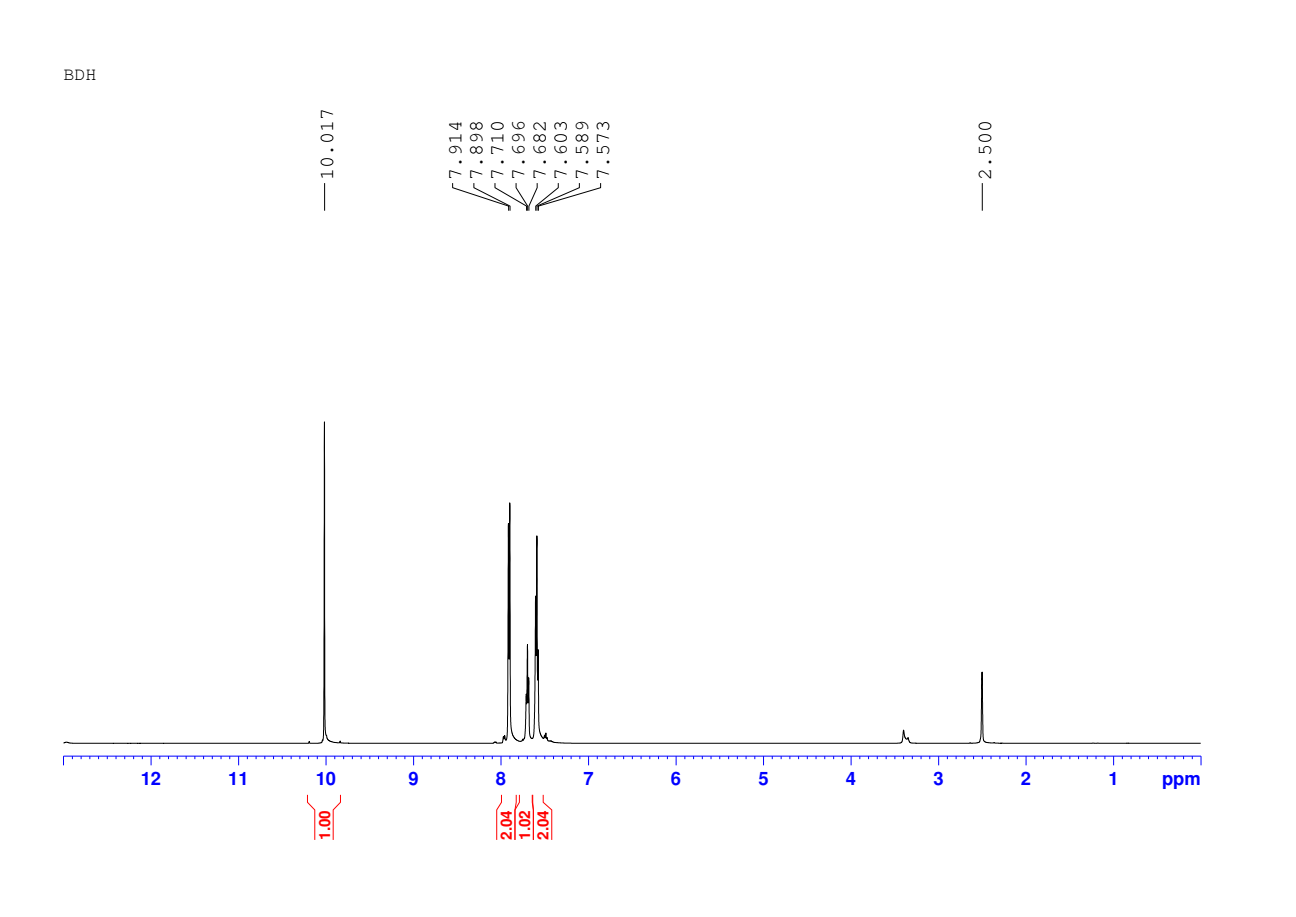


**DMSO-*d_6_***

**d**

**c**

**b**

**a**

**Figure S1:** ^1^H NMR spectrum of benzaldehyde recorded at 298 K using (500 MHz, DMSO-*d_6_*).


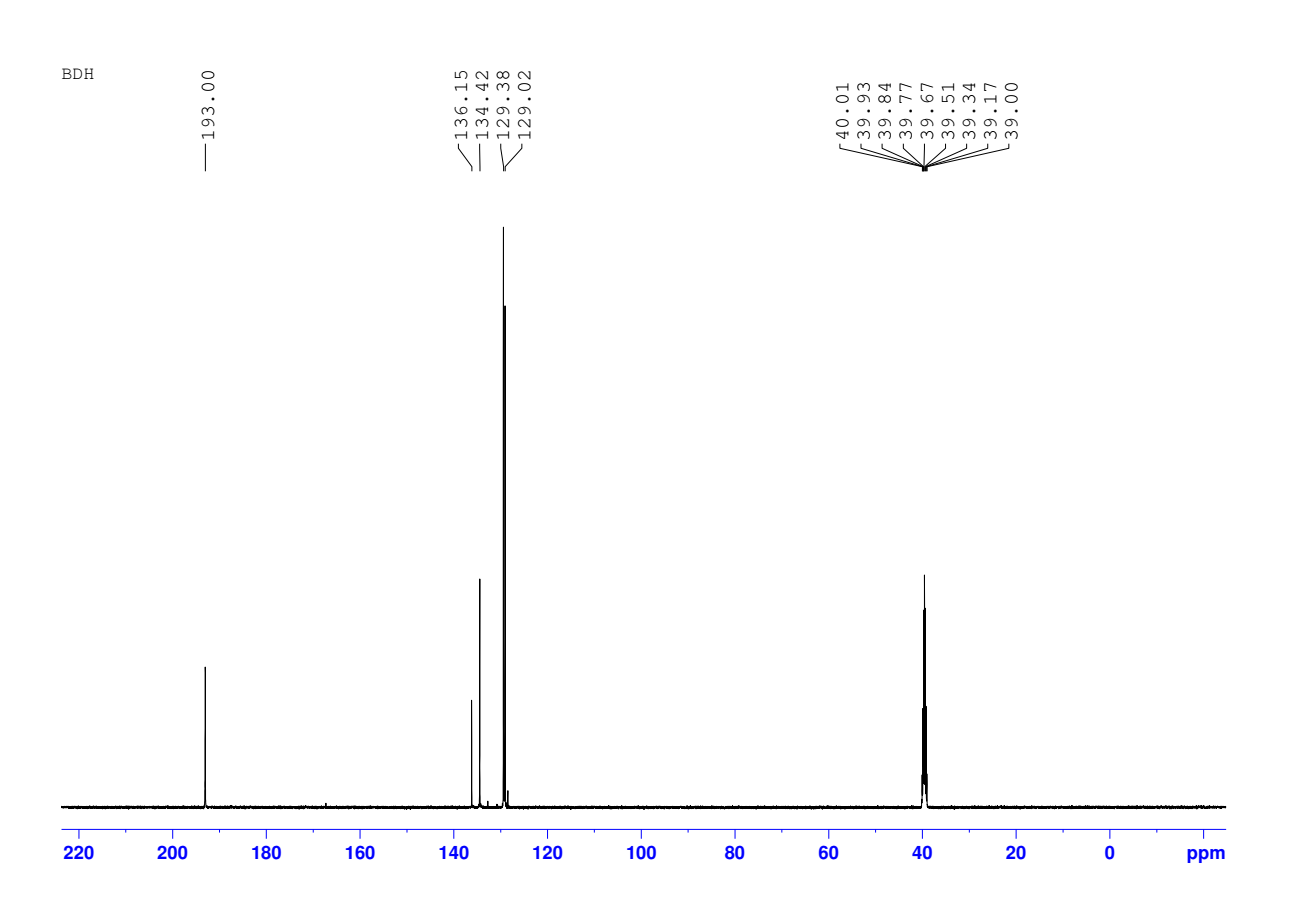


**d,e**

**c**

**b**

**a**

**Figure S2:** ^13^C NMR spectrum of benzaldehyde recorded at 273 K using (125 MHz, DMSO-*d_6_*).


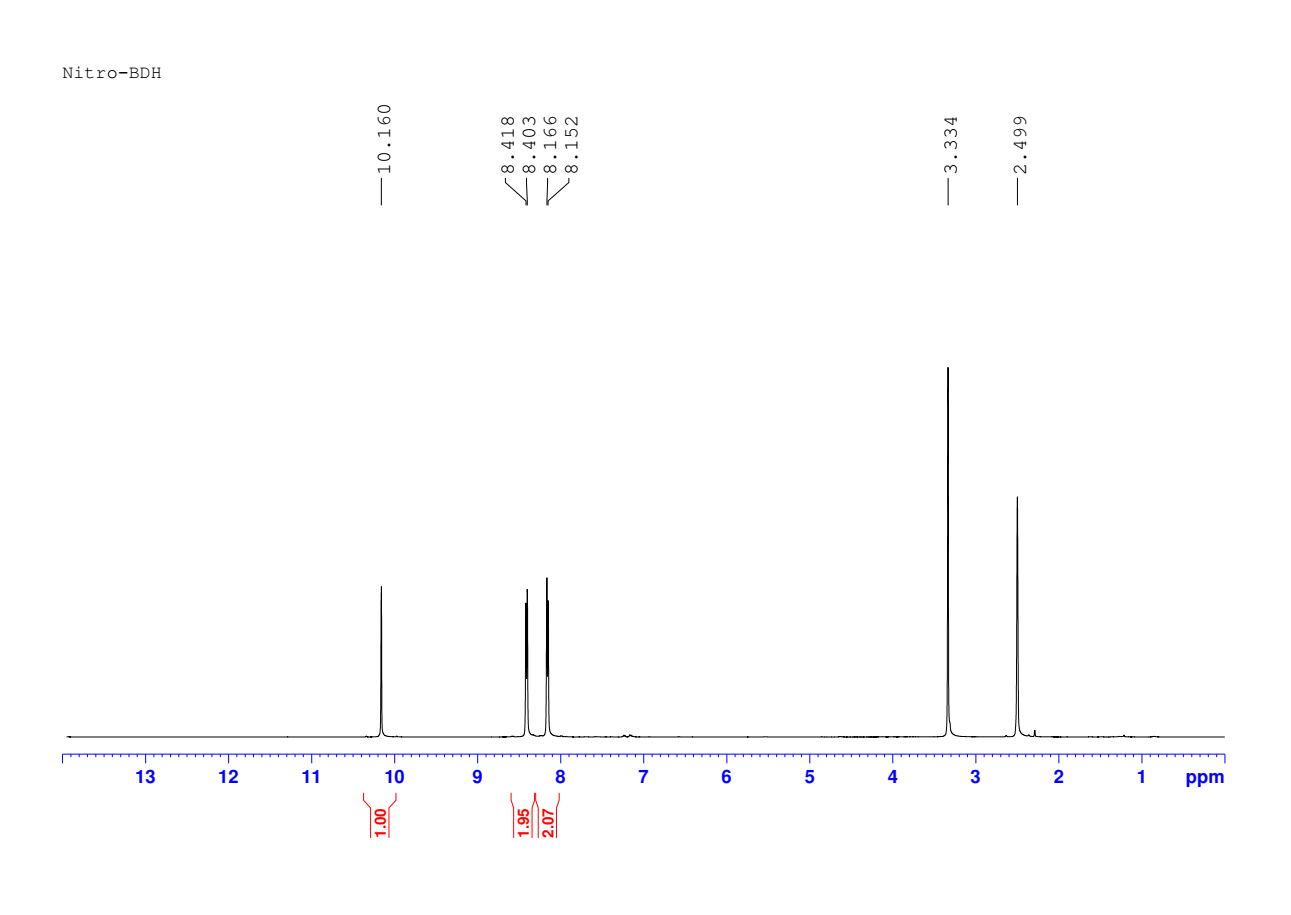


**b**

**c**

**a**

**Figure S3:** ^1^H NMR spectrum of 4-nitrobenzaldehyde recorded at 273 K using (500 MHz, DMSO-*d_6_*).


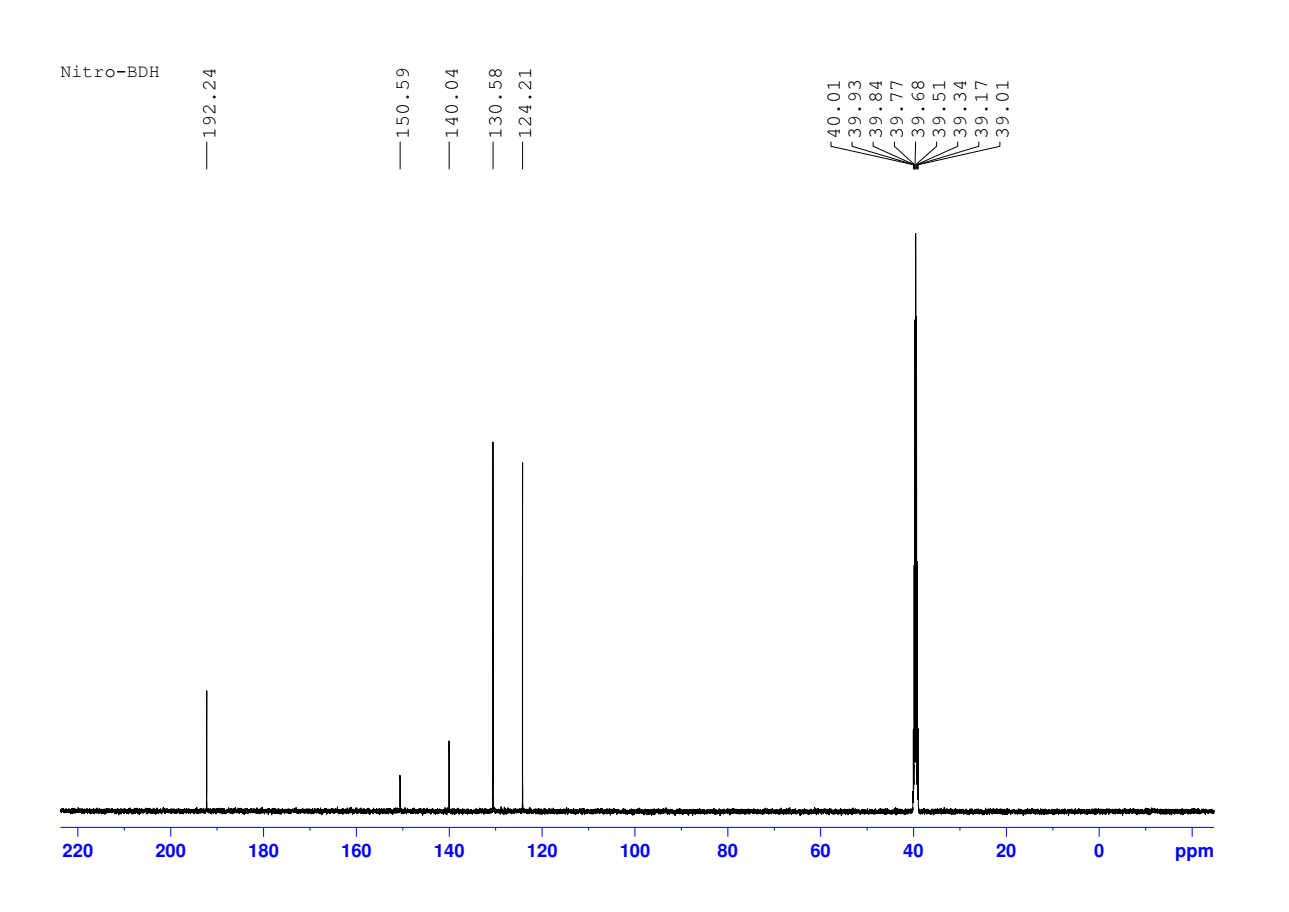


**d**

**c**

**b**

**e**

**a**

**Figure S4:** ^13^C NMR spectrum of 4-nitrobenzaldehyde recorded at 273 K using (125 MHz, DMSO-*d_6_*).


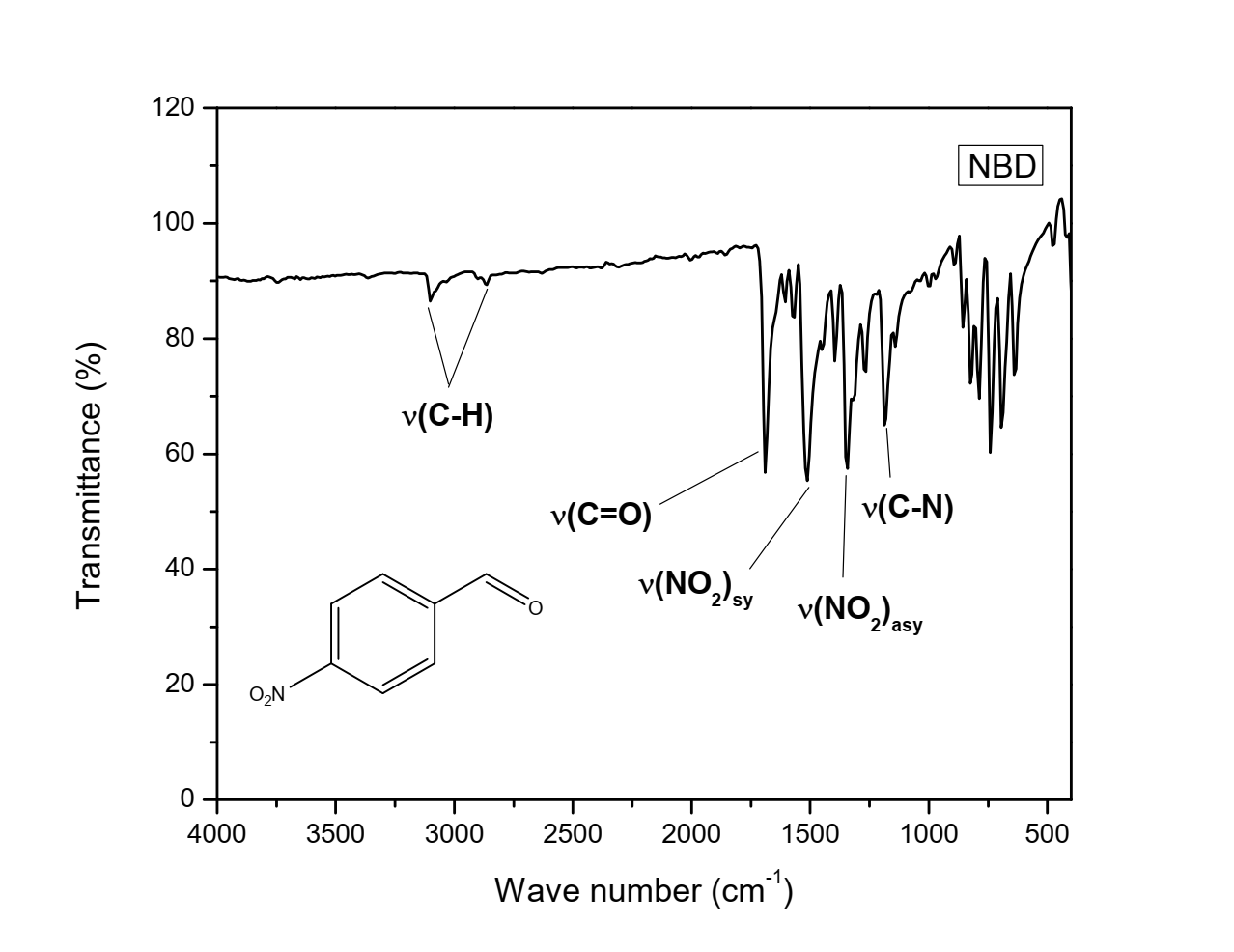


**Figure S5:** FTIR spectrum of 4-nitrobenzaldehyde recorded in solid state using ATR method.


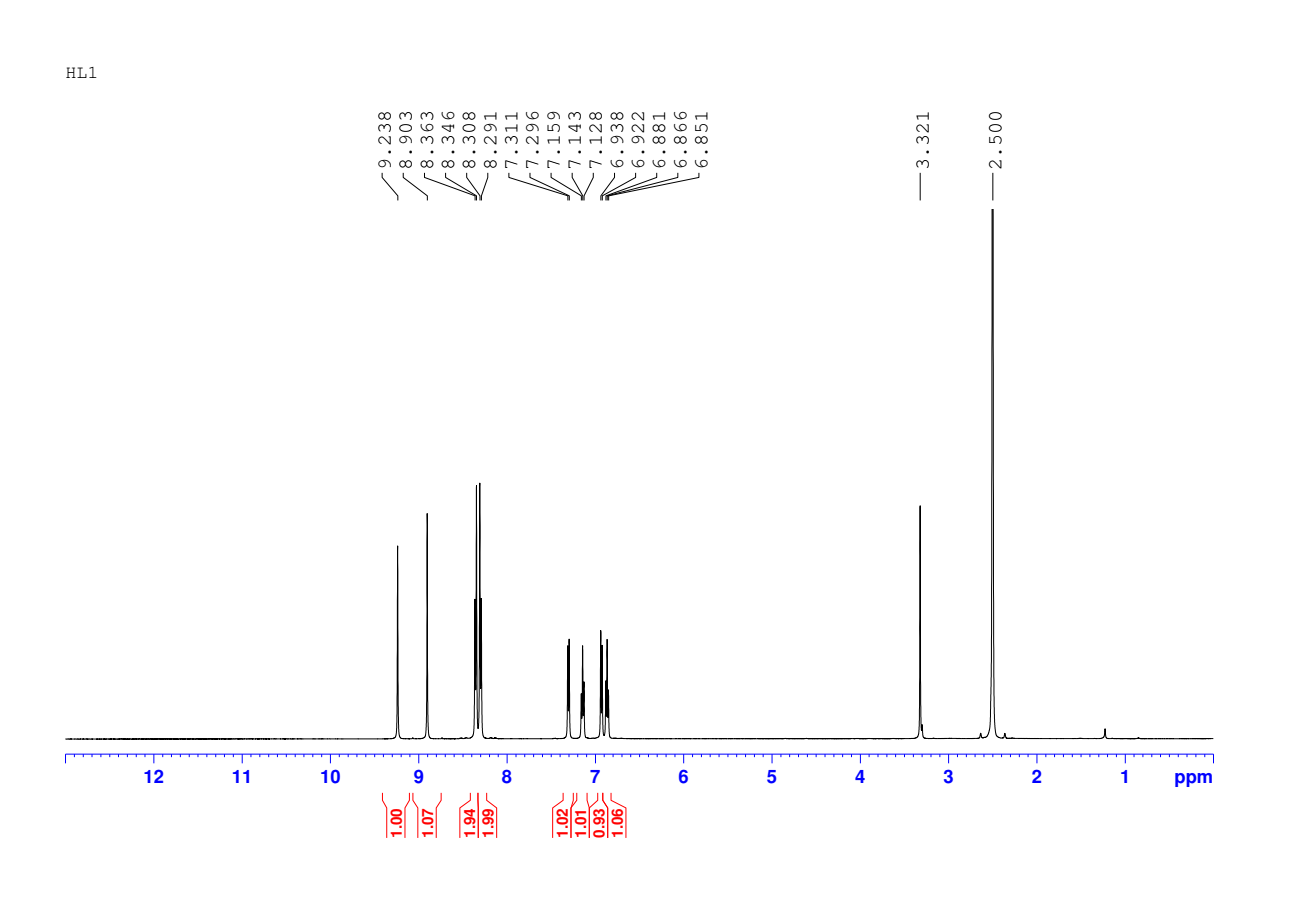


**e,f, g,h**

**d**

**c**

**a**

**b**

**Figure S6:** ^1^H NMR spectrum of the Schiff base ligand, **HL** recorded at 273 K using (500 MHz, DMSO-*d_6_*).


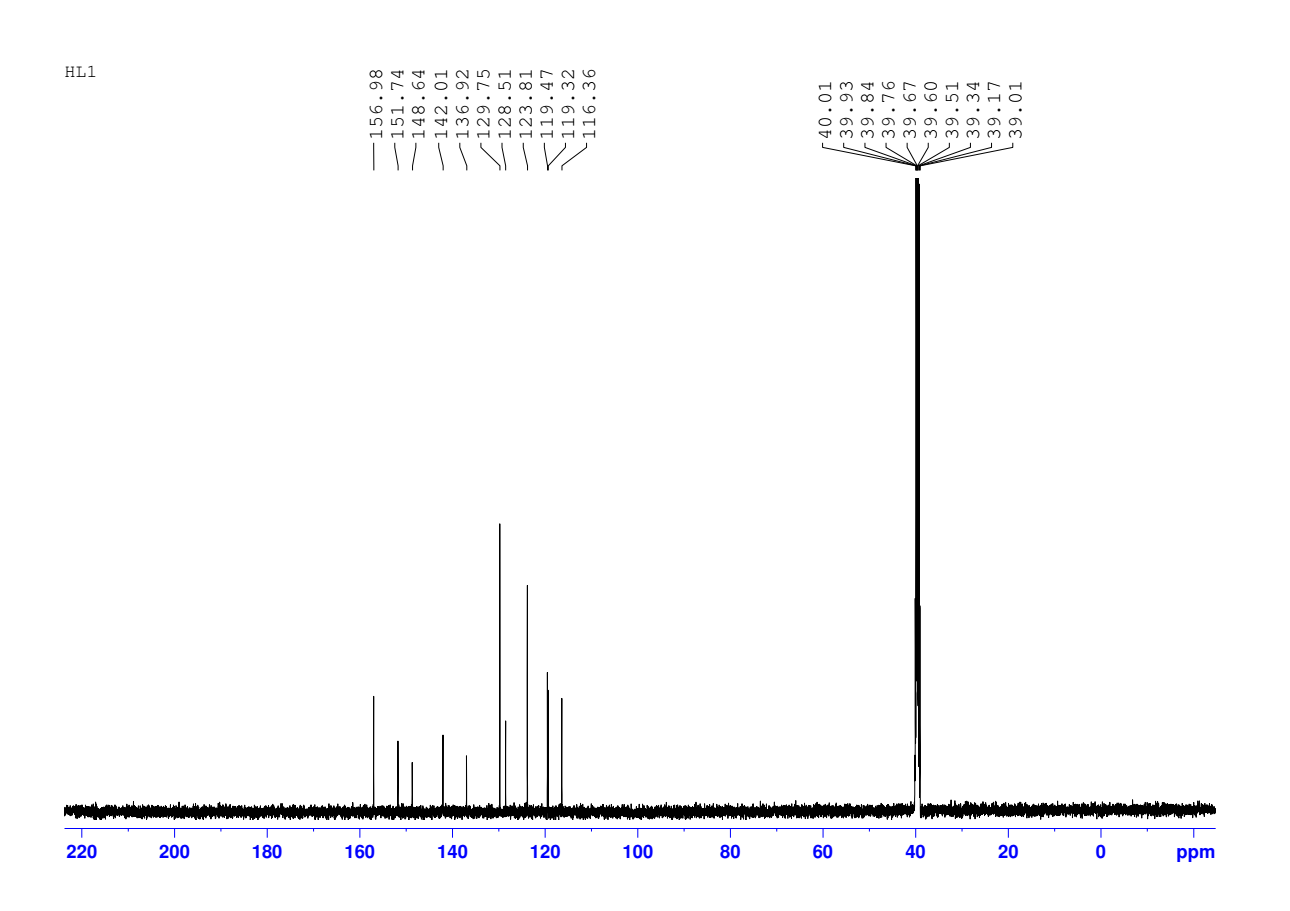


**f,g,h,i,j**

**b,c,d, e**

**a**

**Figure S7:** ^13^C NMR spectrum of the Schiff base ligand, **HL** recorded at 273 K using (125 MHz, DMSO-*d_6_*).


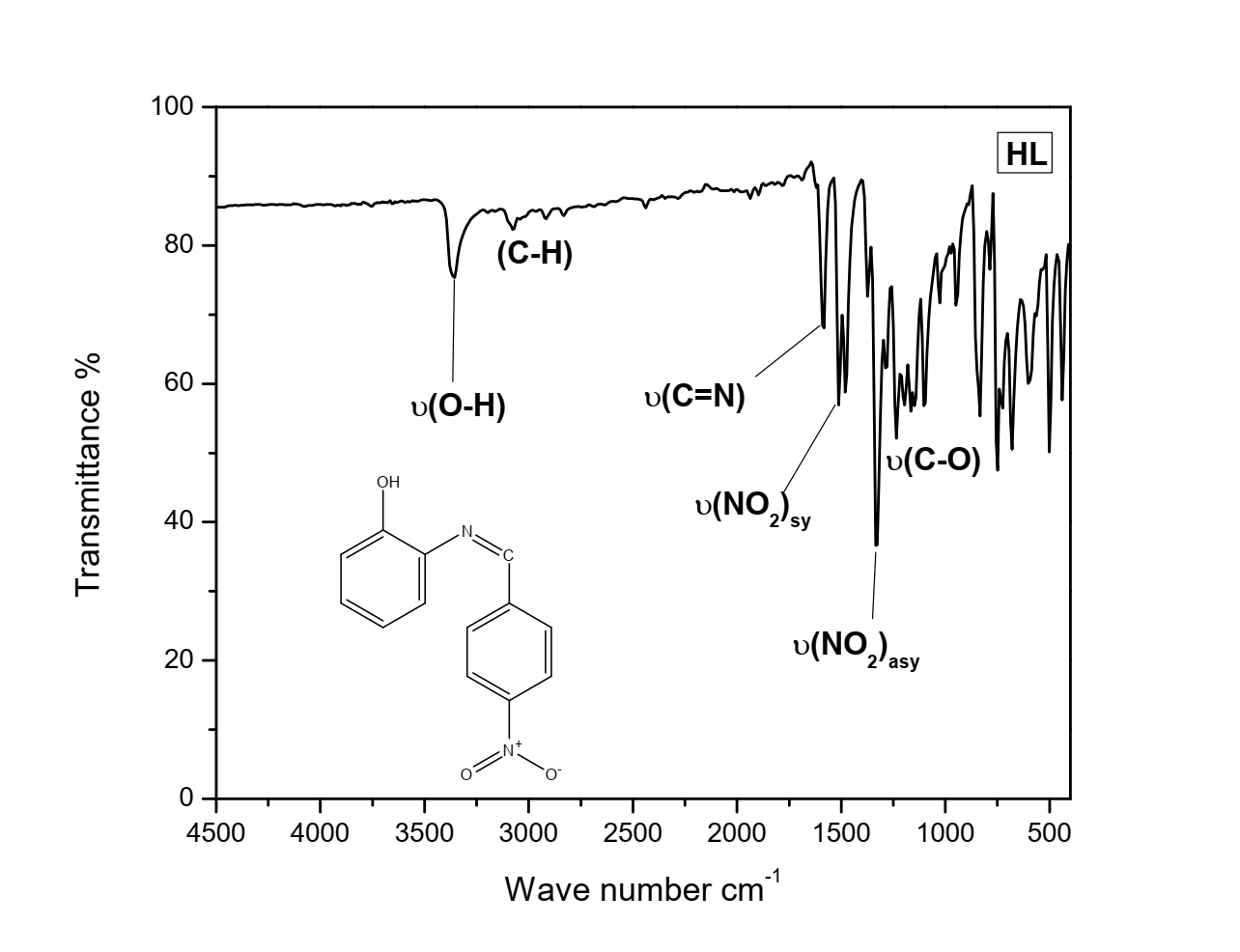


**Figure S8:** FTIR spectrum of the Schiff base ligand, **HL** recorded in solid state using ATR method


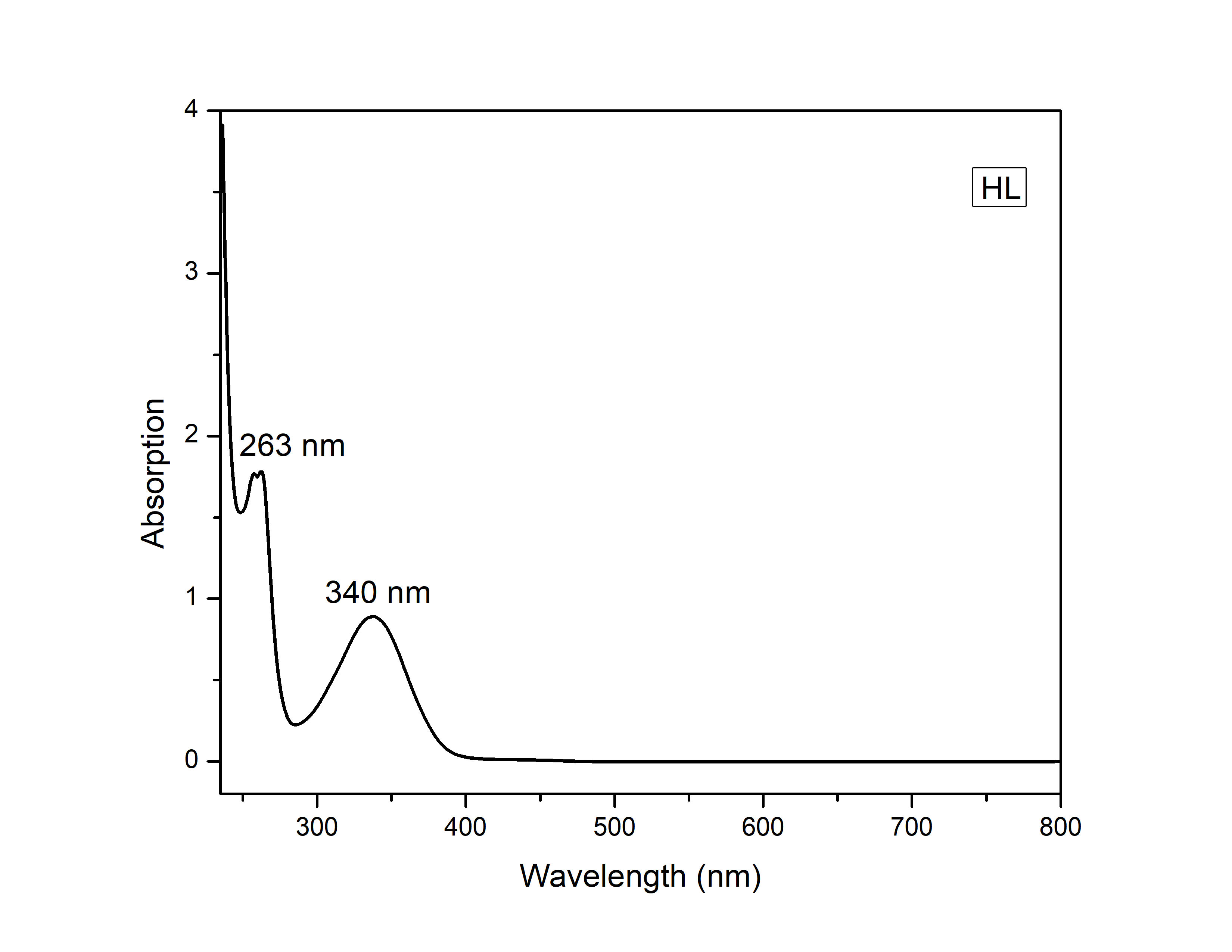


**n→π^*^**

**π→π^*^**

**Figure S9:** UV-Vis spectrum of the Schiff base ligand, **HL** recorded in DMSO (10^-3^ M) at 273 K


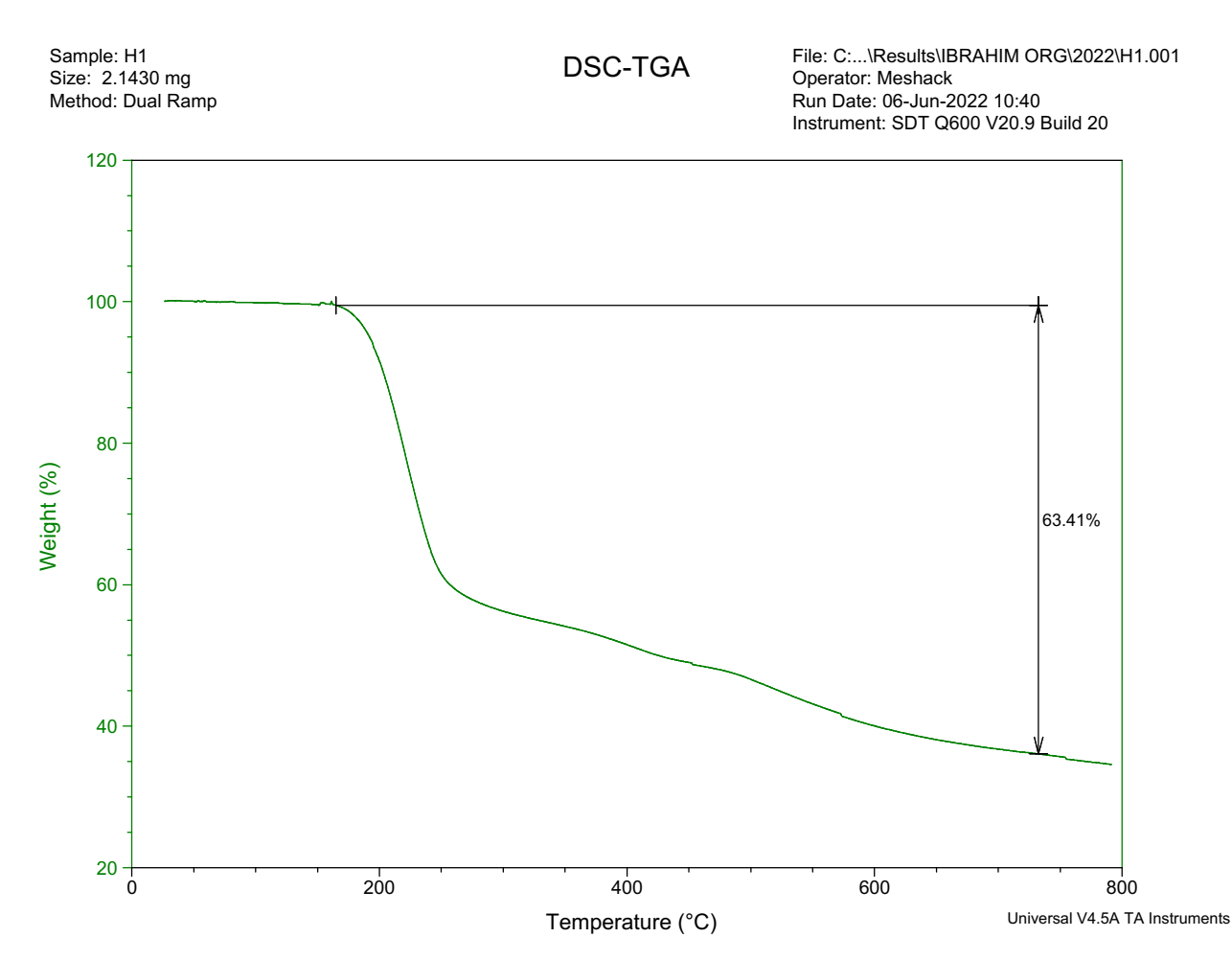


**Figure S10:** Thermogram of the Schiff base ligand, **HL** recorded in inert (N_2_) environment


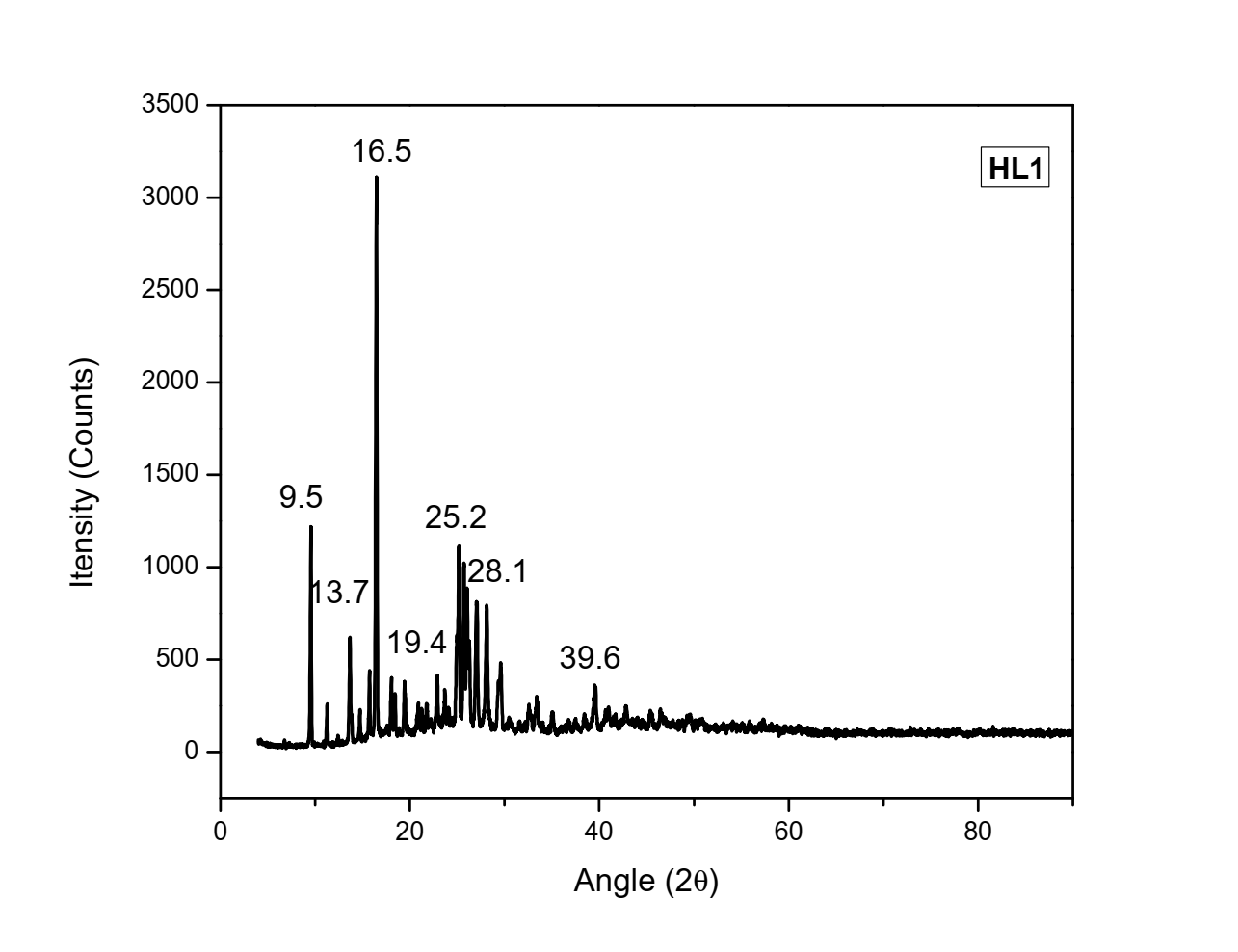


**Figure S11:** Powder X-ray diffraction spectrum of the Schiff base ligand, **HL**


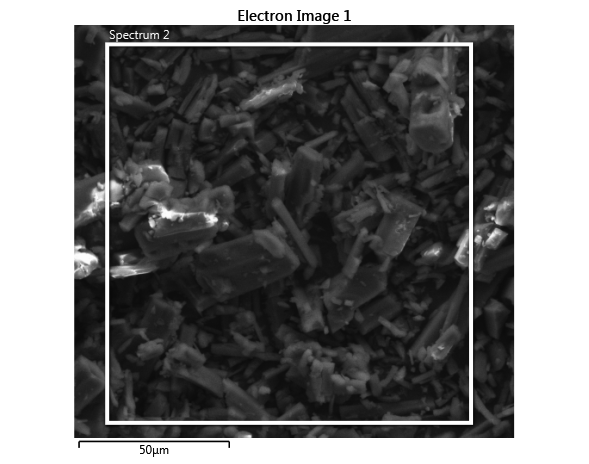


**Figure S12:** Scanning electron microscopy image of the Schiff base ligand, **HL** obtained at magnification of 50 μM

**[M+H]^+^**

**Calc. = 243.0770**

**Found = 243.0844**

**Figure S13:** Mass spectrum of the Schiff base ligand, **HL** obtained using HRMS


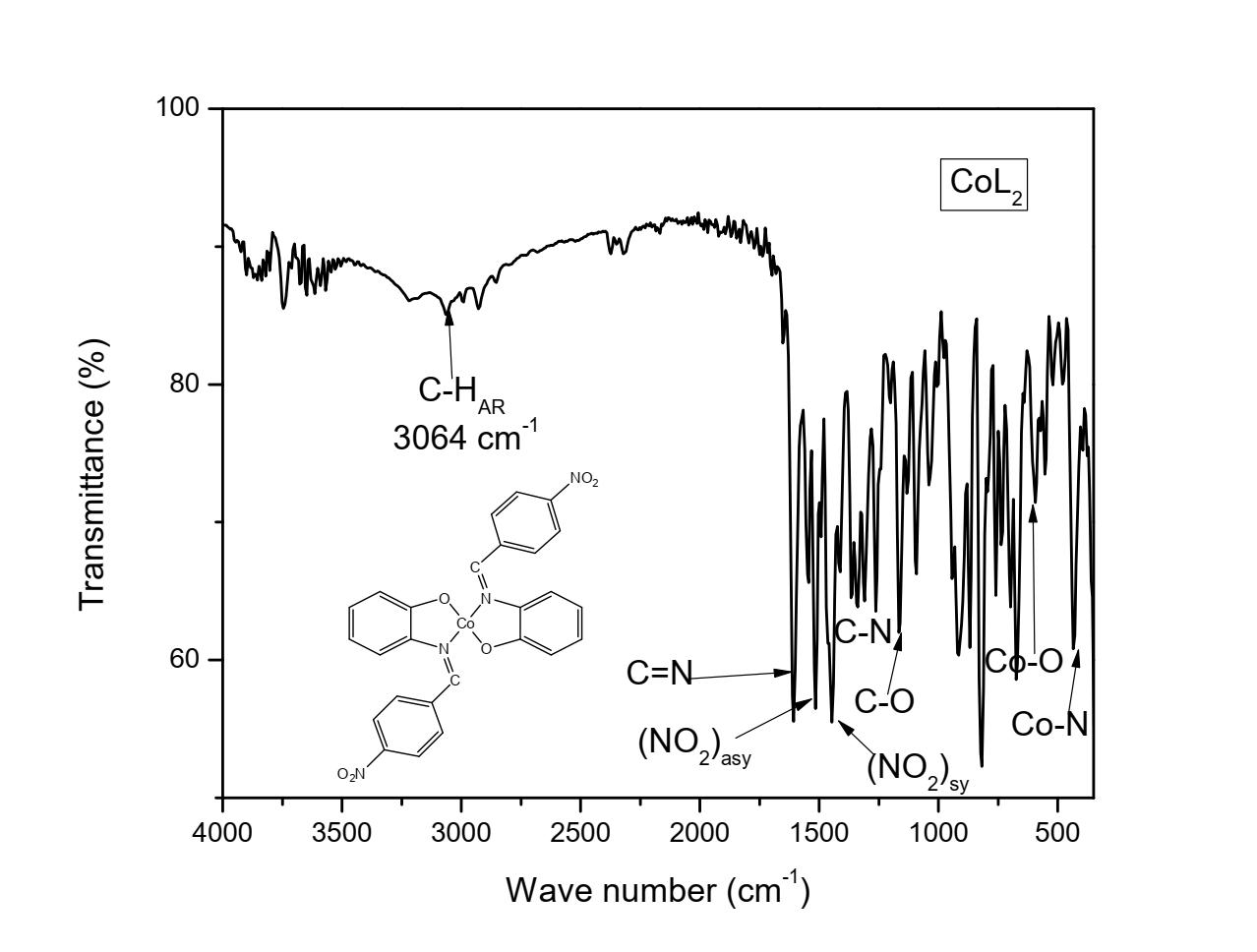


**Figure S14:** FTIR spectrum of **CoL_2_** recorded in solid state using ATR method


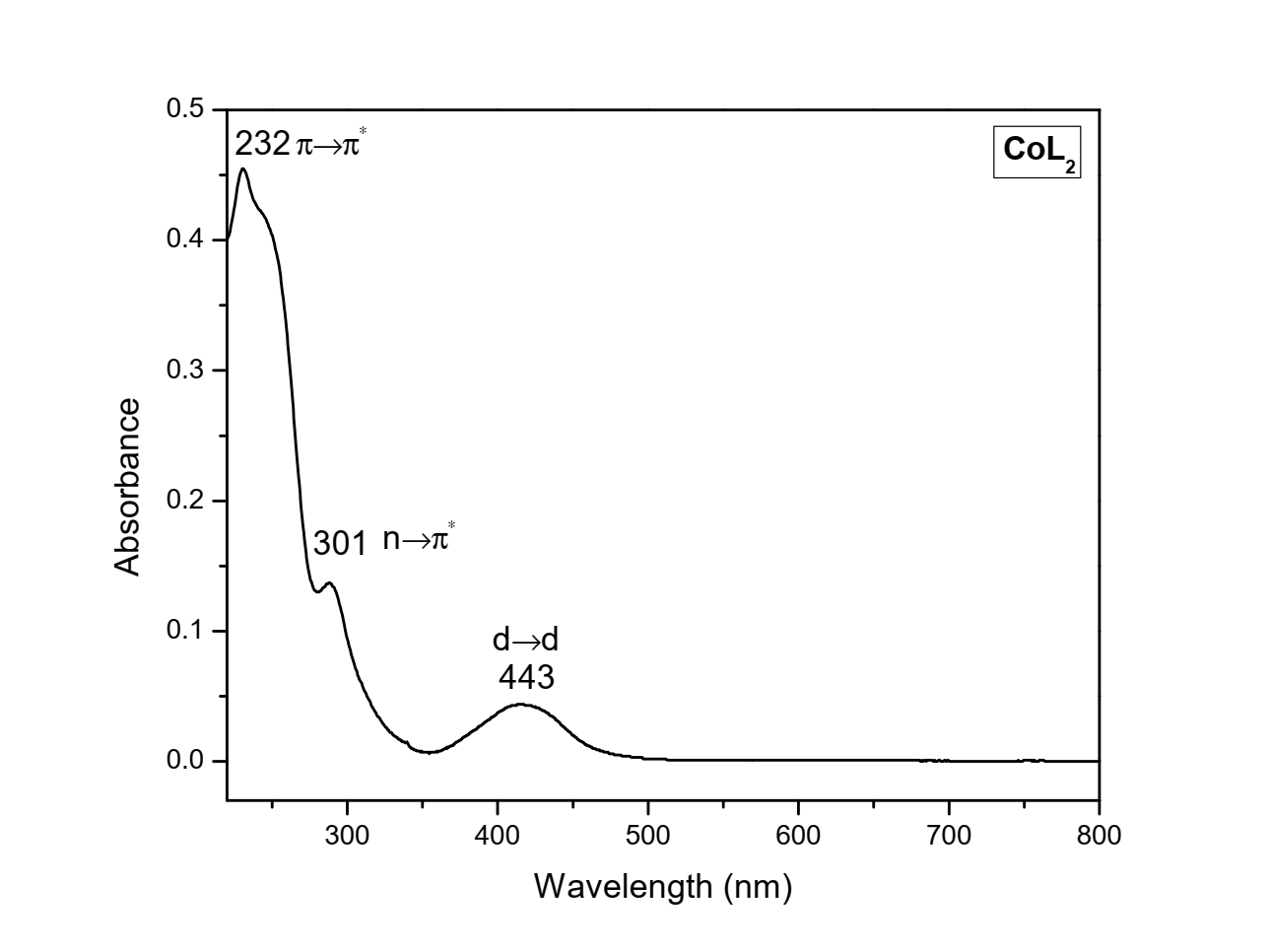


**LMCT**

**Figure S15:** UV-Vis spectrum of **CoL_2_** recorded in DMSO (10^-3^ M) at 273 K


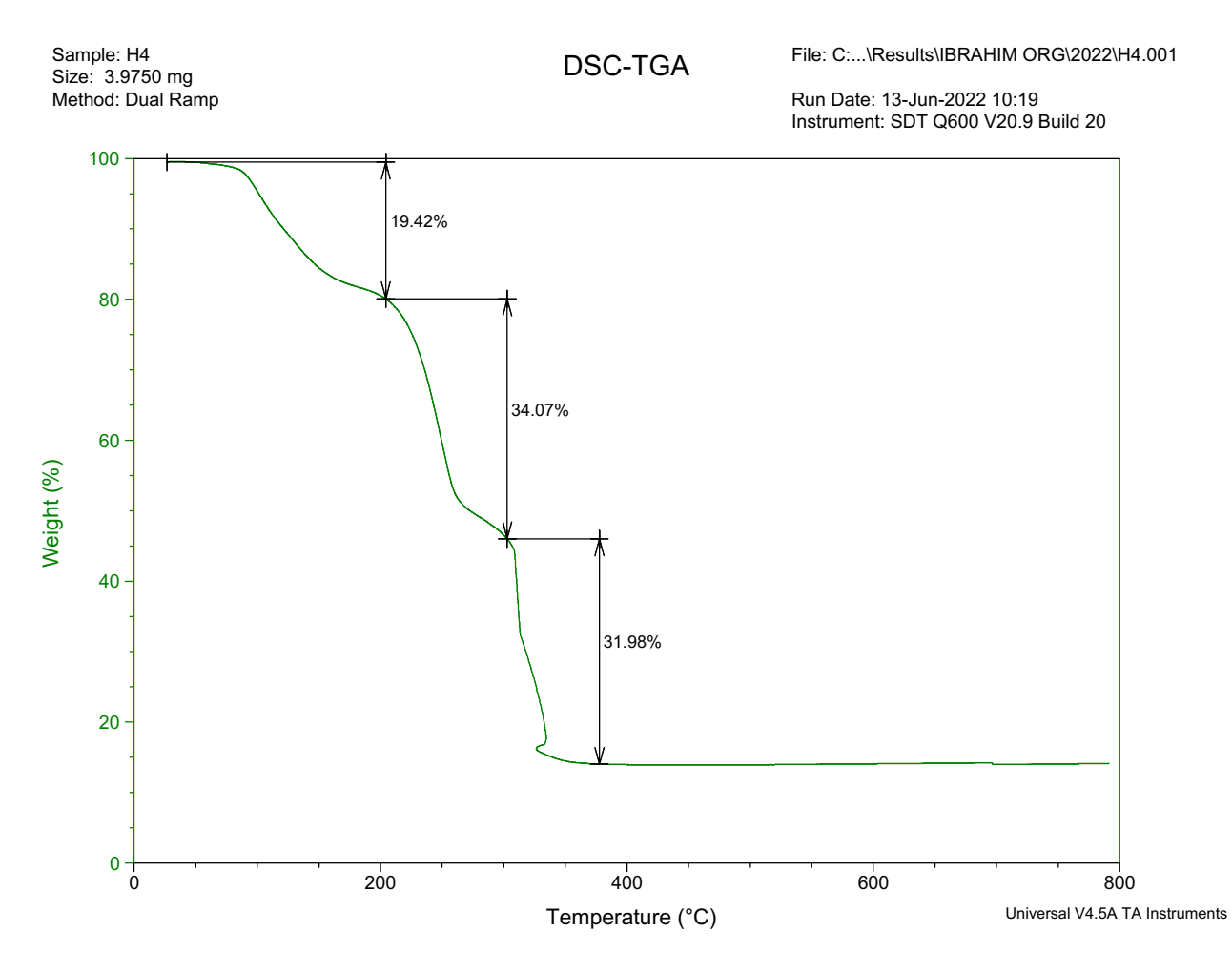


**C_13_H_10_N_2_O_3_**

**C_13_H_10_N_2_O_3_**

**2H_2_O**

**Figure S16:** Thermograph of **CoL_2_** recorded in inert (N_2_) environment


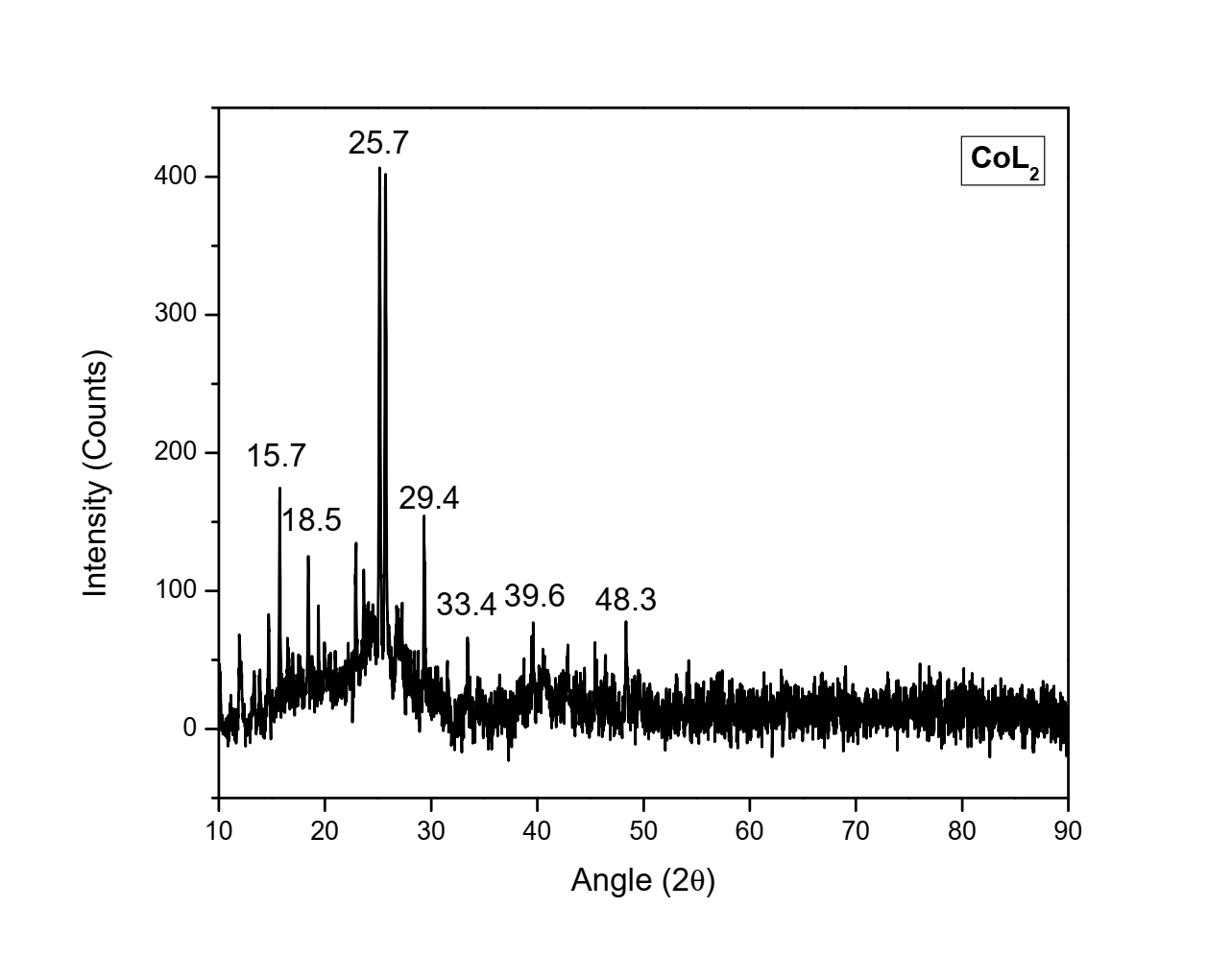


**Figure S17:** PXRD spectrum of **CoL_2_**_._


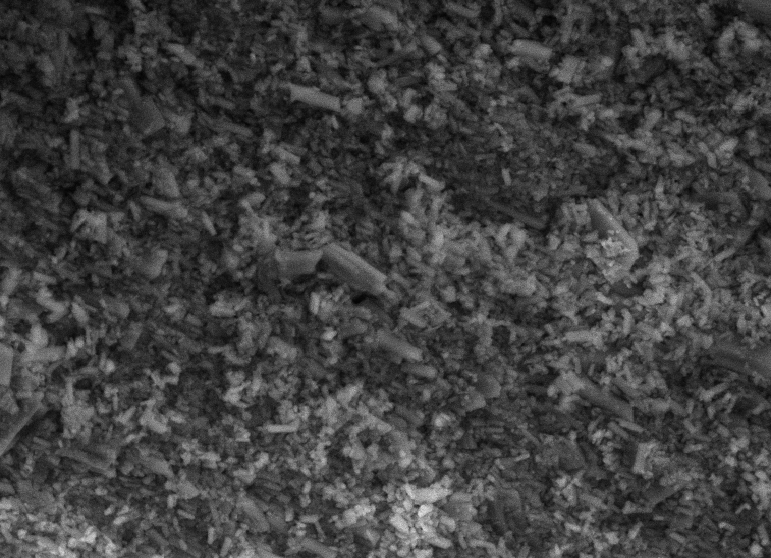


**Figure S18:** SEM surface morphology image of **CoL_2_** obtained at magnification of 20 μM


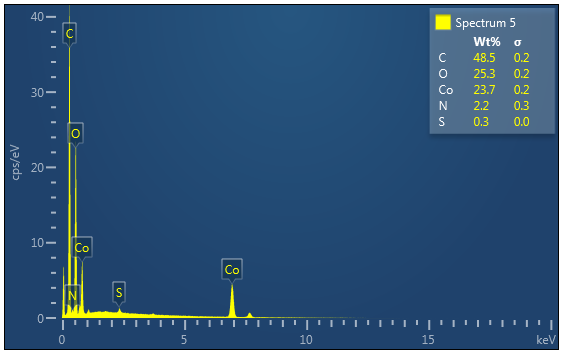


**Figure S19:** EDX micrograph of **CoL_2_**

C_20_H_14_CoN_2_O_2_^+^

C_13_H_9_CoN_2_O_4_^+^

C_13_H_9_CoNO_2_^+^

C_7_H_5_NO_2_^+^

C_13_H_9_N_2_O_2_^+^

**[M+H]^+^**

**Calc. = 542.0637**

**Found = 542.2303**

**Figure S20:** Mass spectrum of **CoL_2_** obtained using HRMS


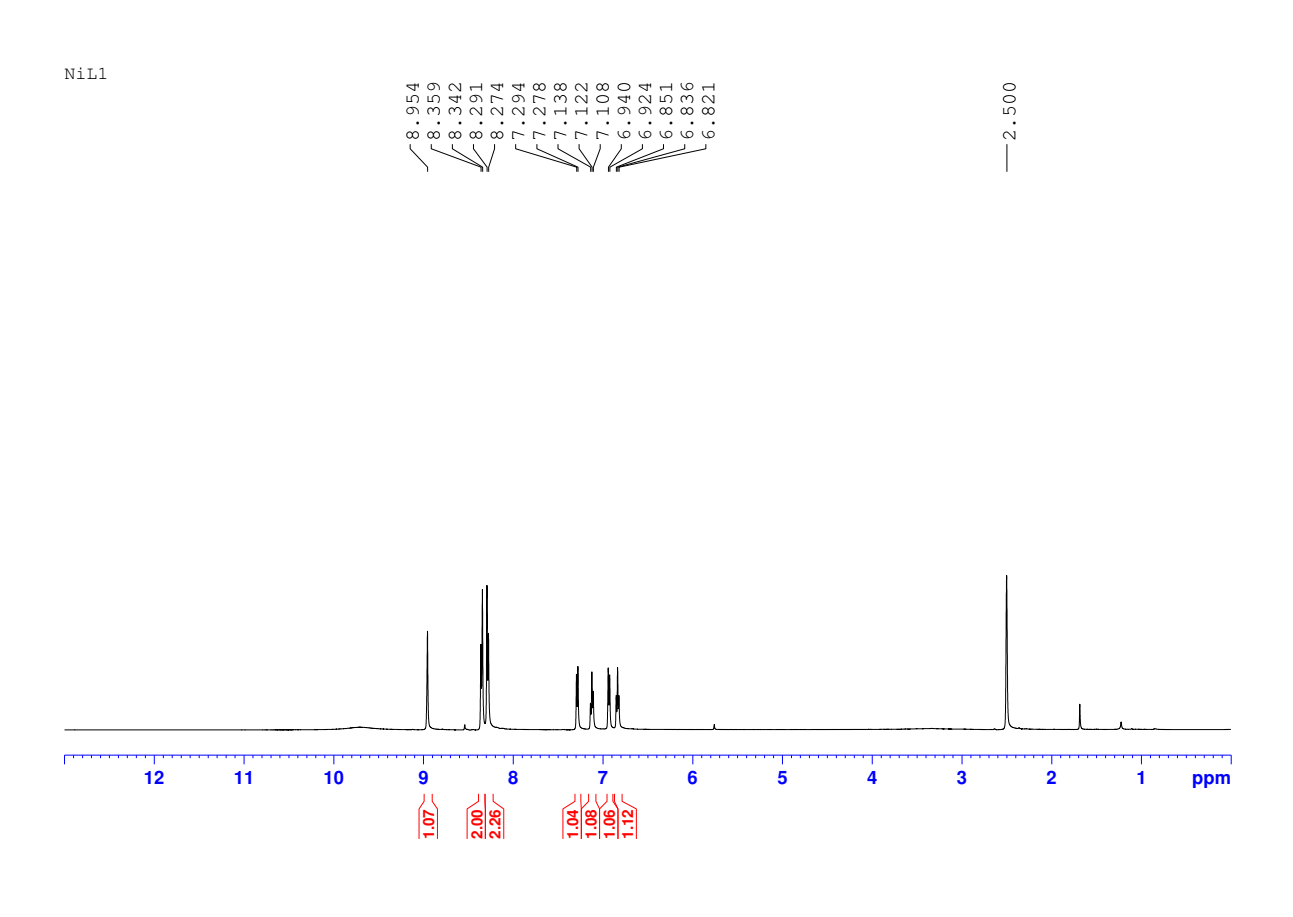


**d,e,f,g**

**b,c**

**a**

**Figure S21:** ^1^H NMR spectrum of **NiL_2_** recorded at 273 K using (500 MHz, DMSO-*d_6_*)


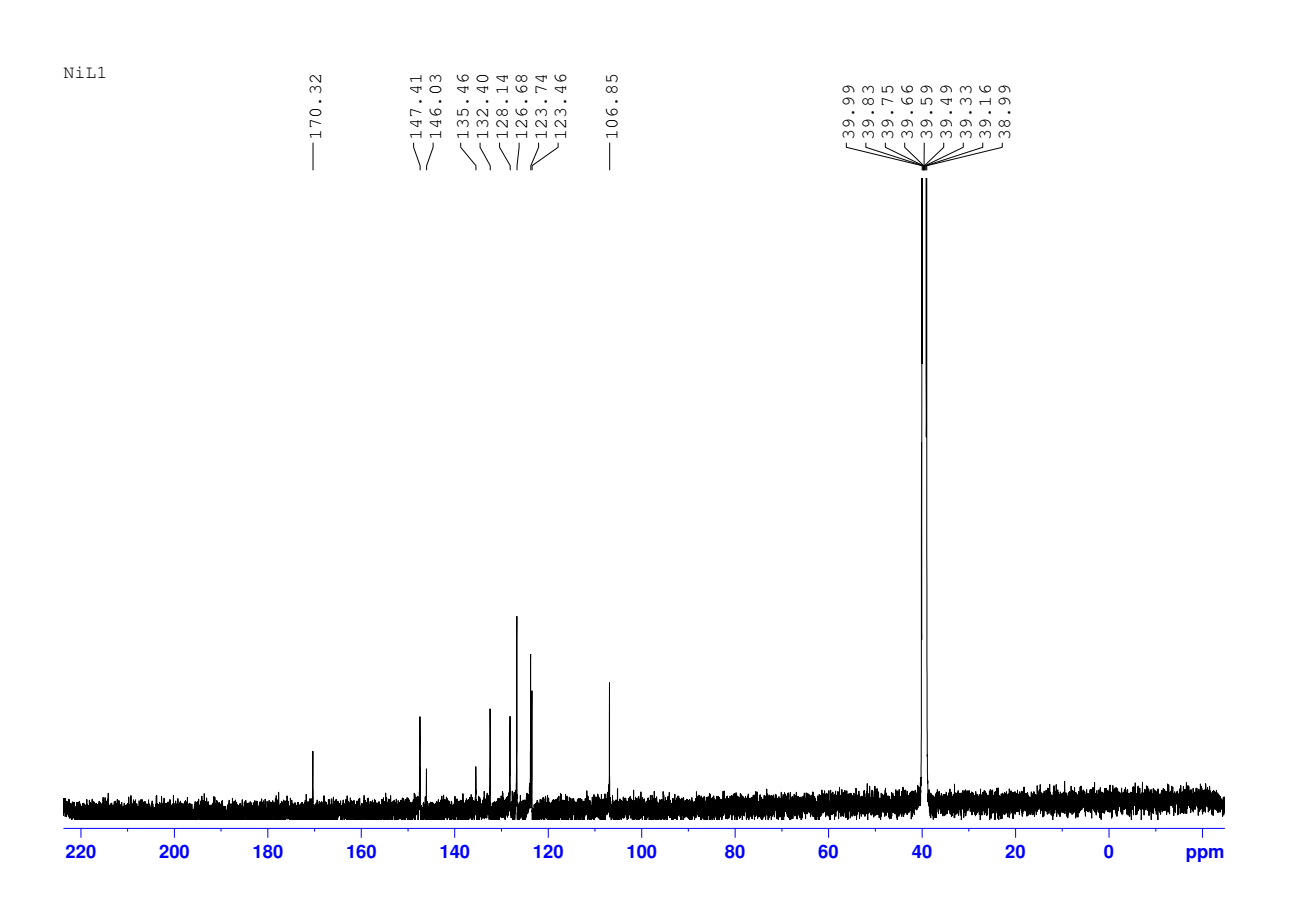


**K**

**d,e,f,h,I,j**

**b,c**

**a**

**Figure S22:** ^13^C NMR spectrum of **NiL_2_** recorded at 273 K using (125 MHz, DMSO-*d_6_*).


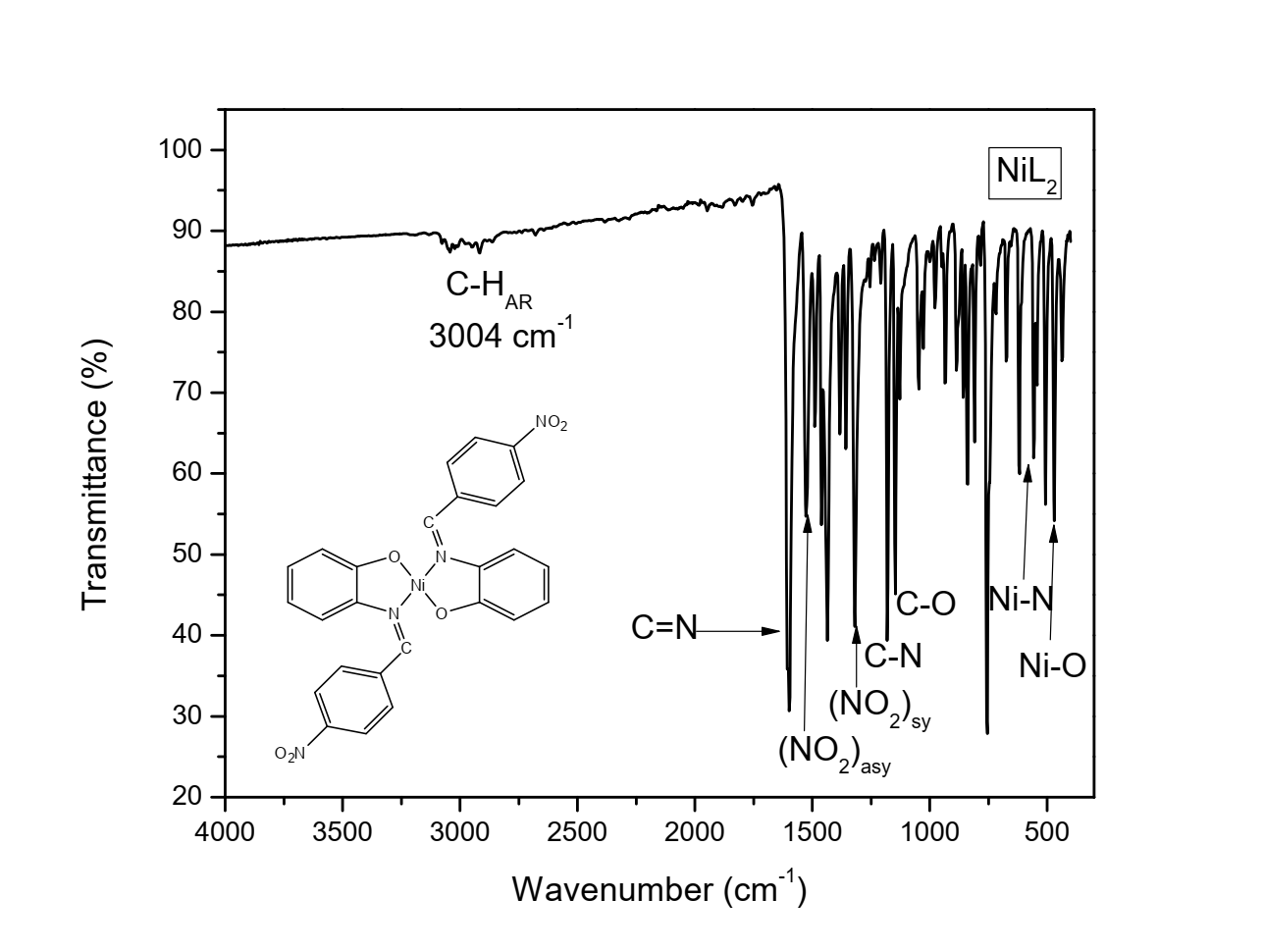


**Figure S23:** FTIR spectrum of **NiL_2_** recorded in solid state using ATR method


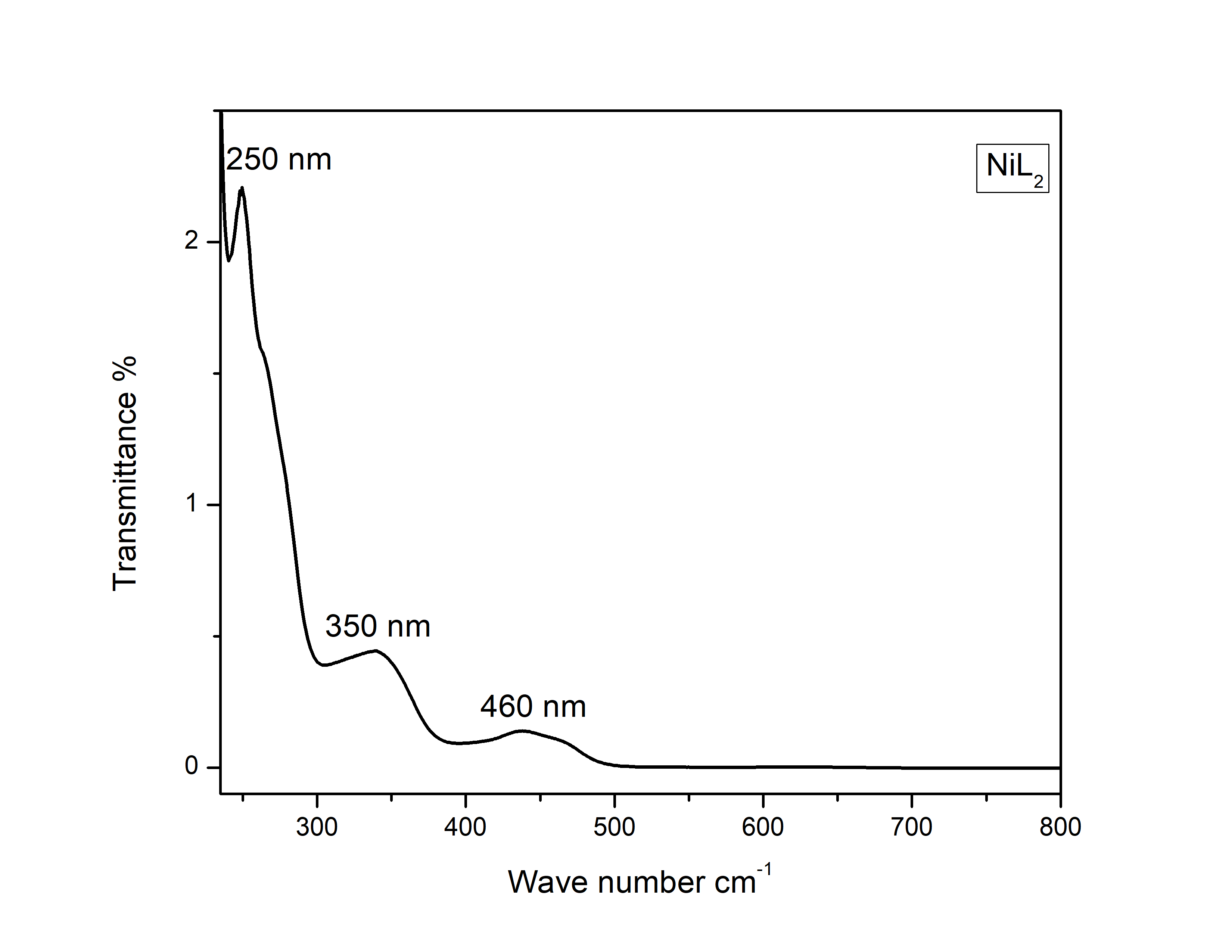


**LMCT**

**n→π^*^**

**π→π^*^**

**Figure S24:** UV-Vis spectrum of **NiL_2_** recorded in DMSO (10^-3^ M) at 273 K


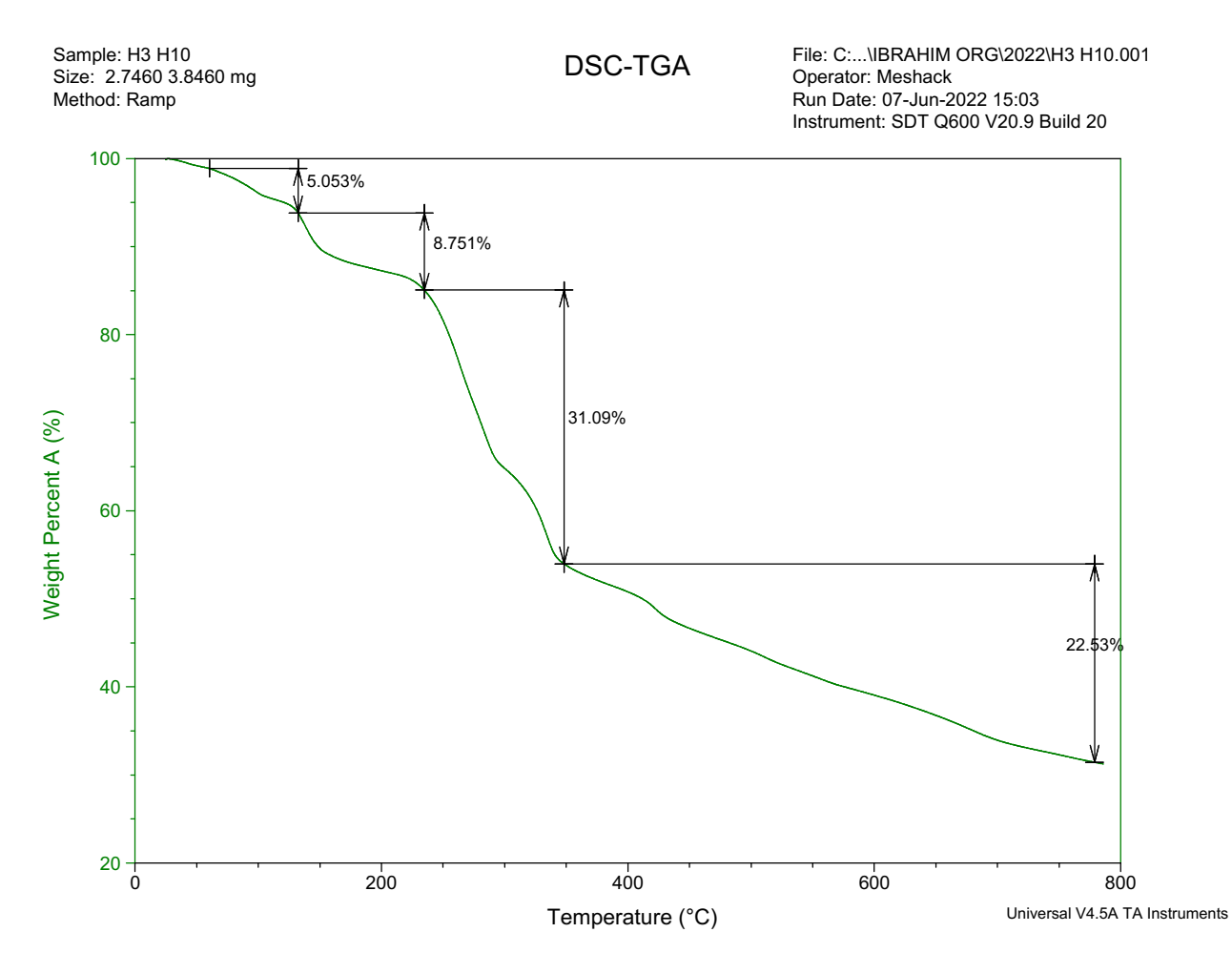


**C_13_H_10_N_2_^+^**

**C_13_H_10_N_2_O_3_**

**2H_2_O**

**Figure S25:** Thermograph of **NiL_2_** recorded in inert (N_2_) environment.


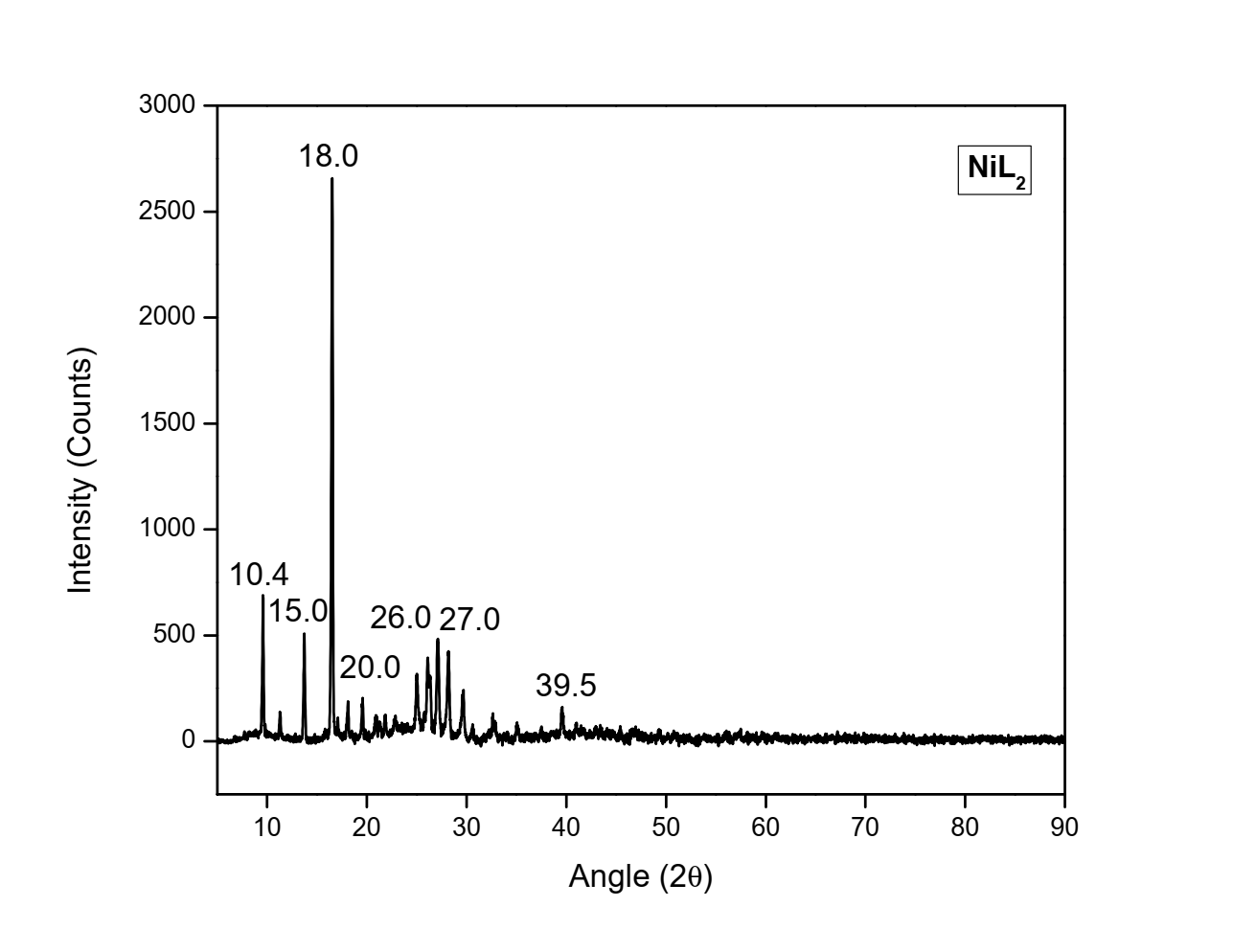


**Figure S26:** PXRD spectrum of **NiL_2_**.


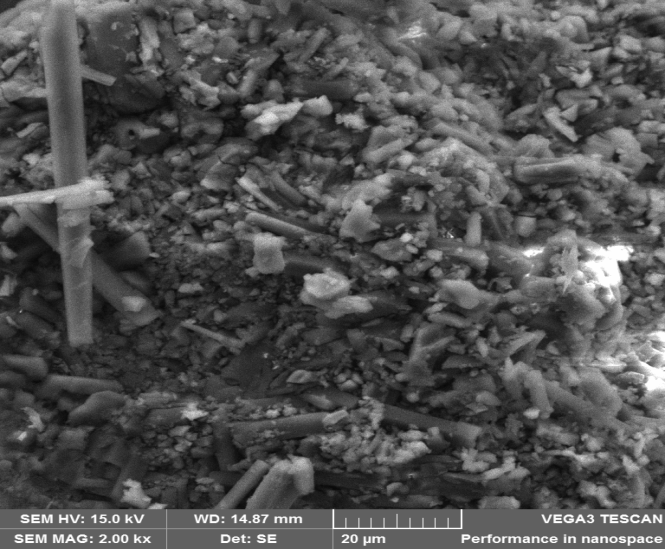


**Figure S27**: SEM image of **NiL_2_** obtained at magnification of 20 μM


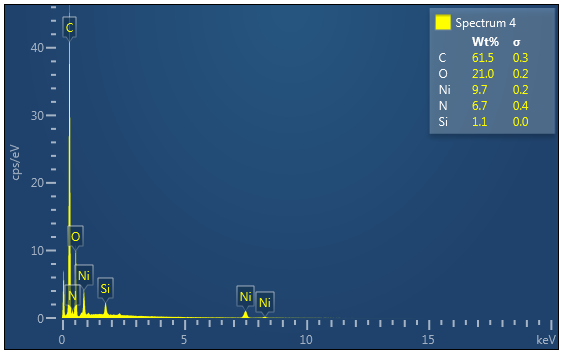


**Figure S28**: EDX micrograph of **NiL_2_**

C_25_H_18_N_2_NiO_2_^+^

C_13_H_9_N_2_NiO_4_^+^

C_7_H_5_NO_2_^+^

**[M+H]^+^**

**Calc. = 542.1457**

**Found = 542.2307**

**Figure S29:** Mass spectrum of **NiL_2_** recorded using HRMS


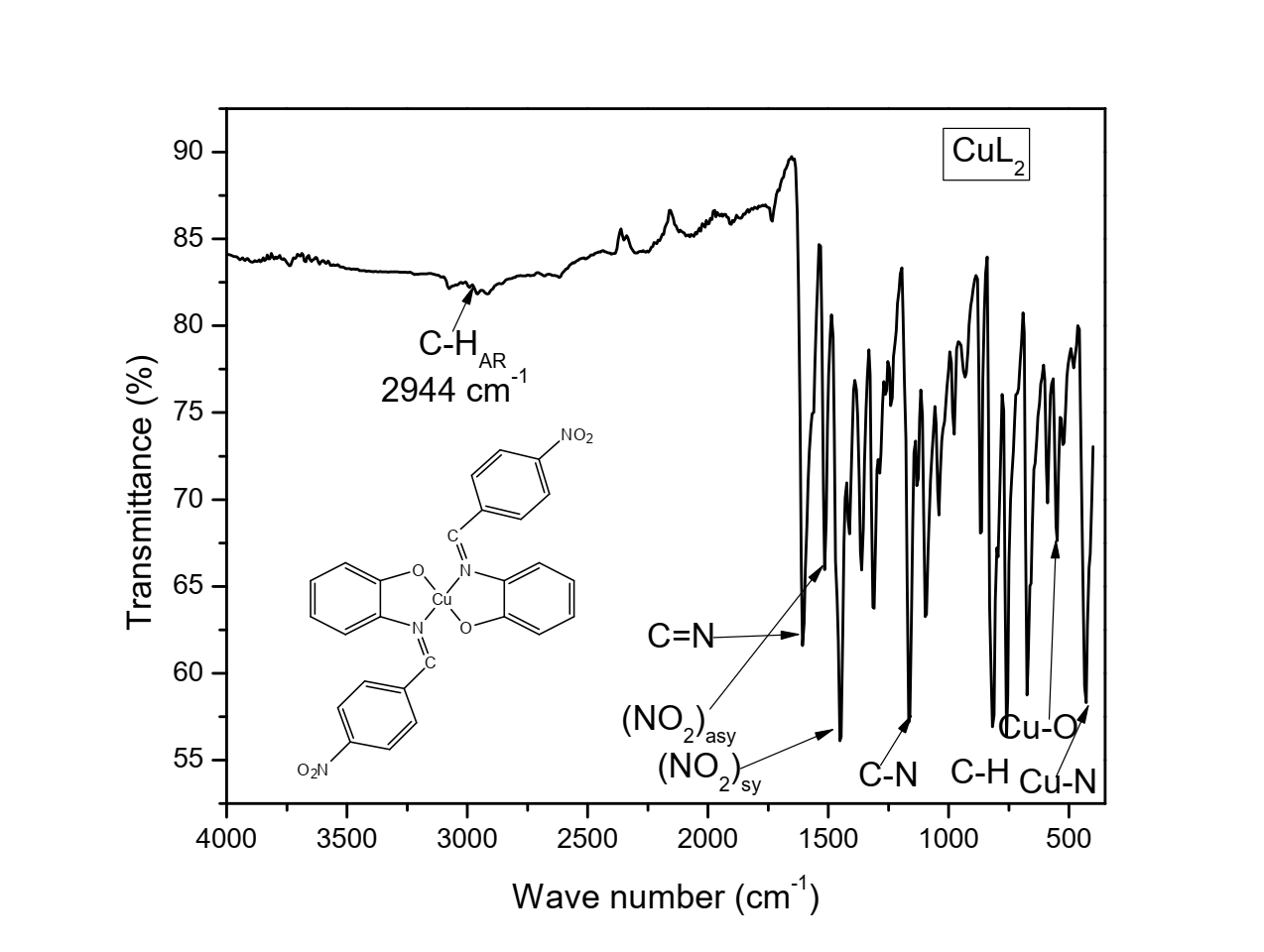


**Figure S30:** FTIR spectrum of **CuL_2_** obtained in solid-state using ATR method


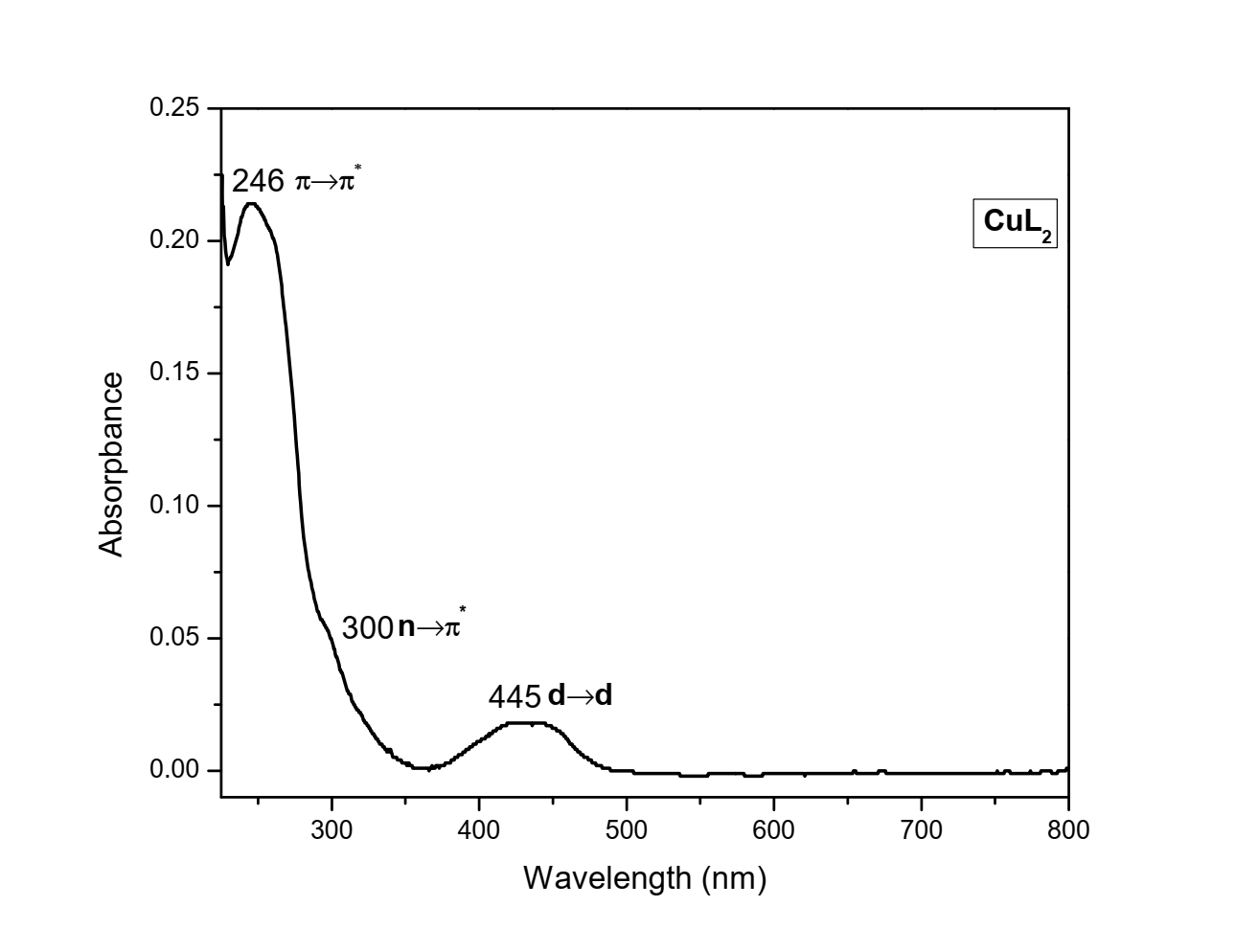


**Figure S31:** UV-Vis spectrum of **CuL_2_** recorded in DMSO (10^-3^ M) at 273 K


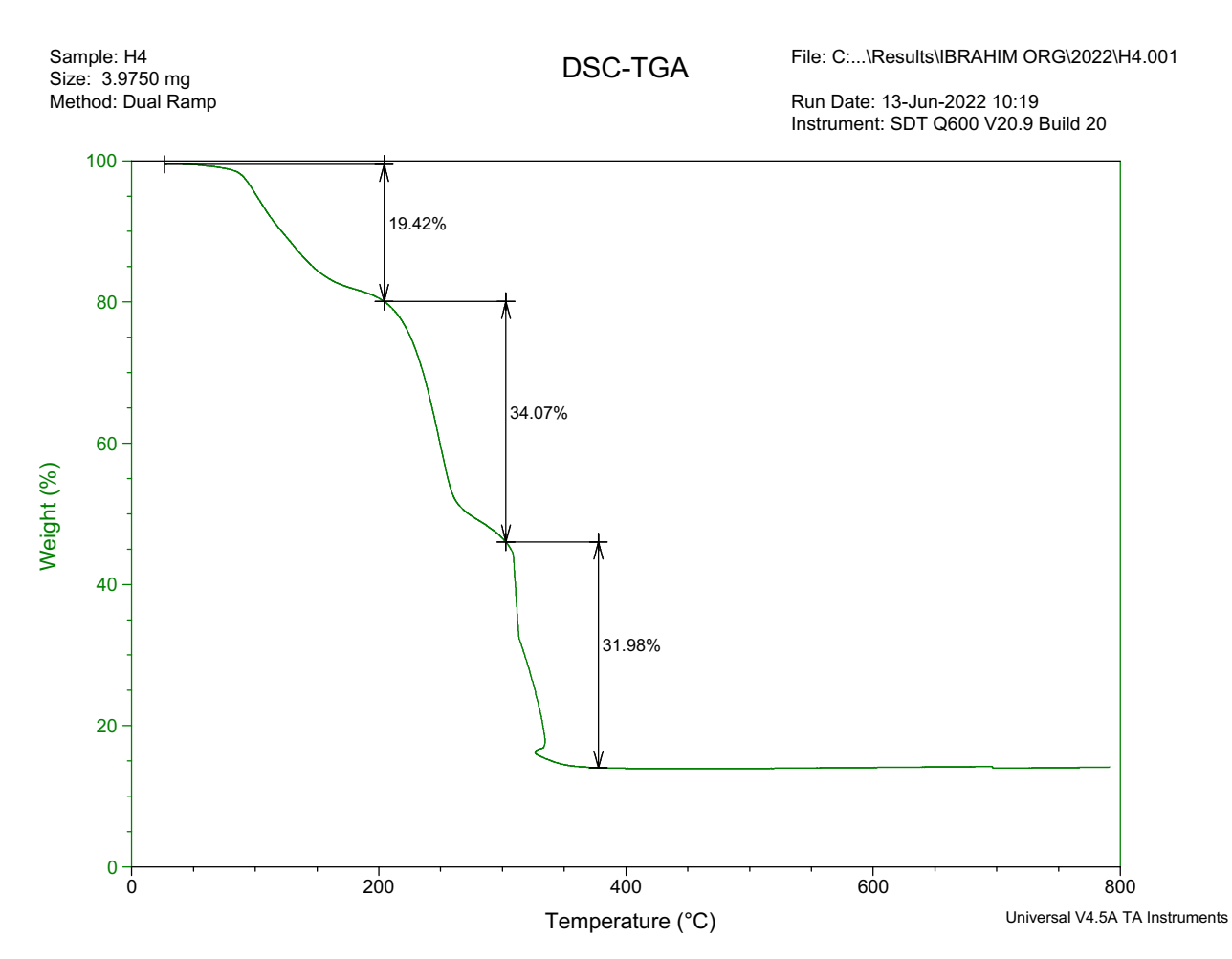


**C_13_H_10_N_2_O_3_**

**C_13_H_10_N_2_O_3_**

**2H_2_O**

**Figure S32:** Thermograph of **CuL_2_** recorded in inert (N_2_) environment


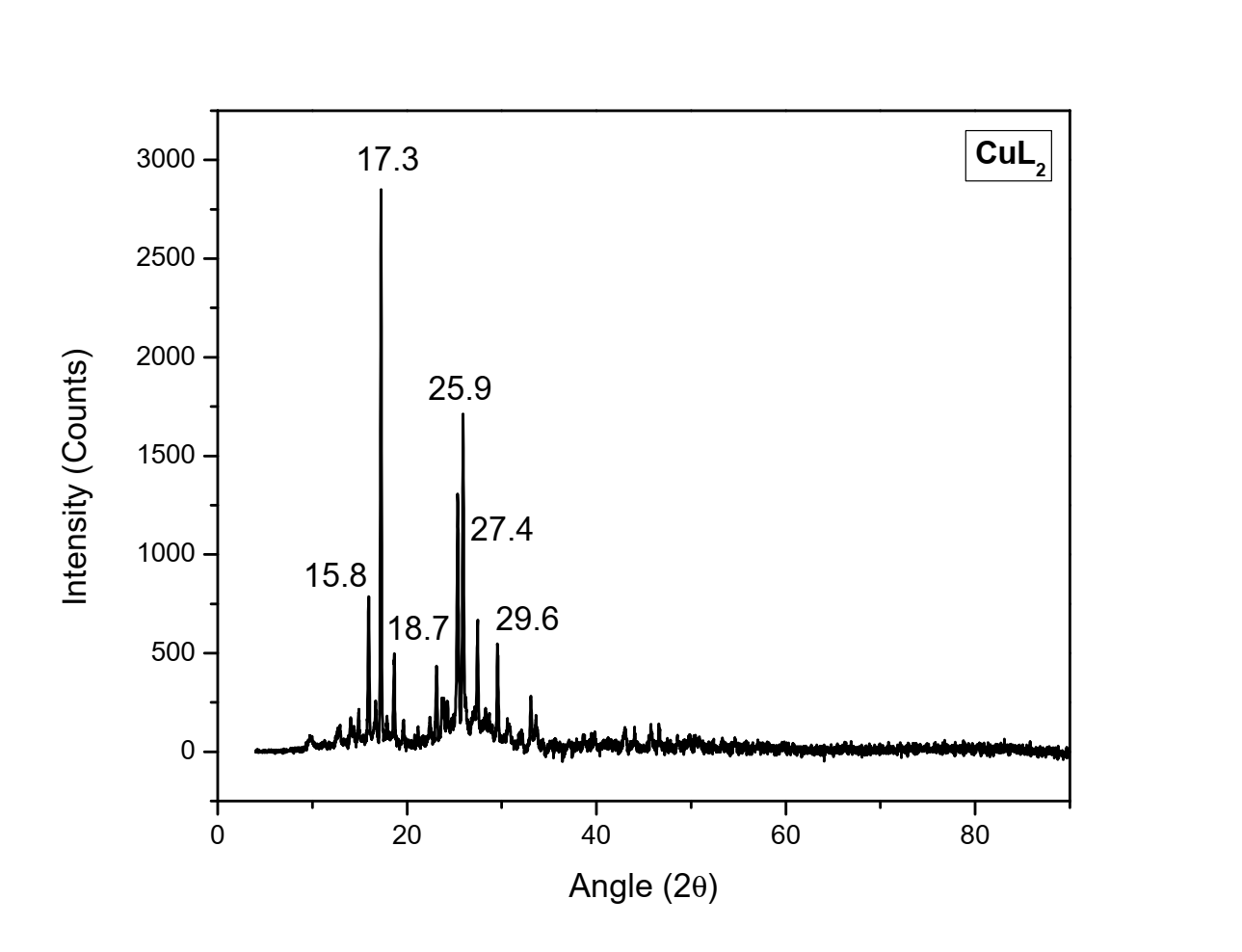


**Figure S33:** PXRD spectrum of **CuL_2_**.


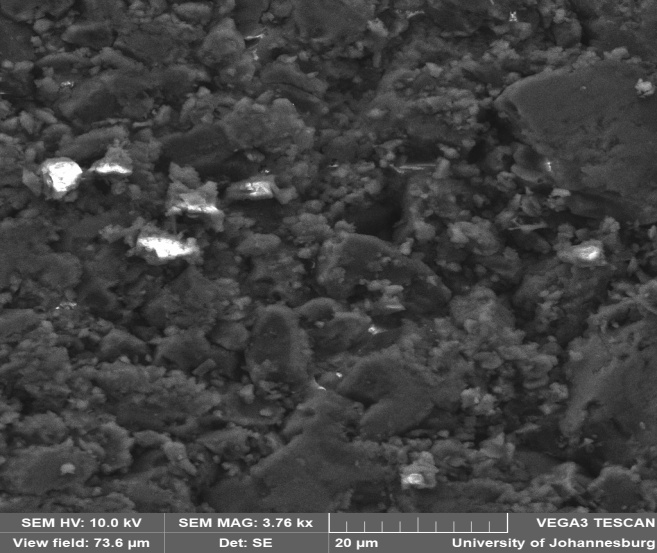


**Figure S34:** SEM surface morphology of **CuL_2_** obtained at magnification of 20 μM


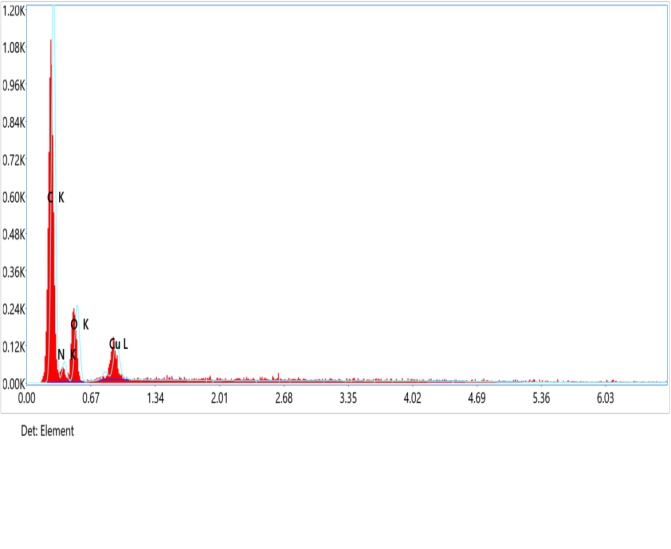


**Figure S35:** EDX micrograph of **CuL_2_**

C_14_H_10_CuN_4_O_6_^+^

**[M]^+^**

**Calc.= 545.0522**

**Found =545.0084**

**Figure S36:** Mass spectrum of **CuL_2_** recorded using HRMS


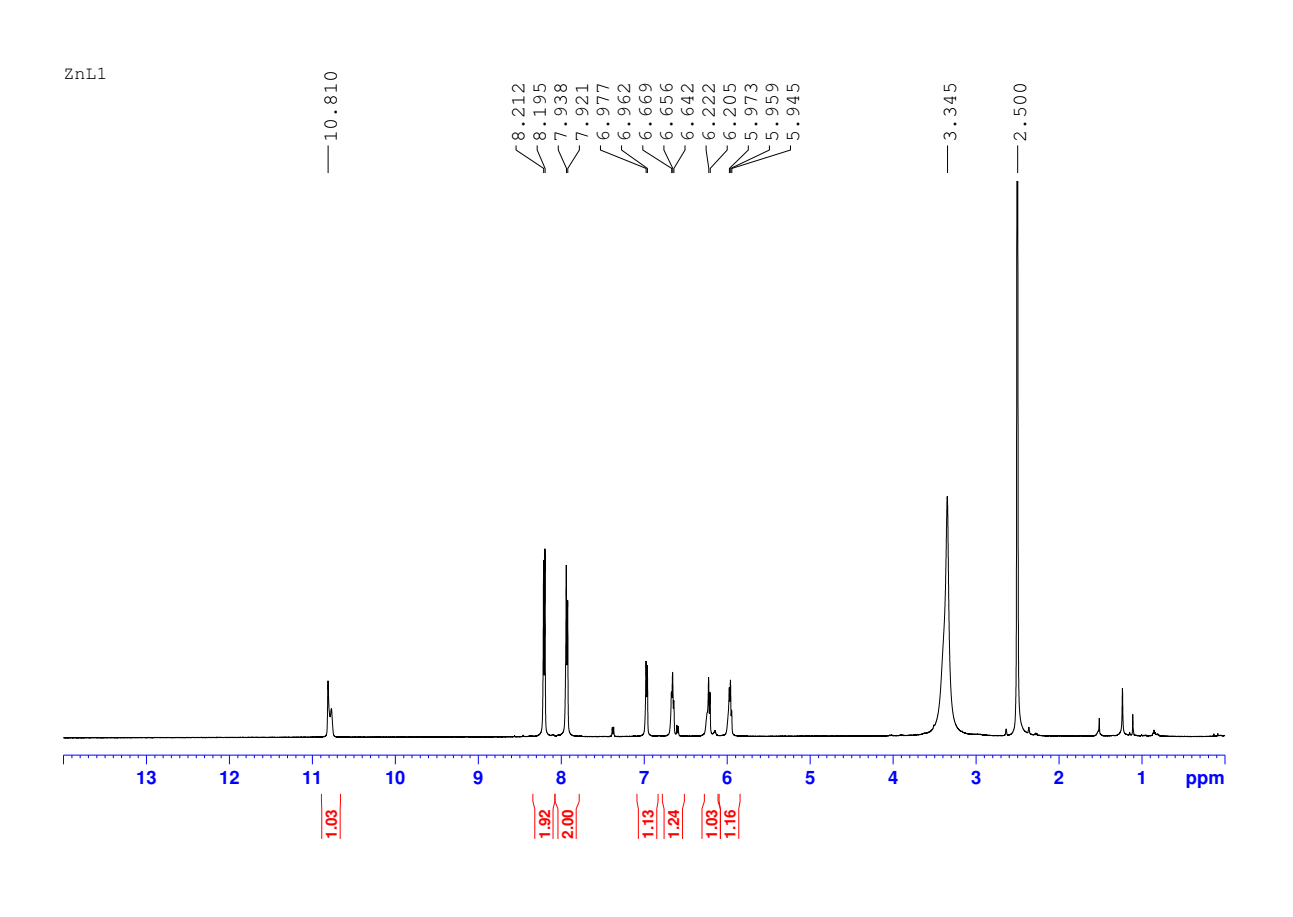


**d,e,f,g**

**b,c**

**a**

**Figure S37:**^1^H NMR spectrum of **ZnL_2_** recorded at 273 K using (500 MHz, DMSO-*d_6_*).


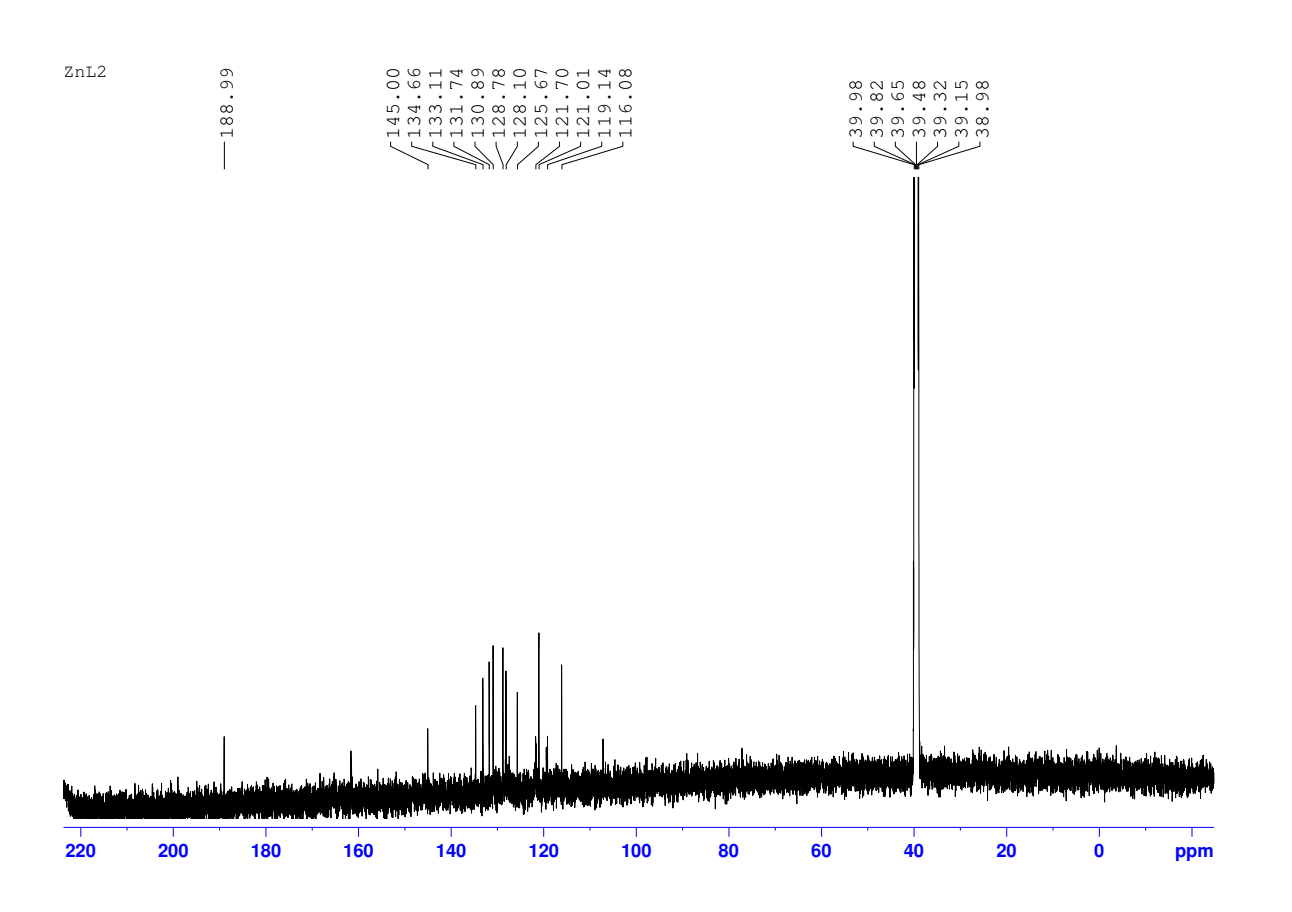

**c,d,e,f,g,h,I,j,k**

**b**

**a**

**Figure S38:** ^13^C NMR spectrum of **ZnL_2_** recorded at 273 K using (125 MHz, DMSO-*d_6_*).


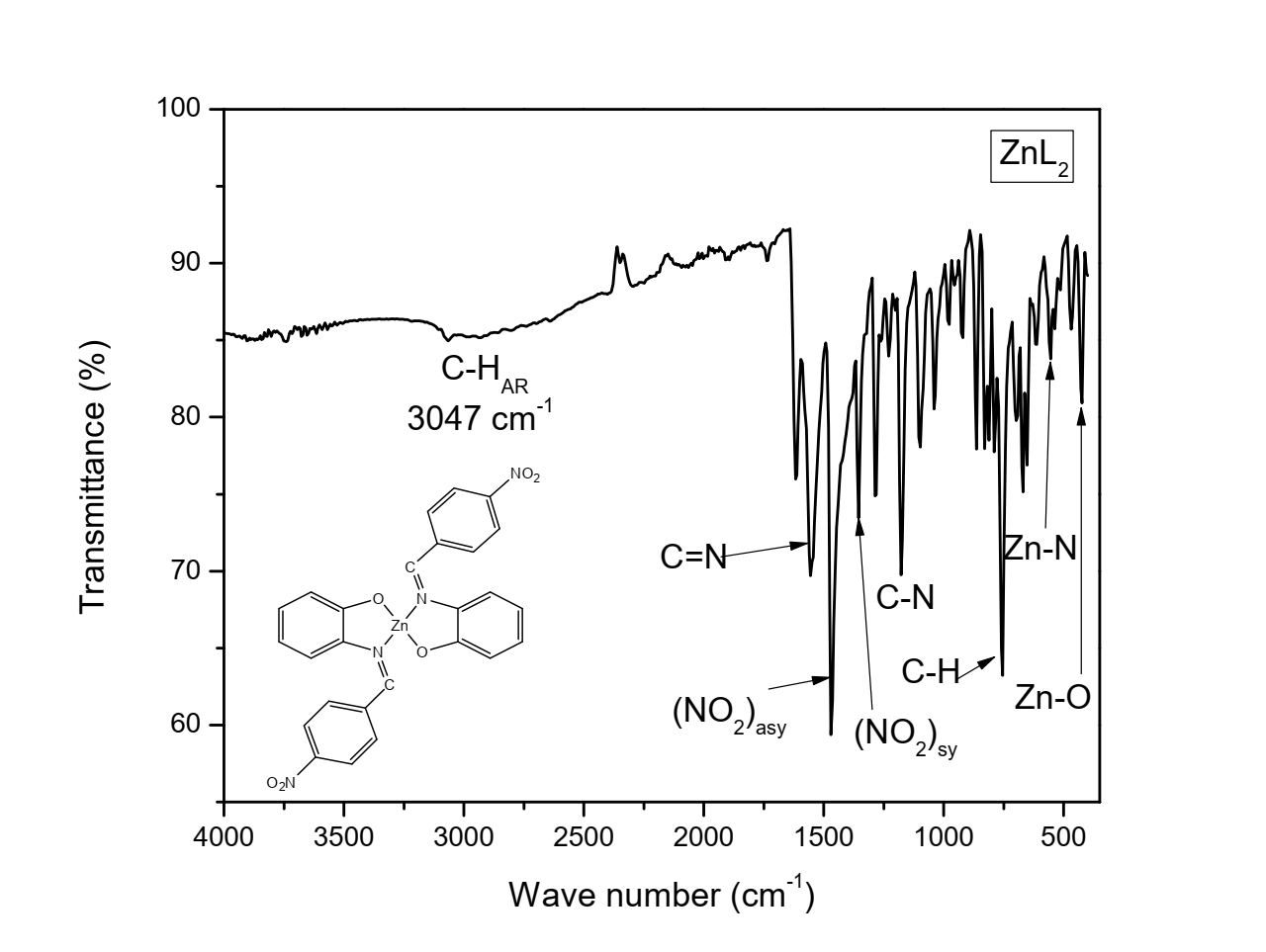


**Figure S39:** FTIR spectrum of **ZnL_2_** recorded in solid-state using ATR method


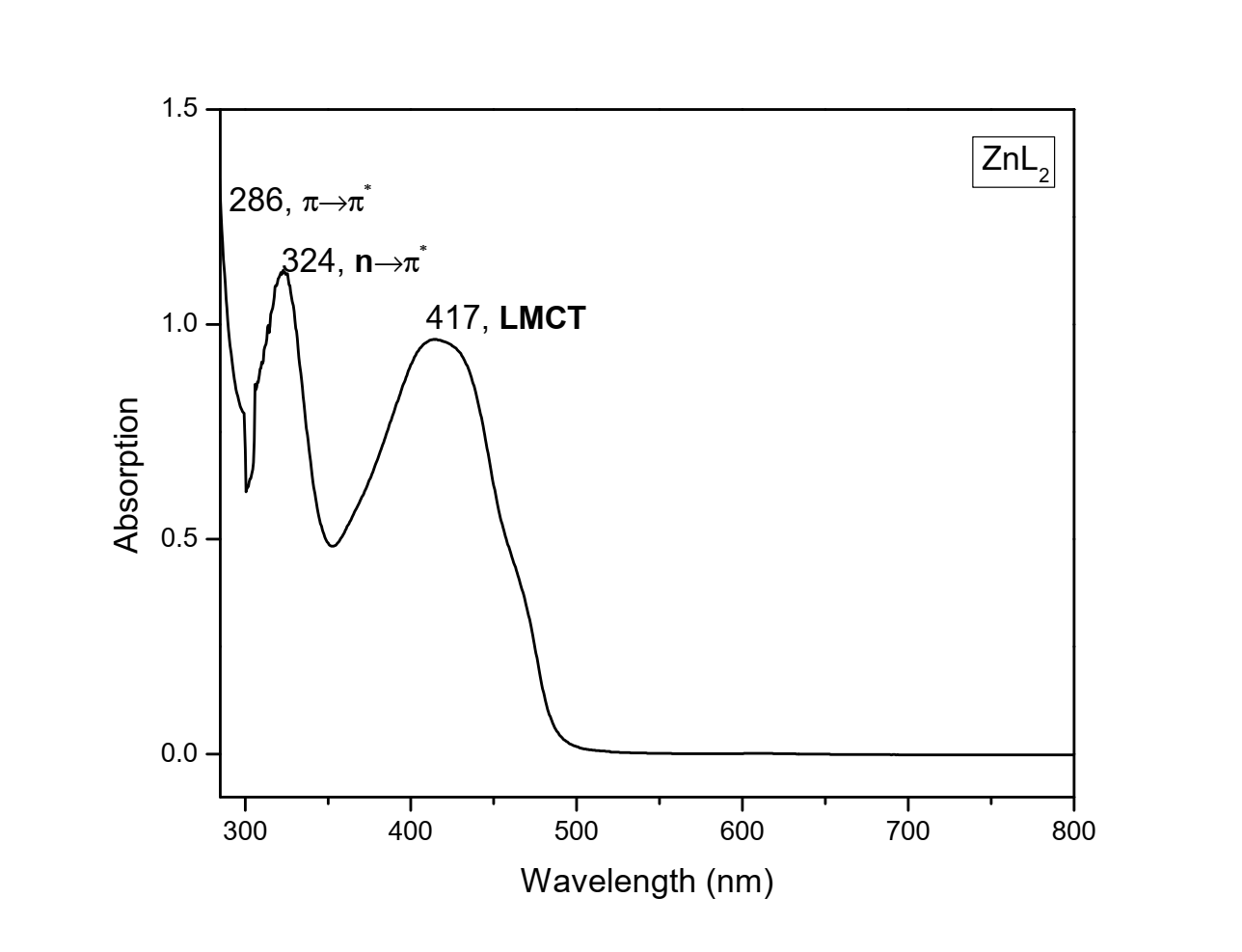


**Figure S40:** UV-Vis spectrum of **ZnL_2_** recorded in DMSO (10^-3^ M) at 273 K


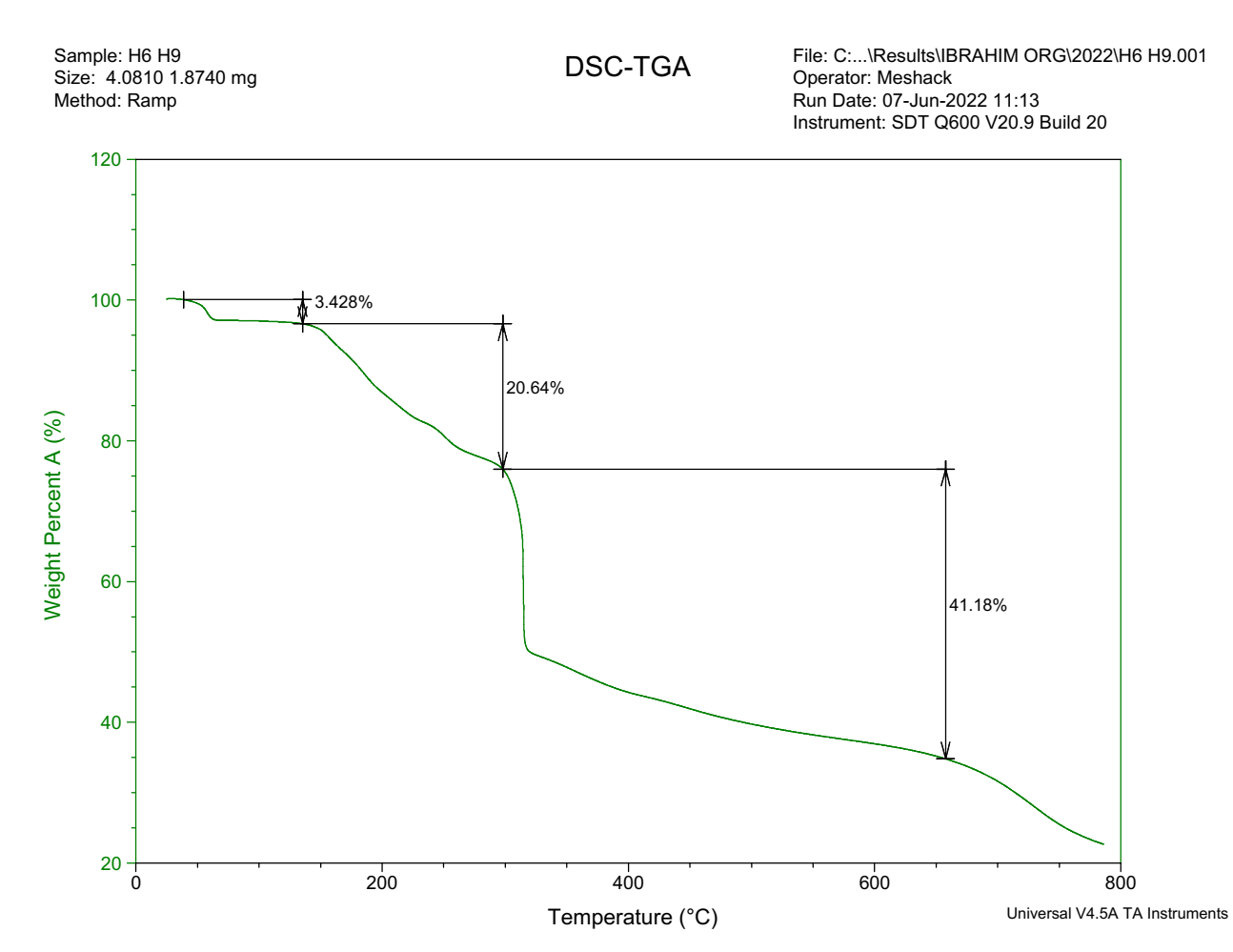


**C_13_H_10_N_2_O_3_**

**C_13_H_10_N_2_^+^**

**Moisture**

**Figure S41:** Thermograph of **ZnL_2_** recorded in inert (N_2_) environment


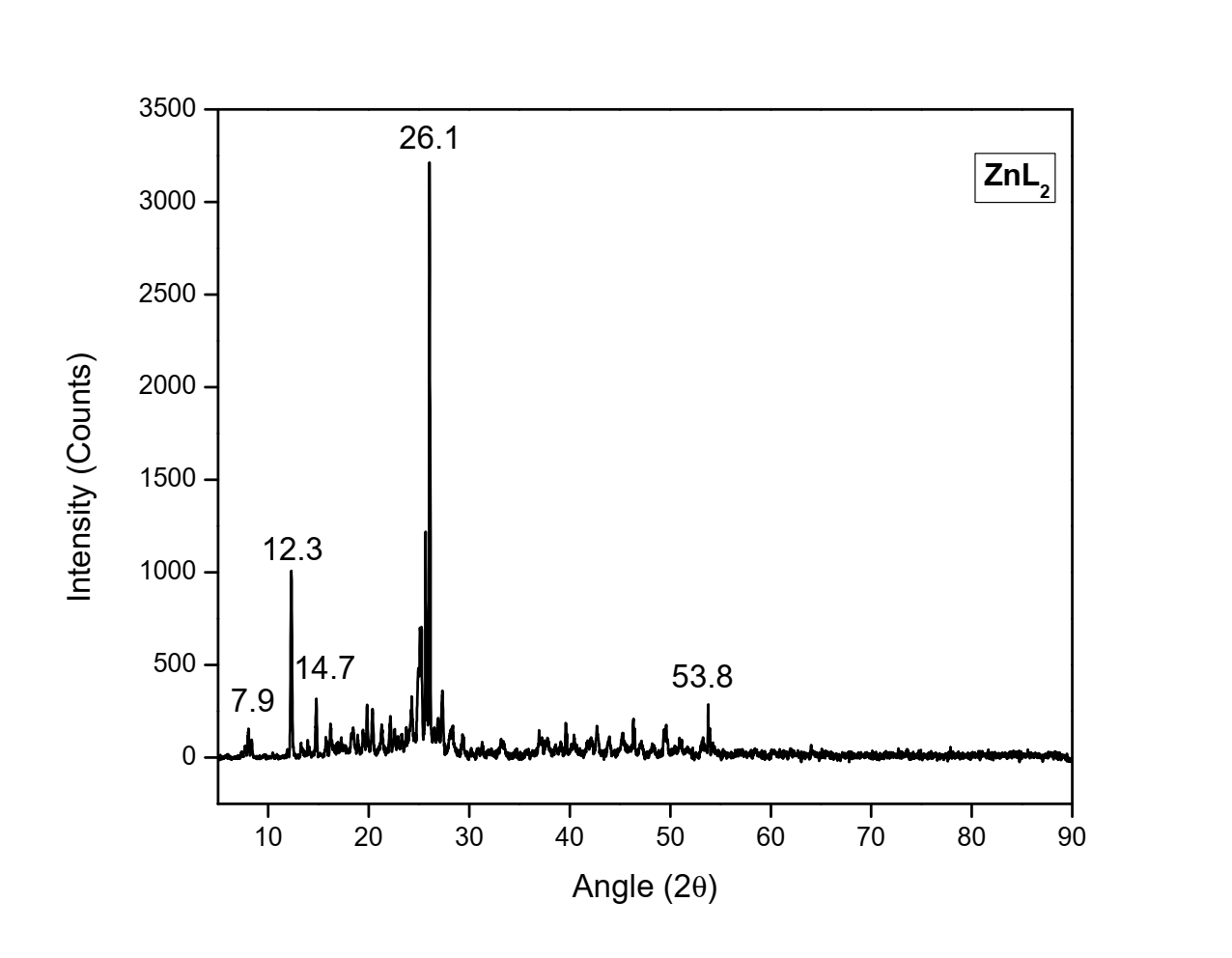


**Figure S42:** PXRD spectrum of **ZnL_2_**.


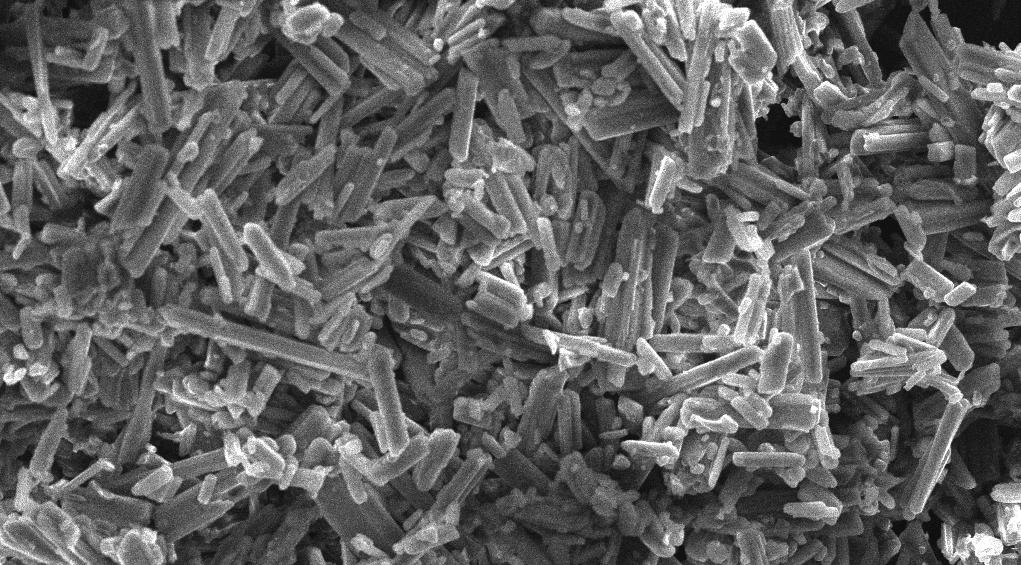


**Figure S43:** SEM surface morphology image of **ZnL_2_** obtained at magnification of 20 μM


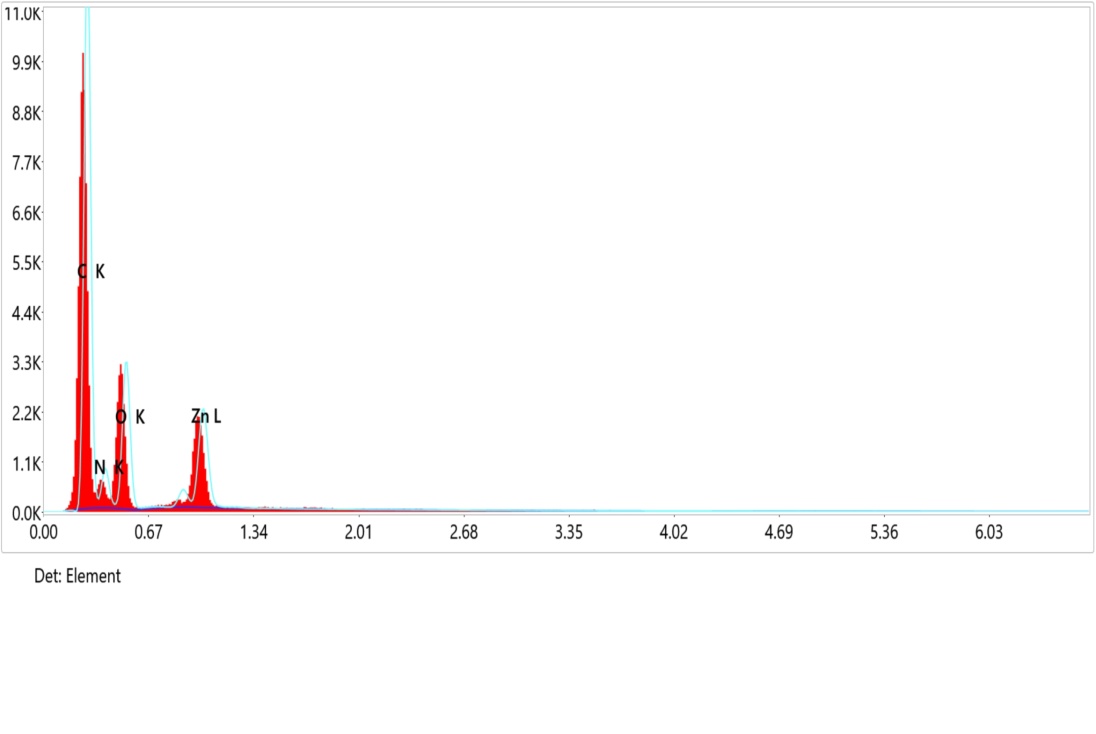


**Figure S44:** EDX micrograph of **ZnL_2_**


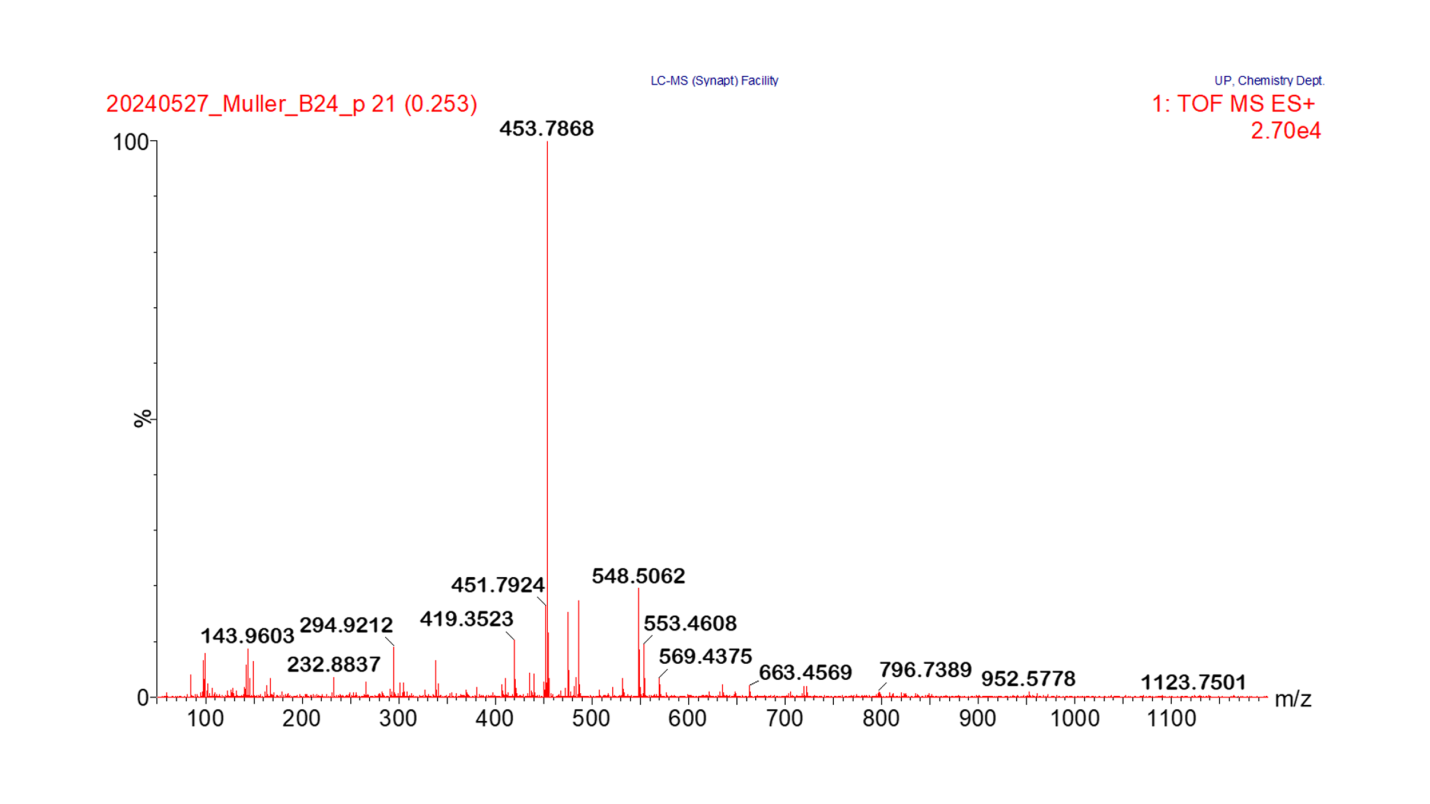


C_26_H_18_N_2_O_2_Zn^+^

C_13_H_9_N_2_O_3_Zn^+^

C_7_H_5_N_2_O_2_

**[M+H]^+^**

**Calc. = 548.8323**

**Found = 548.5062**

**Figure S45:** Mass spectrum of **ZnL_2_**

**Table S1**: Crystal data and structure refinement for the ligand, HL

| **Identification code** | **HL** |
| --- | --- |
| Empirical formula | C_13_H_10_N_2_O_3_ |
| Formula weight | 239.232 |
| Temperature/K | 173.03 |
| Crystal system | Orthorhombic |
| Space group | P2_1_2_1_2_1_ |
| a/Å | 4.662(2) |
| b/Å | 9.537(5) |
| c/Å | 25.170(13) |
| α/° | 90 |
| β/° | 90 |
| γ/° | 90 |
| Volume/Å^3^ | 1119.1(10) |
| Z | 4 |
| ρ_calc_g/cm^3^ | 1.420 |
| μ/mm^‑1^ | 0.101 |
| F(000) | 496.4 |
| Crystal size/mm^3^ | 0.55 × 0.2 × 0.18 |
| Radiation | Mo Kα (λ = 0.71073) |
| 2Θ range for data collection/° | 4.56 to 57.52 |
| Index ranges | -6 ≤ h ≤ 6, -11 ≤ k ≤ 12, -33 ≤ l ≤ 23 |
| Reflections collected | 5581 |
| Independent reflections | 2870 [R_int_ = 0.0518, R_sigma_ = 0.0896] |
| Data/restraints/parameters | 2870/0/164 |
| Goodness-of-fit on F^2^ | 0.977 |
| Final R indexes [I>=2σ (I)] | R_1_ = 0.0561, wR_2_ = 0.1143 |
| Final R indexes [all data] | R_1_ = 0.1066, wR_2_ = 0.1323 |
| Largest diff. peak/hole / e Å^-3^ | 0.34/-0.38 |
| Flack parameter | -1.1(16) |


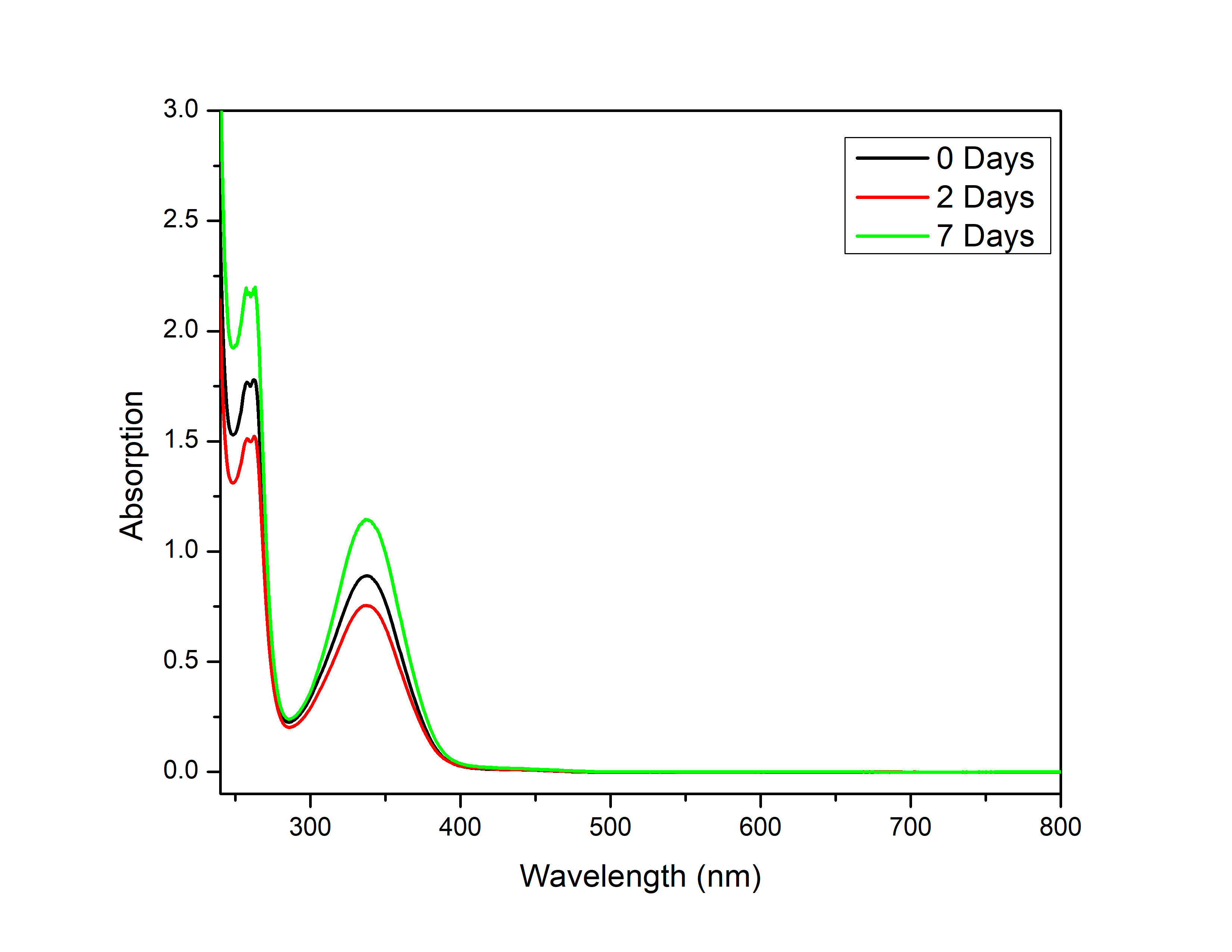


**Figure S46:** UV spectra for the stability study of **HL**


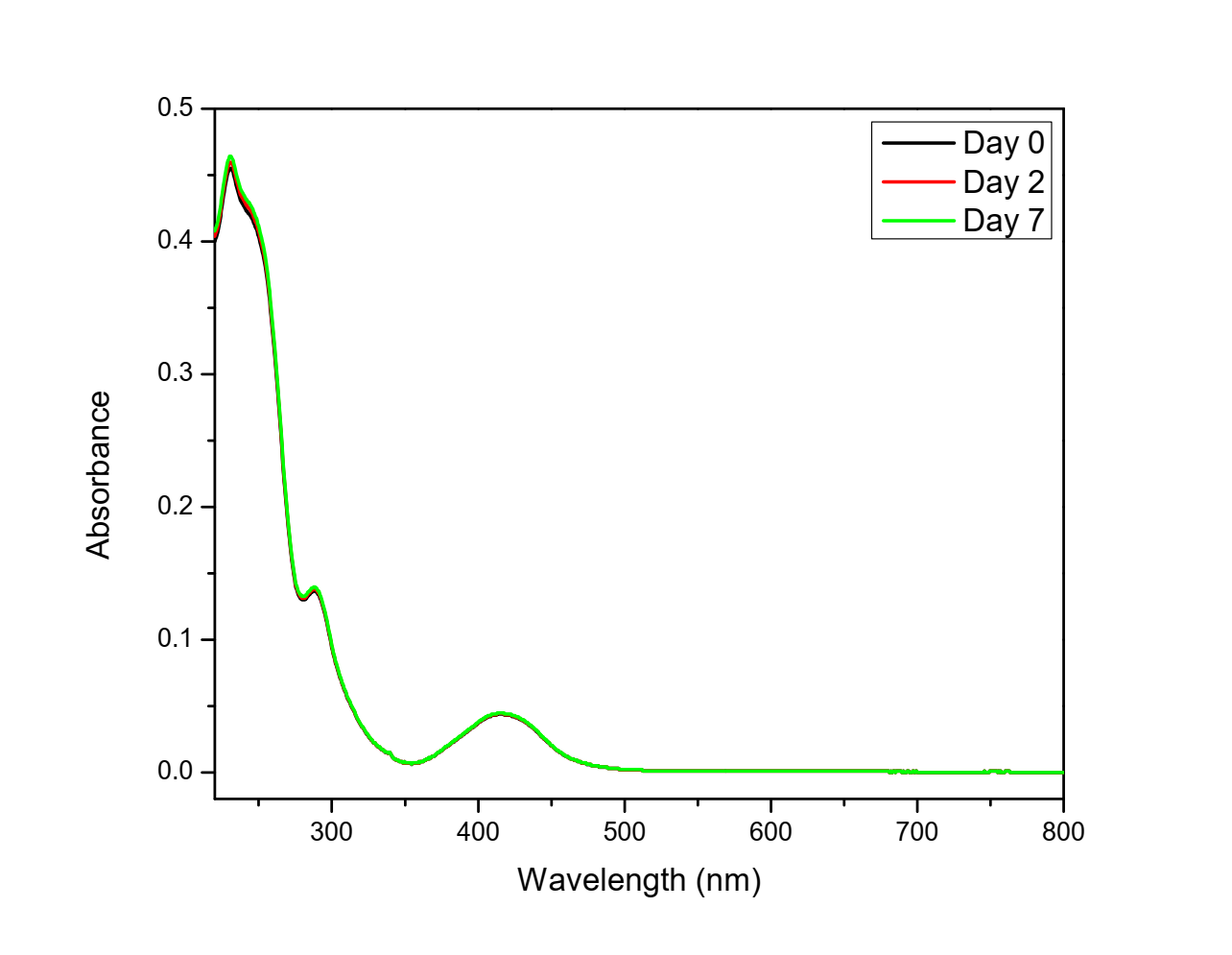


**Figure S47:** UV spectra for the stability study of **CoL_2_**


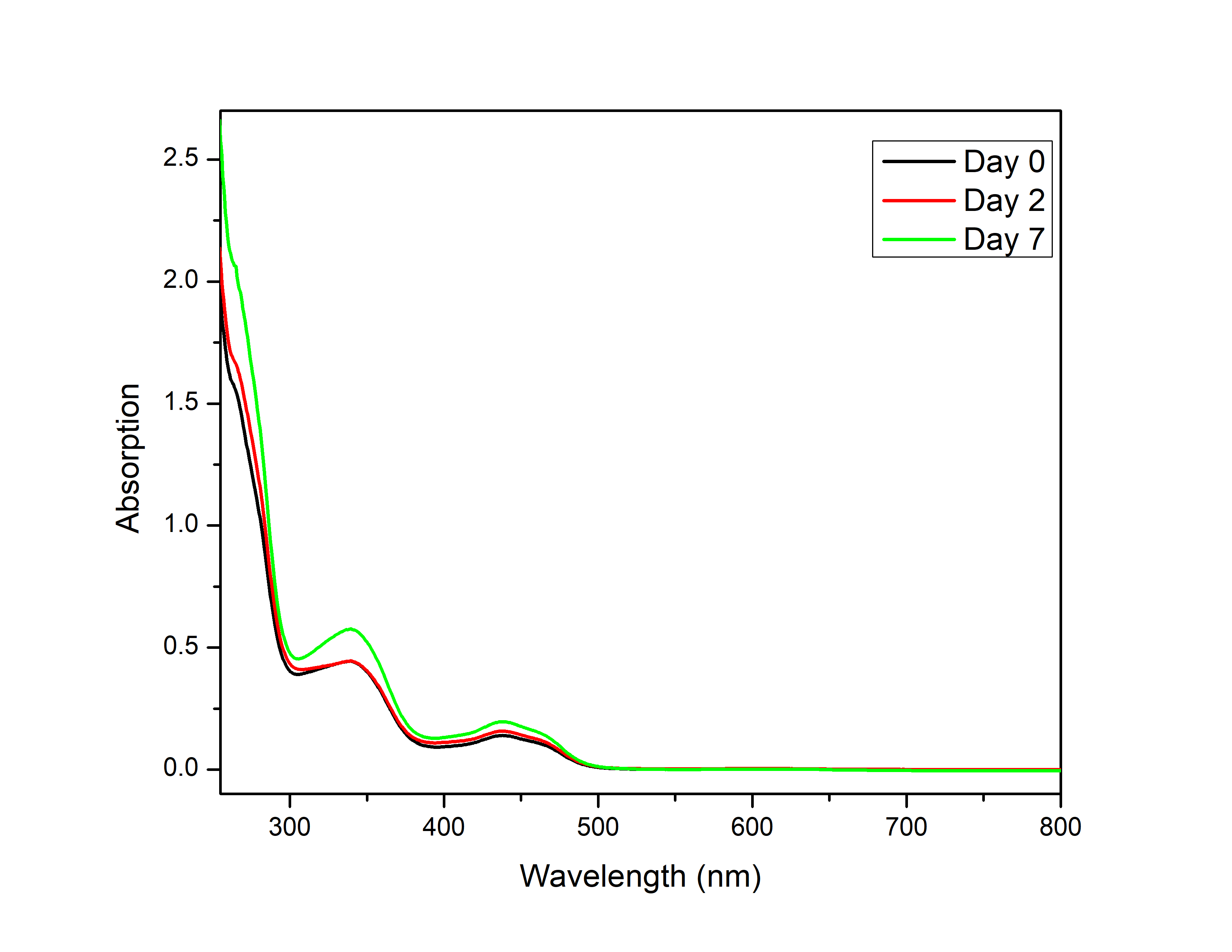


**Figure S48:** UV spectra for the stability study of **NiL_2_**


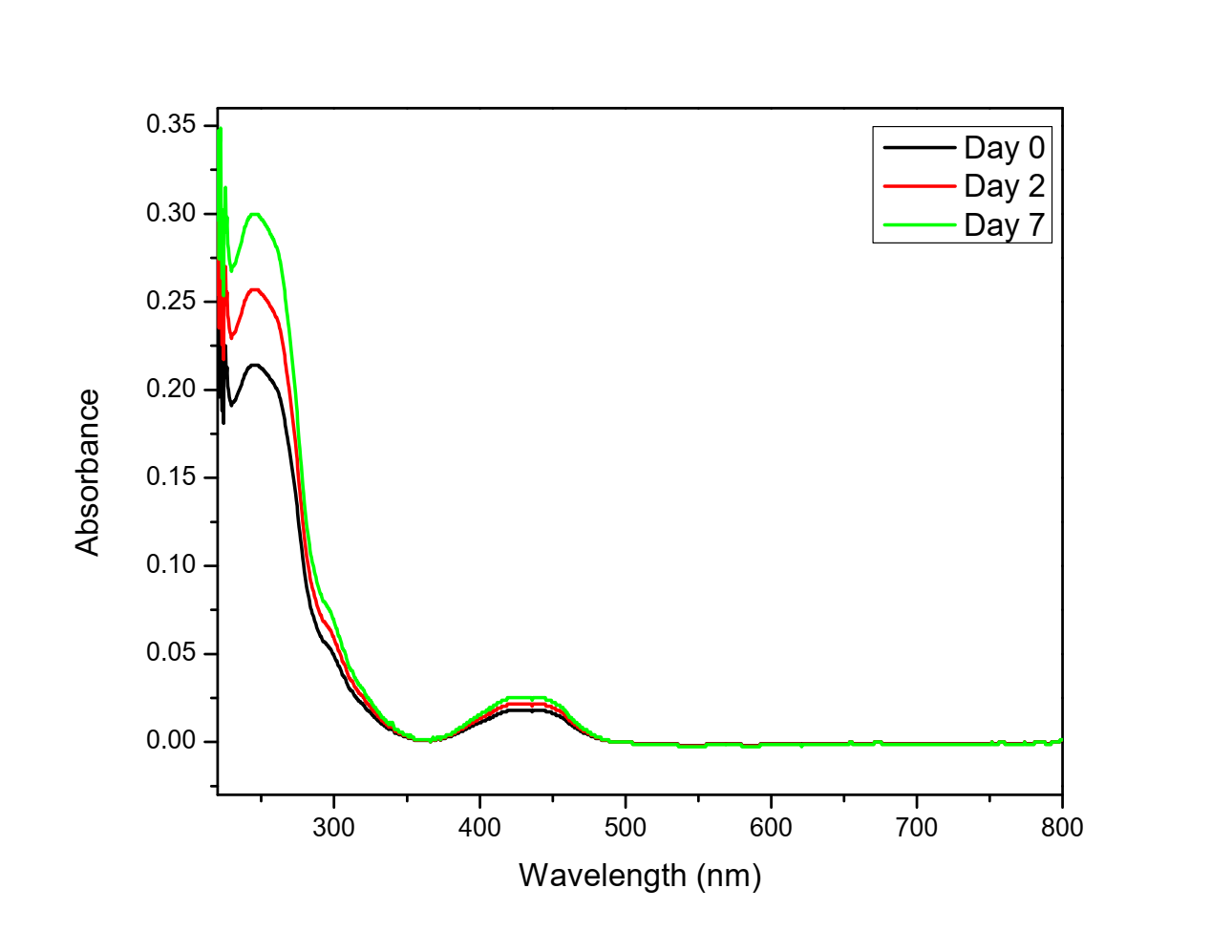


**Figure S49:** UV spectra for the stability study of **CuL_2_**


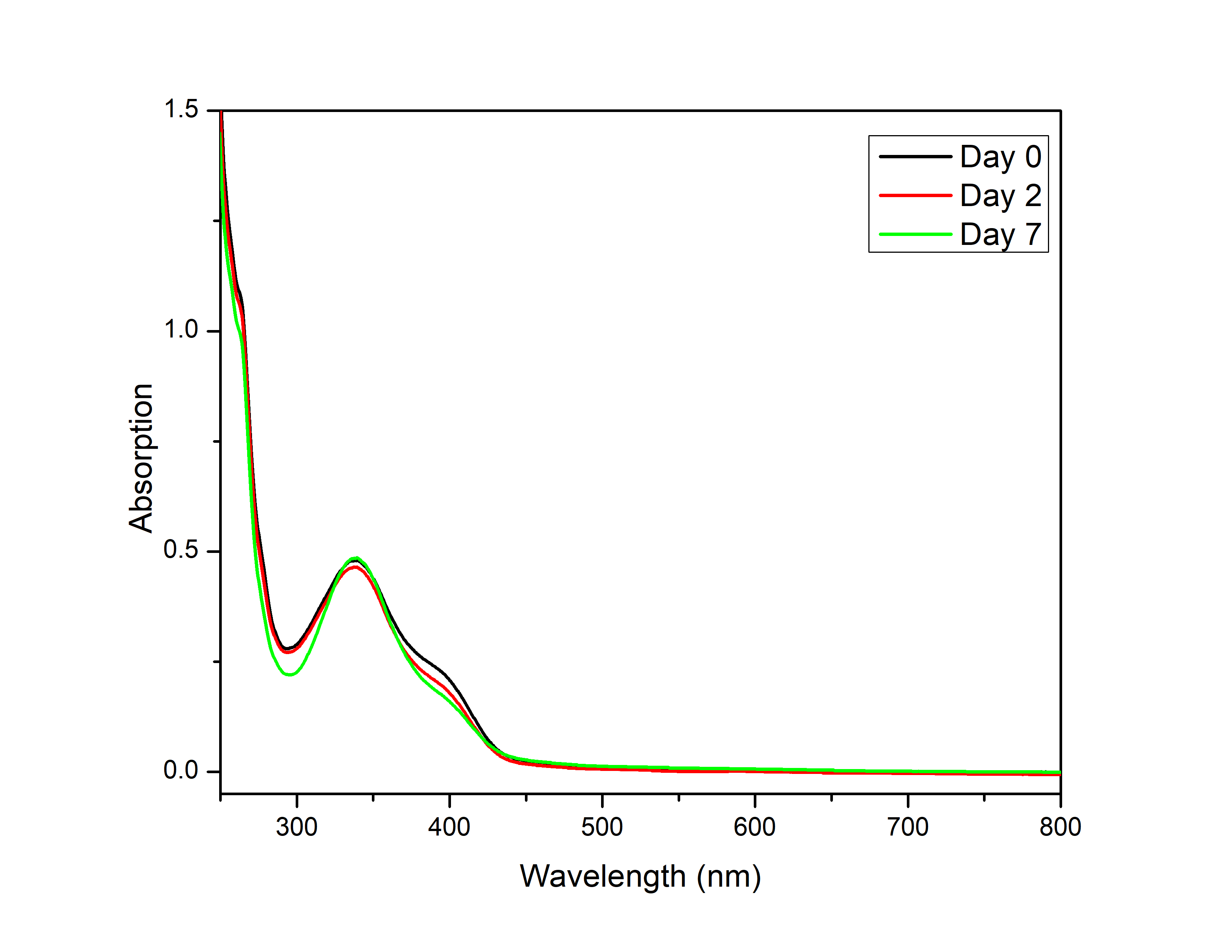


**Figure S50:** UV spectra for the stability study of **ZnL_2_**

**Figure S51:** Plot of mole ratio determination (Job’s Method) for **CoL_2_** complex

**Figure S52:** Plot of mole ratio determination (Job’s Method) for **NiL_2_** complex

**Figure S53:** Plot of mole ratio determination (Job’s Method) for **CuL_2_** complex

**Figure S54:** Plot of mole ratio determination (Job’s Method) for **ZnL_2_** complex


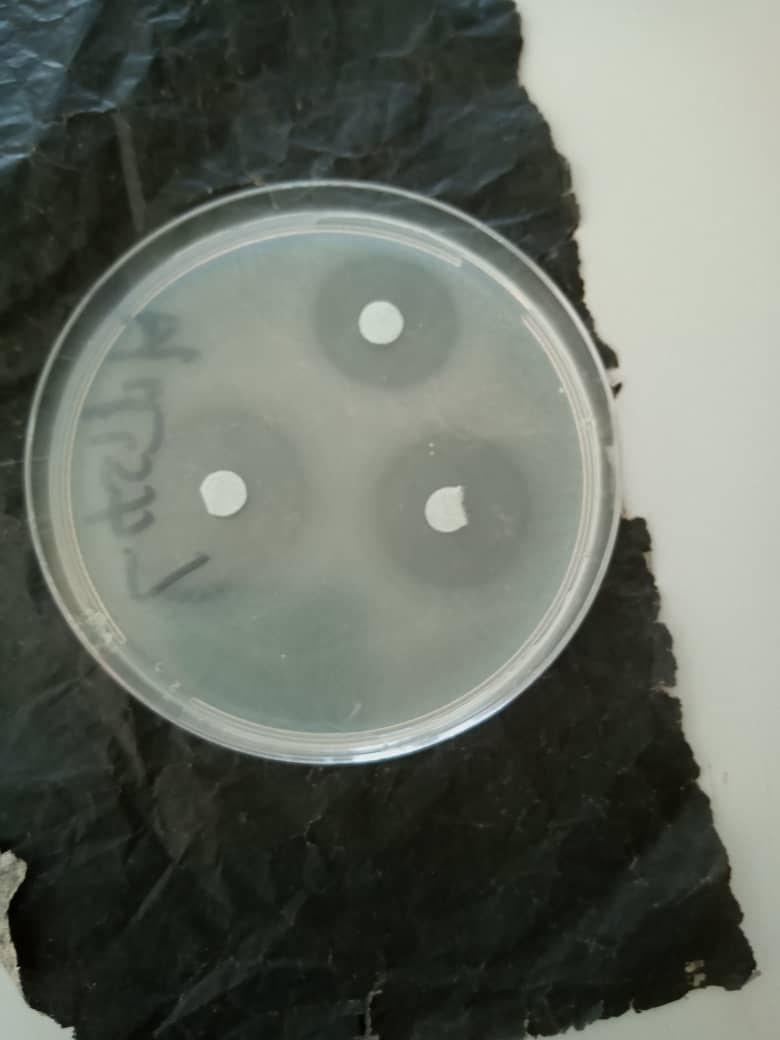


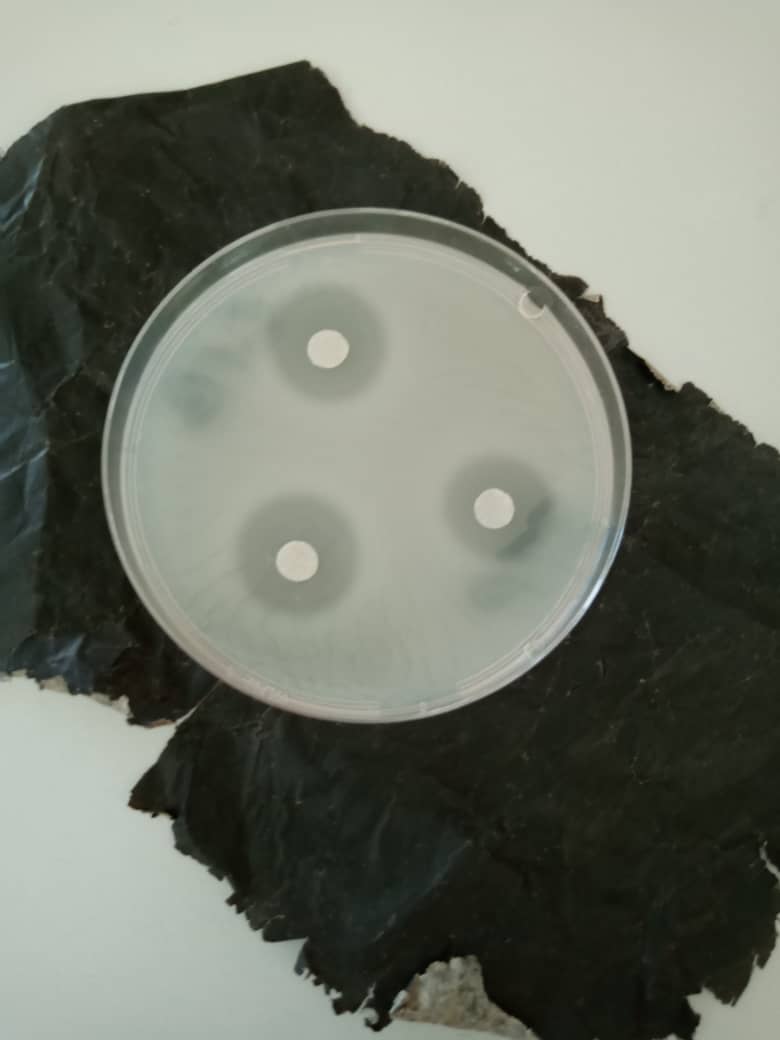


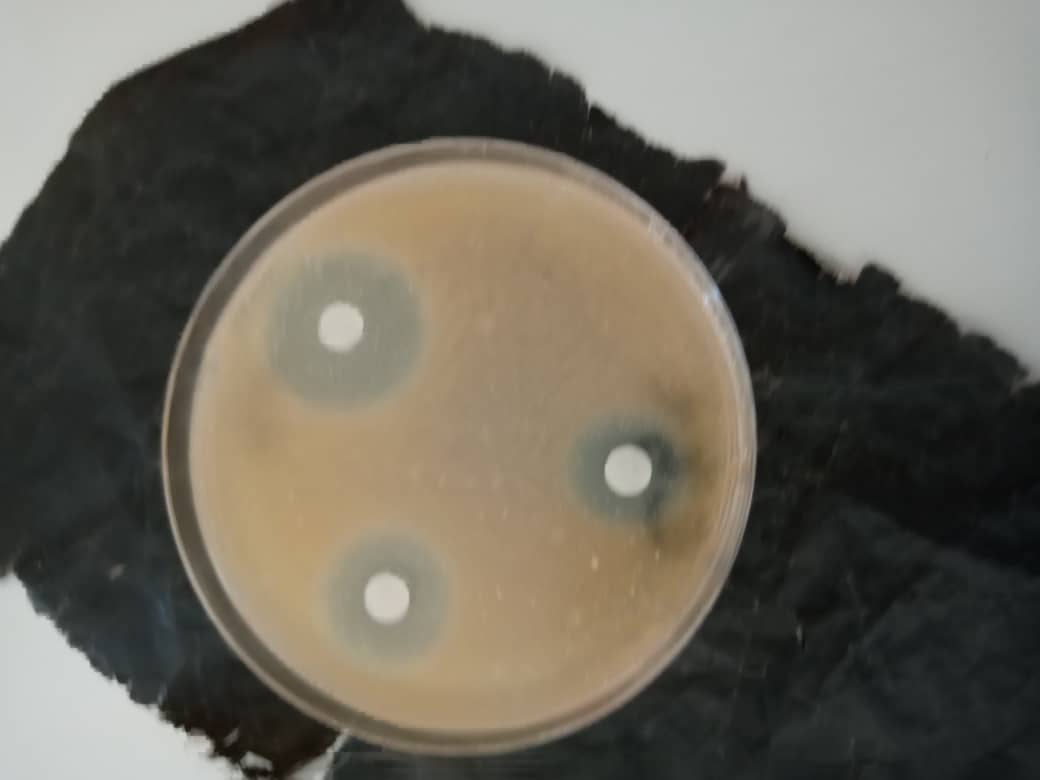


**Figure S55:** Representative of the culture plates for the disc diffusion method assay


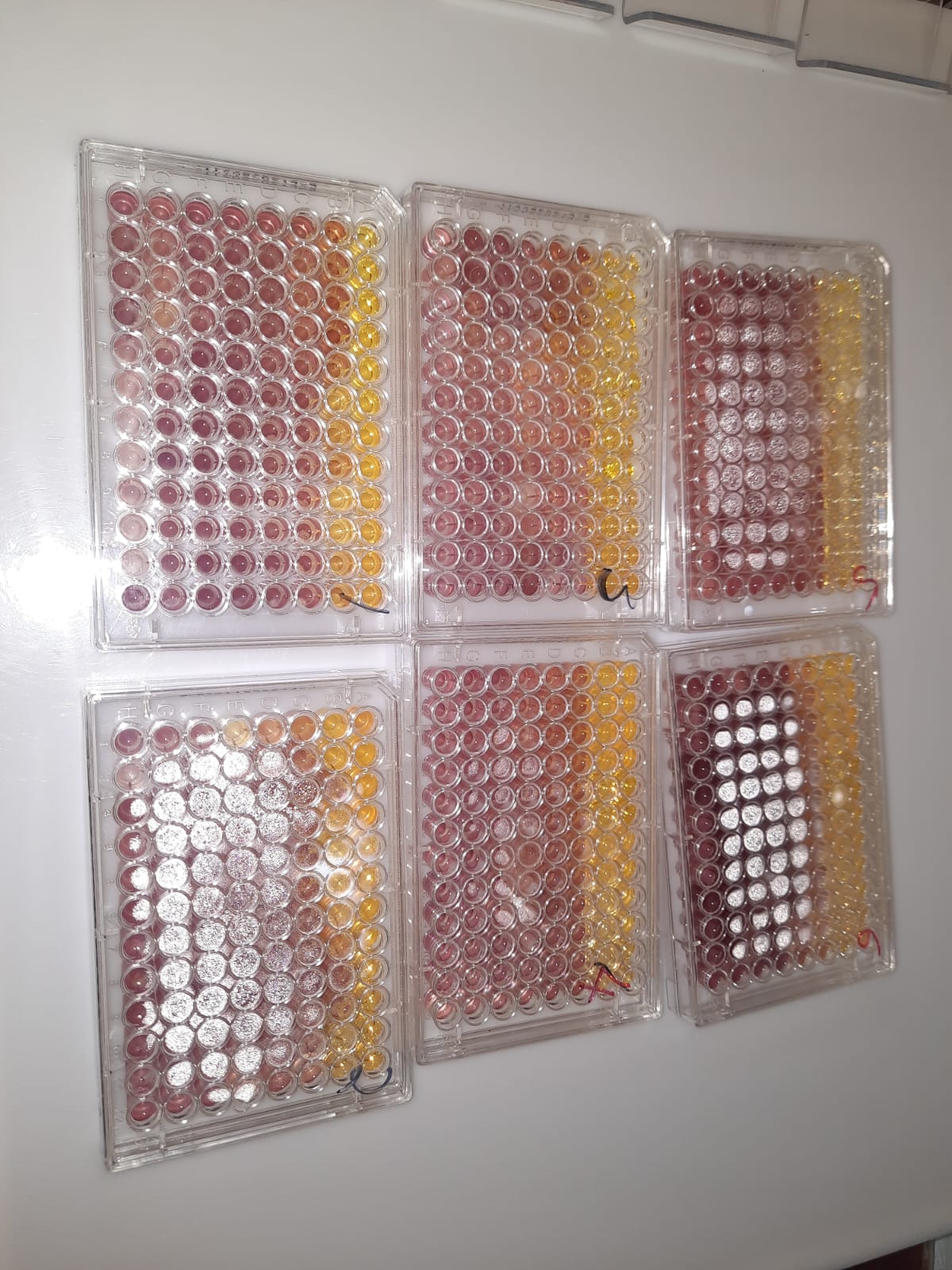


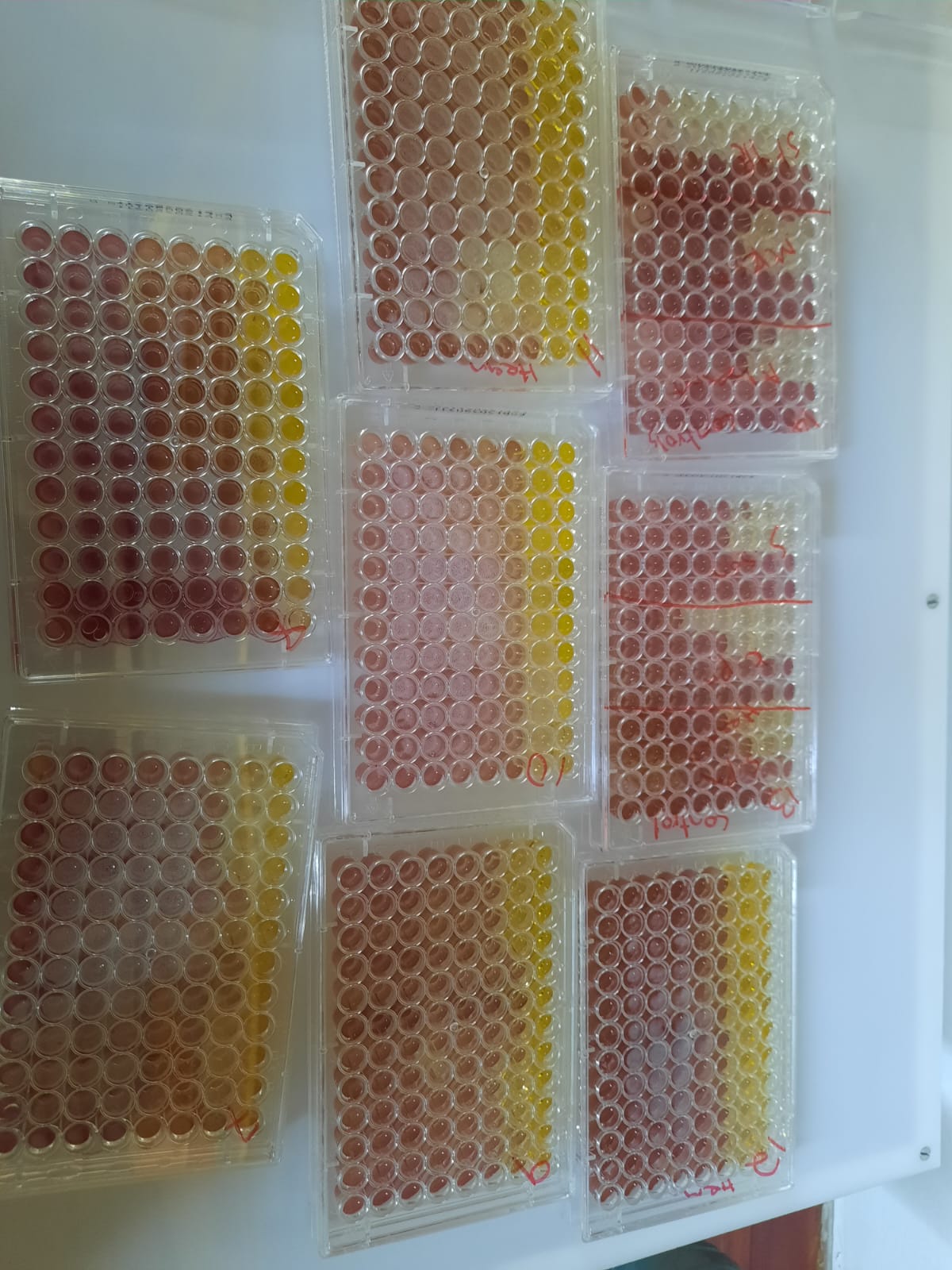


**Figure S56:** Representative of the MIC plate for the broth dilution assay


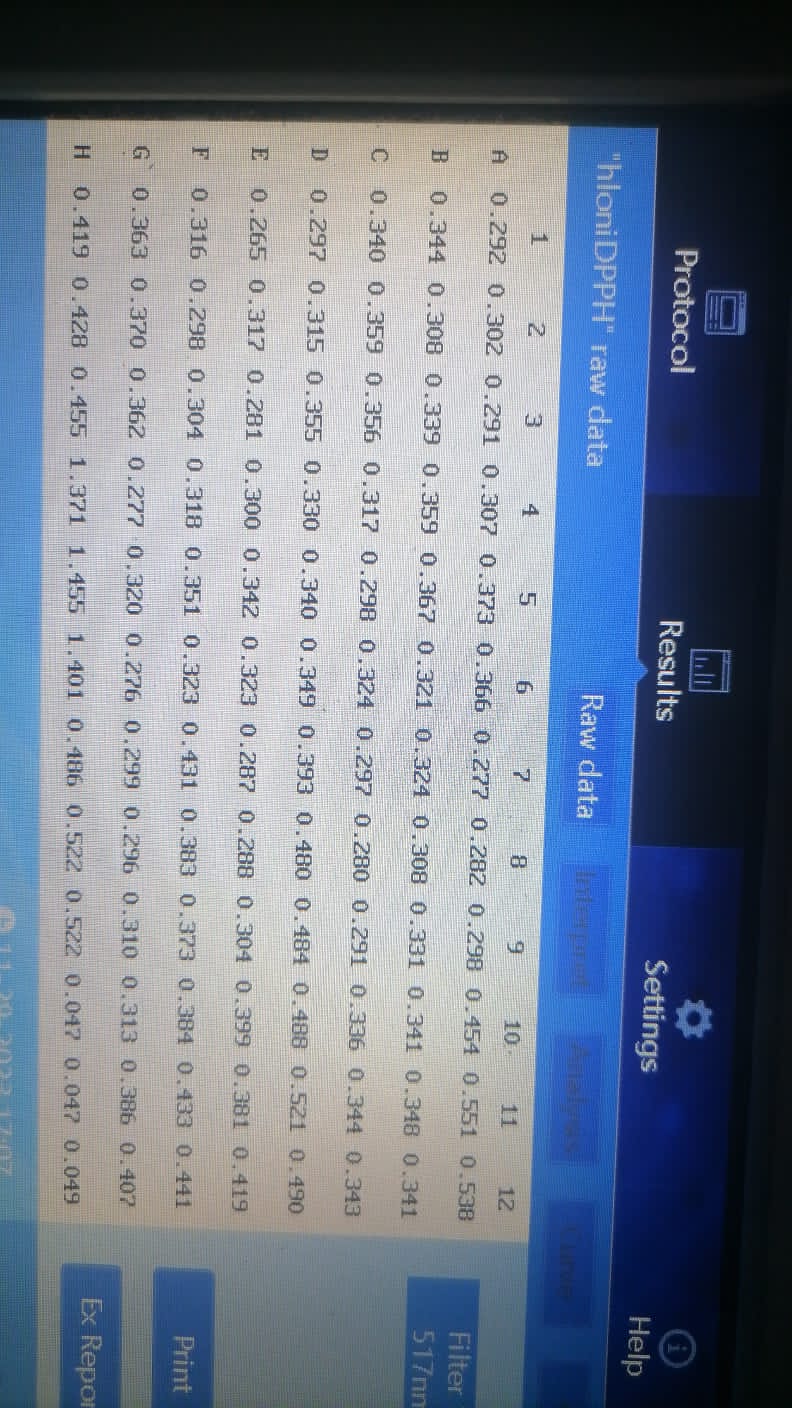


**Figure S57:** Representative of the raw data for the DPPH assay

**References**

[1] W.J. Geary, Coordination Chemistry Reviews 7 (1971) 81.

[2] K.V. Katti, Synthesis and Reactivity in Inorganic, Metal-Organic, and Nano-Metal Chemistry 35 (2005) 1.

[3] H. Kargar, R. Behjatmanesh-Ardakani, V. Torabi, A. Sarvian, Z. Kazemi, Z. Chavoshpour-Natanzi, V. Mirkhani, A. Sahraei, M.N. Tahir, M. Ashfaq, Inorganica Chimica Acta 514 (2021) 120004.

[4] H. Kargar, M. Ashfaq, M. Fallah-Mehrjardi, R. Behjatmanesh-Ardakani, K.S. Munawar, M.N. Tahir, Inorganica Chimica Acta 536 (2022) 120878.

[5] L.H. Abdel‐Rahman, A.M.M. Abdel‐Mawgoud, S.K. Mohamed, M.R. Shehata, M. Abdel‐Hameed, M.A.E.A.A. Ali El‐Remaily, Applied Organometallic Chemistry 36 (2022) e6817.

[6] I. Waziri, T.L. Yusuf, E. Akintemi, M.T. Kelani, A. Muller, Journal of Molecular Structure 1273 (2023) 134382.

[7] I. Waziri, M.T. Kelani, M.O. Oyedeji-Amusa, A.K. Oyebamiji, L.-C.C. Coetzee, A.S. Adeyinka, A.J. Muller, Journal of Molecular Structure (2022) 134756.

[8] H. Kargar, M. Fallah-Mehrjardi, R. Behjatmanesh-Ardakani, H.A. Rudbari, A.A. Ardakani, S. Sedighi-Khavidak, K.S. Munawar, M. Ashfaq, M.N. Tahir, Polyhedron 213 (2022) 115636.

[9] T. Alorini, I. Daoud, A.N. Al-Hakimi, F. Alminderej, Journal of Molecular Structure (2022) 134785.

[10] K.J. Babu, D. Ayodhya, Results in Chemistry 6 (2023) 101110.

[11] W. Al Zoubi, A.A.S. Al‐Hamdani, M. Kaseem, Applied Organometallic Chemistry 30 (2016) 810.

[12] A.C. Ekennia, D.C. Onwudiwe, L.O. Olasunkanmi, A.A. Osowole, E.E. Ebenso, Bioinorganic Chemistry and Applications 2015 (2015) 789063.
